# Metabolic novelty originating from horizontal gene transfer is essential for leaf beetle survival

**DOI:** 10.1073/pnas.2205857119

**Published:** 2022-09-26

**Authors:** Roy Kirsch, Yu Okamura, Wiebke Haeger, Heiko Vogel, Grit Kunert, Yannick Pauchet

**Affiliations:** ^a^Department of Insect Symbiosis, Max Planck Institute for Chemical Ecology, 07745 Jena, Germany;; ^b^Department of Biochemistry, Max Planck Institute for Chemical Ecology, 07745 Jena, Germany

**Keywords:** herbivorous insects, plant cell wall, plant - insect interaction, horizontal gene transfer, polygalacturonase

## Abstract

Horizontal gene transfer (HGT) provides an evolutionary shortcut for recipient organisms to gain novel functions. Although reports of HGT in higher eukaryotes are rapidly accumulating, in most cases the evolutionary trajectory, metabolic integration, and ecological relevance of acquired genes remain unclear. Plant cell wall degradation by HGT-derived enzymes is widespread in herbivorous insect lineages. Pectin is an abundant polysaccharide in the walls of growing parts of plants. We investigated the significance of horizontally acquired pectin-digesting polygalacturonases (PGs) of the leaf beetle *Phaedon cochleariae*. Using a CRISPR/Cas9-guided gene knockout approach, we generated a triple knockout and a quadruple PG-null mutant in order to investigate the enzymatic, biological, and ecological effects. We found that pectin-digestion 1) is exclusively linked to the horizontally acquired PGs from fungi, 2) became fixed in the host genome by gene duplication leading to functional redundancy, 3) compensates for nutrient-poor diet by making the nutritious cell contents more accessible, and 4) facilitates the beetles development and survival. Our analysis highlights the selective advantage PGs provide to herbivorous insects and demonstrate the impact of HGT on the evolutionary success of leaf-feeding beetles, major contributors to species diversity.

## Significance

The radiation of speciose herbivorous beetle lineages on plants correlates with the horizontal acquisition of microbial-derived enzymes targeting plant cell wall (PCW) polysaccharides, including the highly abundant pectin. However, to what extent PCW digestion is relevant for beetles’ nutrition and fitness remains unclear. We show that leaf beetles of a pectinase-null mutant line experienced severely impaired growth and survival, which could be rescued by supplementing orally a pectinase enzyme but not the pectin breakdown end product. We demonstrate that pectin digestion allows the beetles to gain access to the plant’s nutritious cell content rather than providing a carbon source. Importantly, our findings illustrate the significance of PCW-degrading enzymes for the evolutionary success of herbivorous beetles.

Based on a solid fossil record of feeding damage starting from the Devonian, plants have been eaten by insects for more than 400 My (1). Nowadays, both vascular plants and herbivorous insects constitute most of the macroscopic biological diversity (2–4). Herbivorous insects—which make up a quarter of all eukaryote species—outnumber every other plant-consuming clade of animals (5). This leads to a fundamental question in biology: which insect traits promoted their evolutionary success? Insect species richness has often been attributed to the diversity of their hostplants, a controversial correlation that depends on the specific clade and taxonomic level analyzed (6–9). Although the major evolutionary transition toward a plant-based diet remains elusive, the long-lasting interaction of insects with their hosts resulted in a plethora of morphological, physiological, and behavioral adaptations (6). Consequently, a better understanding of these adaptations would shed light on fundamental mechanisms by which herbivorous insects successfully radiated and persisted on plants.

Besides the well-known chemical defenses of plants against pathogens and herbivores (10), structural defenses like the plant cell wall (PCW) make it difficult for an insect to access essential nutrients (11).Variations in PCW density and composition affect food choice and performance of herbivorous insects (12, 13). In turn, survival on a fiber-rich but nutrient-poor diet requires adaptations such as strong mandibles, compensatory feeding, or associations with nutritional symbionts (14–16).

The PCW is difficult to digest because it is a composite material made of interconnected polysaccharides (17, 18). The primary wall is dominant in growing plant tissues and consists of a network of cellulose and hemicelluloses embedded in a pectin matrix (19). Pectin is a hetero-polysaccharide with a backbone rich in galacturonic acid (GalUA) and is highly abundant in primary walls and in the cell-connecting middle lamellae (20). Besides its overall protective role in contributing to wall strength and cell–cell adhesion, pectin also mediates defense against microbial pathogens (21). To weaken the PCW, fungal and bacterial pathogens secrete an array of PCW degrading enzymes (PCWDEs), including enzymes that digest pectin, important virulence factors (22–24).

Pectinolytic enzymes, as an adaptation to access plant nutrients, were long thought to be restricted to bacteria and fungi (25). Insect pectinase activity and other PCWDEs were believed to be restricted to symbiotic microbes associated with their hosts (26, 27). This assumption was first challenged by the discovery of a termite-endogenous cellulase (28) and the finding that a pectinolytic polygalacturonase (PG) was encoded in the genome of the weevil *Sitophilus oryzae* (29). Since then, an increasing number of animal-endogenous PGs were identified in the genomes of Phasmatodea (stick insects) (30), Hemiptera (true bugs) (31), and Coleoptera (beetles) (32–36). Recent studies have shown that insect-derived PGs, belonging to family 28 of glycoside hydrolases (GH28) cleave the GalUA backbone either terminally (exo-PG) or randomly (endo-PG), similar to their microbial counterparts (37–39).

A fascinating aspect of insect PGs is their evolutionary origin by horizontal gene transfer (HGT). Phylogenetic analyses have suggested several independent events of microbe-to-insect HGT, consistent with the patchy distribution of PGs in herbivores (39–41). Their conservation for millions of years after initial acquisition, metabolic integration, and subfunctionalization following gene duplication suggest that PGs played a key role in the diversification of plant-feeding insects (38, 41). The positive correlation between species richness and the appearance of PCWDEs including PGs has indeed been shown recently in the Phytophaga (42), a superradiation comprising weevils, leaf beetles, and longhorned beetles (43, 44).

Despite these correlative studies, experimental evidence of the adaptive value of insect PGs is still lacking. We had previously established that the leaf beetle *Phaedon cochleariae*, possessed genes for three enzymatically active endo-PGs (*GH28-1*, *GH28–5*, and *GH28–9*) which were specifically expressed in the beetle’s gut (38, 45). Further sequencing revealed a fourth PG gene (*GH28-10*) that was previously considered a *GH28-9* allele due to high sequence similarities. We sequentially knocked out all four PG-encoding genes, to assess the importance of pectin digestion on larval growth, developmental time, and survival. We found that a decrease in PG activity correlates with a lower fitness in mutant individuals, due to their inability to efficiently digest pectin. We rescued the fitness of the PG knockout mutants by supplementing their diet with a recombinant form of their own GH28-1 enzyme. In contrast, these mutants could not be rescued by adding a pectin breakdown product in their diet. This indicates that breaking open the PCW to obtain nutrient-rich cytoplasm is the primary benefit of pectinases rather than the production of pectin digestion projects for nutrition. This is an experimental demonstration of the importance of PCW digestion to herbivore fitness. These results suggest that pectinases acquired by HGT, and PCWDEs in general, represent key innovations, in part explaining the evolutionary success of herbivorous beetles.

## Results

### *PG* Knockout Causes Less Pectolytic Activity and More Undigested Pectin.

**Fig. 1.**
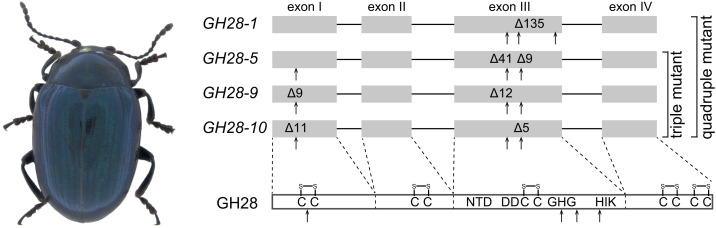
The mustard leaf beetle *Phaedon cochleariae* and its endo-PGs targeted for CRISPR-mediated knockout. The exon-intron structure of the PG encoding genes *GH28-1*, *GH28-5*, *GH28-9*, and the newly discovered *GH28-10* as well as a corresponding primary structure of a consensus GH28 protein are shown schematically. Target sites of guide RNAs are indicated with arrows and nucleotide deletions fixed in the two mutant lines (triple mutant: wild-type *GH28-1*, knockout *GH28-5*, *GH28-9*, and *GH28-10*; quadruple mutant: knockout *GH28-1*, *GH28-5*, *GH28-9*, and *GH28-10*) are shown. Conserved disulfide bridges as well as functionally important amino acid residues are highlighted in the GH28 protein using single letter code.

**Fig. 2.**
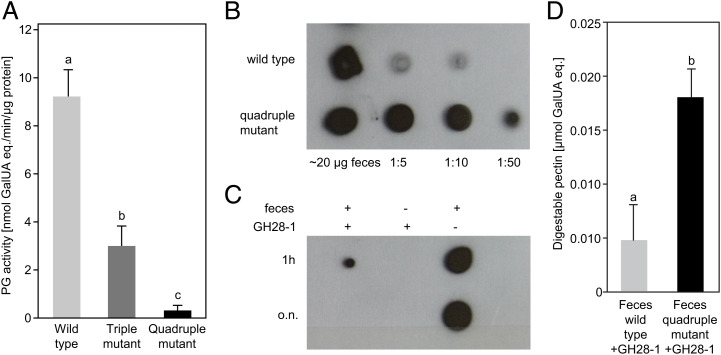
Phenotypic differences between wild-type (light gray), triple mutant (dark gray), and quadruple mutant (black) line. Different letters indicate significant differences between lines or treatments. (*A*) Quantification of PG activity from *P*. *cochleariae* larval gut-protein extracts. Larvae were reared on leaves of Chinese cabbage from supermarket (*n* = 6, *P* < 0.05; one-way ANOVA, Tukey test, *F* = 181.326). (*B*) Detection of pectin extracted from feces of quadruple mutant and wild-type larvae. Immuno-dot assay of LM20 antibody (1:250) binding to dilution series of pectin extracts. (*C*) The same fecal pectin extract from quadruple mutant larvae has been preincubated with GH28-1 for either 1 h or overnight (o.n.) before blotting. (*D*) Quantification of digestible pectin from feces of wild-type and quadruple mutant larvae after incubation with GH28-1 expressed in µmol galacturonic acid equivalents (GalUA eq.) (*n* = 3, *P* = 0.005; *t* test, t = 5.480).

Using CRISPR/Cas9, we successfully generated two *P. cochleariae* mutant lines: a triple (*GH28-5*, *GH-9*, and *GH-10*) and a quadruple (*GH28-1*, *GH-5*, *GH-9*, and *GH-10*) PG knockout line (Fig. 1 see *SI Appendix*, *SI Results* for details). Based on previous enzymatic characterization and the now confirmed endo-PG activity of GH28-10, transcriptome data, and newly generated genome data, the quadruple knockout line is equivalent to a *PG*-null-activity mutant (38). Both lines were viable under our standard rearing conditions (*SI Appendix*, Fig. S5) enabling us to test whether there was any pectin digestion in the beetles’ gut. Gut PG-activity, measured from larval gut-protein extracts, was significantly reduced to less than 50% in the triple knockout and to <2% in the quadruple knockout compared with the wild-type line (Fig. 2*A*). These results showed that most or all gut PG activity is due to several beetle enzymes and that these were all targeted in this experiment. The residual gut pectolytic activity in the quadruple knockout line likely originates from gut microbes or digested plant material, something we have not investigated further due to its insignificant contribution compared with the beetle-derived gut PG activity. To understand how the reduction of PG-activity measured in vitro translates into the ability to digest pectin in vivo, we analyzed and compared the pectin content in wild-type and quadruple mutant line insect feces. Our immuno-dot blot assays revealed that wild-type larvae do not digest all of the pectin from the food, as a signal derived from the pectin-specific antibody LM20 was detectable from their undiluted feces pectin extract (Fig. 2*B*). However, the amount of extractable pectin from feces was much higher in mutant compared to wild-type larvae, shown by stronger signals detectable from a dilution series of the mutants’ feces extract (Fig. 2*B*). These signals were reduced by supplementing the feces extracts from mutant larvae with heterologously expressed *P. cochleariae* GH28-1 endo-PG enzyme (Fig. 2*C*). In addition, significantly higher amounts of pectin breakdown products accumulated when feces extracts from mutant insects were used as a substrate in GH28-1 enzyme assays compared to those obtained from wild type insects (Fig. 2*D*). These assays showed that a much higher amount of undigested but hydrolysable pectin was still present in the feces of mutant larvae compared to wild-type ones, illustrating the ability of insect-derived PGs to digest substantial amounts of pectin from plant material.

### *PG* Knockout Impairs Growth, Development, and Survival.

**Fig. 3.**
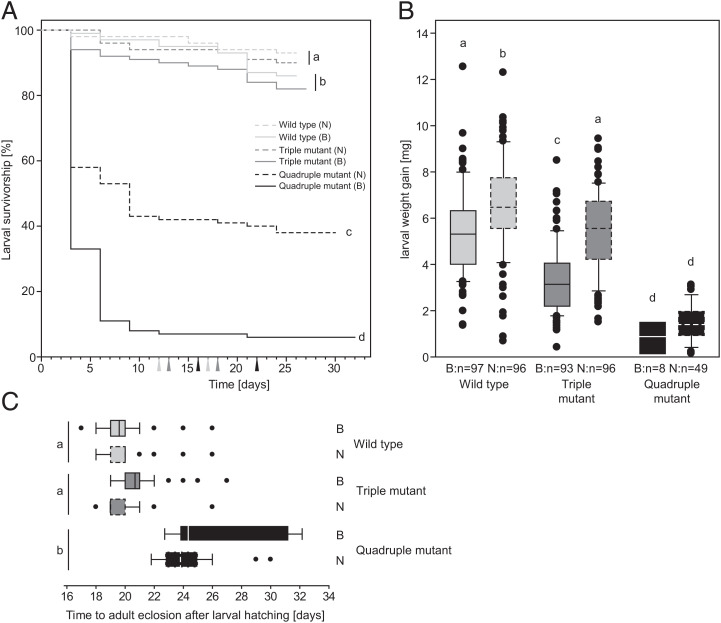
Fitness differences between wild-type (light gray), triple mutant (dark gray), and quadruple mutant (black) *P. cochleariae* lines depending on the food plants (Chinese cabbage [B, solid lines], watercress [N, dashed lines]). Different letters indicate significant differences between populations. (*A*) Kaplan–Meier survivorship curves (first instar larvae to adult eclosion). Genotypes differ in their survival (χ^2^ = 294.560, *P* < 0.001). Survival of the quadruple mutant is significantly reduced compared to wild-type and triple mutants. Survival is significantly better on watercress than on Chinese cabbage (χ^2^ = 44.506, *P* < 0.001). All *P. cochleariae* genotypes are similarly influenced by the food plants (χ^2^ = 1.341, *P* = 0.511). (*n* = 100; Cox proportional hazard models). Arrowheads on the x-axis indicate the beginning of pupation and adult eclosion in the three lines, respectively. (*B*) Larval weight gain (calculated after 7 d of feeding, 8 d old larvae). The boxes show the median (horizontal line) and interquartile range and whiskers defining the 10th and 90th percentiles. *Phaedon cochleariae* gain weight differently depending on their genotype (F = 238.954, *P* < 0.001). Wild-type individuals gain significantly more weight than triple mutant individuals. Quadruple mutants gain significantly less weight than wild-type and triple mutant individuals. In general, feeding on watercress led to a significantly higher weight gain than feeding on Chinese cabbage (F = 80.066, *P* < 0.001). However, the influence of the food plant on weight gain was dependent on the *P. cochleariae* genotype (F = 6.414, *P* = 0.002). Whereas, for wild-type and triple mutant *P. cochleariae*, feeding on watercress led to a significantly higher weight gain than feeding on Chinese cabbage, this was not the case for the quadruple mutants (two-way ANOVA, Tukey HSD). (*C*) Time to adult eclosion (starting from hatching larvae). The boxes show the median (vertical line) and interquartile range and whiskers defining the 10th and 90th percentiles. Time from larval hatching to adult eclosion was significantly different between *P. cochleariae* genotypes (deviance = −41.144, *P* < 0.001). Quadruple mutants needed significantly longer than wild-type and triple mutant individuals did. In general, the food plants did not influence the time to adult eclosion (deviance = −1.777, *P* = 0.182). That was true for all three *P. cochleariae* genotypes (deviance = −0.816, *P* = 0.665).

Next, we investigated the phenotypic consequences of the reduced ability to digest pectin. We recorded survival, development, and larval growth of our three *P. cochleariae* lines on two different food plants. We found no significant differences in survivorship from neonates to adult eclosion between wild-type and the triple mutant line individuals (Fig. 3*A*). In contrast, the number of quadruple-mutant individuals that reached adulthood was significantly reduced. Additionally, feeding on Chinese cabbage significantly reduced the survival of all *Phaedon* lines. This was most pronounced for the quadruple-mutant line where less than 10% of individuals reached adulthood when feeding on Chinese cabbage, compared with 40% on watercress.

Analyzing the impact of *PG* gene knockout on larval growth revealed a more complex pattern. We found significant differences between all three beetle lines (Fig. 3*B*). Larval growth was impaired more, when more PGs were knocked out (wild type > triple mutant > quadruple mutant). Irrespective of the beetle line, larvae grew bigger on watercress compared with Chinese cabbage. Feeding on watercress led to a significantly higher weight gain for wild-type and triple-mutant individuals than for quadruple-mutant individuals.

Furthermore, we found that these differences in larval growth correlated in part with times to adult eclosion (Fig. 3*C*). Neonates of the quadruple mutant line took significantly longer to reach adulthood compared to the other two lines (∼5 d on average, which accounts for 25% of the entire developmental time). However, the food plant did not influence the time to adult eclosion.

Taken together, the elimination of pectin digestion resulted in a reduced fitness of the PG mutant beetles, irrespective of the parameter we monitored. However, the magnitude of performance differences comparing the three lines was parameter-specific and most pronounced for larval growth. The fact that impaired growth did not result in prolonged development and higher mortality in the triple mutant line, indicated possible compensatory mechanisms, a hypothesis we investigated further.

We found gene expression differences in nontargeted *GH28* family members between triple mutant and wild-type line beetles including an elevated expression of *GH28-1* in the triple mutant line (*SI Appendix*, Fig. S6). Thus, we found evidence for successful compensation at the transcript level in triple mutant larvae that may explain their wild type-like developmental time and survival.

### PG Activity But Not Pectin Breakdown Products Rescue the *PG*-Null Mutant.

To see whether the lack of PG activity per se caused the observed fitness costs, heterologously expressed GH28-1 PG enzyme was added to the food of quadruple mutant larvae. First, we verified that the heterologously expressed GH28-1 from *P*. *cochleariae* retains activity when applied topically to Chinese cabbage leaves and in the gut after being taken up with the food. The detergent Silwet, allowing an even distribution of the GH28-1 formulation on leaf surfaces, did not impair PG activity (*SI Appendix*, Fig. S7*A*). Moreover, feeding for 24 h on leaves coated with the GH28-1 enzyme led to a measureable PG activity in the gut of quadruple mutant larvae (*SI Appendix*, Fig. S7*B*).

**Fig. 4.**
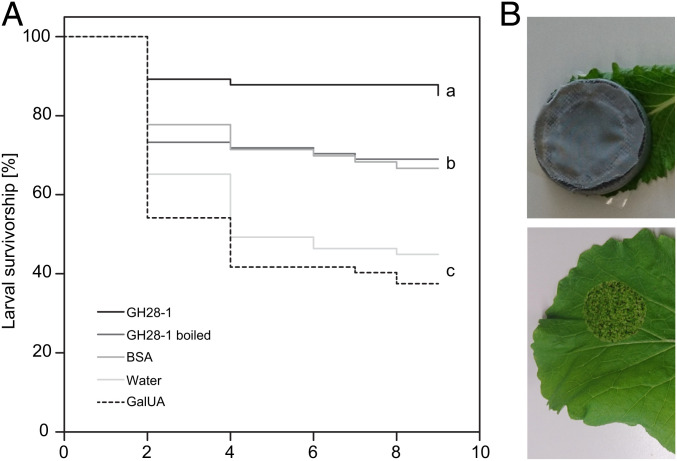
Rescue experiment by feeding different substances to the quadruple mutant population. (*A*) Kaplan–Meier survivorship curves of *P. cochleariae* for 9 d starting with first instar larvae. Larvae were reared on leaves of Chinese cabbage plants painted with GH28-1 protein (GH28-1), previously heat-inactivated GH28-1 protein (GH28-1 boiled), bovine serum albumin (BSA), water-detergent mixture used in every treatment (water), or galacturonic acid (GalUA). Larvae significantly differed in survival (χ^2^ = 45.690, *P* < 0.001). Different letters indicate significant differences between treatments (*n* = 80 for any treatment except for GalUA (*n* = 72)). (*B*) Larvae were trapped in cages and the uptake of substances was checked every 2 d by observing feeding traces.

We next set up a feeding experiment where quadruple-mutant neonates were caged to allow only consumption of PG GH28-1-coated parts of individual Chinese cabbage leaves (Fig. 4*B*). We found a strong positive effect of PG-supplemented food on larval survivorship (Fig. 4*A*). Similar to the triple mutant and wild-type line feeding on either Chinese cabbage or watercress (see Fig. 3 for comparison), the quadruple mutant supplemented with PG GH28-1 showed ∼90% survivorship. In contrast, larvae exposed to leaves coated only with a water/detergent mixture (control treatment) showed a reduced survivorship of ∼40%.

Unexpectedly, larvae feeding on leaves supplemented with the heat-inactivated PG GH28-1 or equal amounts of BSA had intermediate survival levels, which were significantly lower compared with the active PG GH28-1 treatment but higher than the control. This suggests that the beetles are obtaining some nutrition from the added protein on the leaf. However, feeding on a diet enriched with GalUA, the smallest pectin breakdown product released by PG activity, resulted in larval mortalities comparable to the control group, suggesting that the pectin breakdown product itself did not contribute significantly to beetle nutrition.

In summary, a plant diet supplemented with protein but not with a pectin breakdown product (GalUA) results in a partial rescue of the *PG*-null mutant. Furthermore, only the addition of PG activity to the leaf material can rescue the mutant line to a comparable level as wild-type beetles, which is direct evidence that the lack of PG activity alone was responsible for reduced larval survival.

## Discussion

What is the physiological benefit of the high pectinase activity provided by several *GH28* genes in the guts of herbivorous beetles? Recent data on the persistence and diversification of *GH28* genes following their HGT-based convergent acquisitions in different herbivorous insect lineages suggest a long-term evolutionary benefit (39–41). Although the ecological relevance of PCW degradation by phytopathogens in assisting host invasion and digestion has been known for a long time (46, 47), comparable evidence for insect herbivores is lacking (48). A few studies have down-regulated the expression of genes encoding cellulases (49, 50), a mannanase (51) or PGs (52) by RNA interference in various species of leaf beetles, assigning specific PCWDEs to broad digestive abilities. However, even when taking into account the complexity of PCWDE gene families by RNA interference of several genes simultaneously, strong phenotypes such as increased insect mortality were not observed (52).

Here, using a CRISPR/Cas9 knockout approach, we show that the loss of PG activity results in strong negative fitness consequences in an herbivorous beetle. This provides the evolutionary rationale for the adaptive value of retaining PG genes and metabolically integrating the corresponding enzymes after horizontal acquisition. Earlier studies employing RNA interference did not show strong fitness effects because residual enzyme activity resulting from incomplete expression knockdown was sufficient to confer a significant benefit, as we saw when only three *PG* genes were completely knocked out.

The high mortality caused by the complete loss of PG activity convincingly shows the significance of pectin digestion for *P*. *cochleariae*. Evidence from other leaf beetles is consistent with our results. The tortoise beetle *Cassida rubiginosa* is unable to digest pectin and has lower survivorship when its symbiotic bacterium expressing pectinases is removed (53). Supplementing the diet of the bean beetle *Callosobruchus maculatus* with PG inhibitors (54) also caused reduced larval survival. Thus, a similarly important role can be attributed to pectin digestion in other herbivorous beetles and herbivorous insects housing PG enzymes in general.

The single functional GH28-1 endo-PG in the triple mutant was sufficient to compensate for the knockout of its counterparts in our experimental setup. This was further supported by the rescue of the quadruple mutant when fed with leaves supplemented with functional GH28-1 enzyme. However, PG activity is not encoded by a single gene, but by a gene family in leaf beetles and other insects with some members having functional overlap (38, 55). Thus, the question arises about the factors causing the evolutionary stability of redundant *GH28* genes other than dosage effects. Similar to beetles, phytopathogenic fungi possess moderate-size *GH28* gene families that encode proteins with overlapping functions (56). However, although fungal PG enzymes are major virulence factors, individual PG-mutants rarely lose pathogenicity (23). Instead, the functional redundancy of fungal PGs has been tied to an evolutionary arms race with PG inhibitors (57), proteins encoded by huge gene families in plants to defend themselves against microbial invaders (58). One specific PG inhibitor possesses variable binding affinities toward different endo-PGs of a single fungal species (59). Our recent analyses of the differential binding and inhibition of *P. cochleariae* endo-PGs by plant-derived PG inhibitors indicate that a similar coevolutionary arms race may be occurring in the beetle-plant interaction (60).

A diverse set of pectolytic enzymes may provide leaf-feeding beetles with increased opportunities for adaptation but at the same time impose certain limitations. On the one hand, it enables a robust response to a dynamic environment, including changes in food-plant species composition, organ and developmental stage-specific PCW pectin concentrations and remodeling of the PCW due to abiotic stress like global warming and its multifactorial effects (61). It is generally accepted that genetic redundancy resulting in functional compensation can contribute to the genetic robustness of a system, providing a selective advantage (62–64). On the other hand, since not all herbivorous insects (e.g., grasshoppers, lepidopteran, and dipteran larvae) can digest pectic polysaccharides, leaf beetles may now be so dependent on pectin digestion that they have lost other ancestral mechanisms for coping with mechanical defenses of plants to guard their nutritious cytoplasm from herbivores. This dependency may explain why all investigated Phytophaga species possess PG genes except for those that have secondarily shifted to a diet that lacks pectin (e.g., fungus weevils and ambrosia beetles) or that feed on grasses low in pectin, relying on bacterial symbionts to provide PG activity (some reed beetles) (42, 65). Thus, a robust and adjustable pectin digestion may promote but also determine the beetle’s ability to adapt to a changing environment, a fact that may apply to the Phytophaga clade in general—a major lineage contributing to various ecosystem service roles including weed biocontrol agents, pollinators and decomposers.

Whether PGs contribute to the efficient digestion of PCW, by making the nutritious cell content available, or whether the pectin breakdown product GalUA can be directly utilized, has been an open question since insect PGs were first discovered ∼20 y ago. The results of our rescue experiments provide evidence that PGs contribute to unpack the plant cells to fuel other digestive processes rather than providing the beetle with GalUA as an energy source. These findings are further supported by the inability of any animal to catabolize GalUA. Such an important function of PGs at the beginning of the digestive process correlates well with PG expression patterns in the beetle’s anterior midgut cells (29), and with where PGs from pectolytic symbionts enter the beetle’s digestive tract, at the foregut/midgut junction (53). Nevertheless, as leaf beetles are associated with a diverse microbiota (66), probably including GalUA-metabolizing bacteria or fungi, pectin breakdown products released in the beetle’s gut may be beneficial to the beetle by contributing to maintain a healthy gut community.

Our study underscores the importance of horizontal gene transfer in driving metabolic innovations such as pectin digestion in herbivorous beetles. We found that the main benefit of PG activity is the efficient release of nutrients. This is the experimental demonstration of the importance of PCW digestion to herbivore fitness and the selective advantage provided by the pectolytic system in surviving and diversifying on a plant-based diet.

## Materials and Methods

### Leaf Beetle Rearing and Testing on Plants.

*Phaedon cochleariae* was reared for several generations in the laboratory (16 °C, long day conditions, 16-h/8-h light/dark period) on Chinese cabbage (*Brassica rapa* ssp. *pekinensis*) leaves obtained from a supermarket. Cabbage plants used for bioassays (*B. rapa* ssp. *pekinensis* var. Cantonner Witkropp) were reared in the greenhouse (21 °C, 55% humidity, long day conditions, 14-h/10-h light/dark period), and larvae were fed with middle-aged leaves from 6- to 8-wk-old nonflowering plants (rescue experiment) or with entire plants (fitness recordings). Watercress (*Nasturtium officinale*) was reared in the greenhouse (21 °C, 60% humidity, short day conditions, 10-h/14-h light/dark period). Larvae were fed with approximately 8-wk-old watercress.

### Genome Sequencing and Assembly.

A pool of three wild-type pupae was used to isolate high molecular weight (HMW) genomic DNA with the Nanobind Tissue Big DNA Kit (Circulomics). The resulting DNA was treated with the Short Read Eliminator Kit XS (Circulomics) to selectively precipitate HMW fragments. Final DNA purity and concentrations were measured using Nanodrop (Thermo Fisher) and Qubit (Thermo Fisher). Sequencing libraries were constructed using the HMW DNA as input for the Nanopore LSK-109 ligation kit (Oxford Nanopore Technologies) following the manufacturer’s protocol. A total of 2.54 million reads (32.85 Gb) were generated from one R 9.4.1 MinION flow cell and bases were called by GUPPY v.4.0.11 (67) with high-accuracy option (dna_r9.4.1_450bps_hac.cfg model). The N50 length of the called sub reads was 26.71 kb. The draft genome was assembled using Flye 2.8.3 (68) with setting minimum overlap as 10 kb and with “-meta” option. The generated assembly was four times polished with Racon v.1.4.13 (69) with (-m 8 -x -6 -g -8 -w 500) option and then further polished one time with Medaka v.1.0.3 with the r941_min_high_g344 model using the MinION raw reads. Diploid regions were purged with PURGEhaplotigs v.1.0.3 (70) resulted in the final genome assembly, which had N50 of 432 kb and 1.45 Gb of total genome size. The quality of the assembled genome was assessed by BUSCO ver.4 (71) with insecta_odb10 database, and the final genome assembly covered 92.0% of complete BUSCOs with having 2.4% fragmented and 5.6% missing BUSCOs.

### Transcriptome Sequencing and Assembly.

For full-length complementary DNA (cDNA) sequencing using the PacBio ISO-Seq method, total RNA was isolated from 10 individual larvae and adults, respectively. RNA extraction was performed using the innuPrep DNA/RNA Mini Kit (Analytik Jena) following the manufacturer’s instructions. RNA integrity was checked on an Agilent 2100 Bioanalyzer (Agilent). Equal amounts of total RNA from larval and adult samples were pooled and sent to the Max-Planck Genome Center Cologne (https://mpgc.mpipz.mpg.de/home/) for poly(A)+ mRNA isolation, SMRTbell library generation, and sequencing on a PacBio Sequel instrument. Using the Iso-Seq analysis pipeline implemented in the PacBio SMRT Link 5.1 software, the *P. cochleariae* mRNA data processing resulted in the assembly of 20,835 high quality transcripts. The HQ transcripts were annotated using BLAST, Gene Ontology, EggNOG, and InterProScan in OmicsBox v2.0 (https://www.biobam.com/omicsbox). For BLASTx searches against the nonredundant NCBI protein database (nr database), up to 20 best NR hits per transcript were retained, with an E-value cutoff of ≤10^–3^ and a minimum match length of 15 amino acids.

### Single Guide RNA Design, CRISPR/Cas9 Complex Assembly, In Vitro Test, and Egg Injections.

Cas9 endonuclease requires two RNA motifs for functional editing, a target-specifying CRISPR RNA (crRNA) and a transactivating crRNA (tracrRNA). The crRNAs targeting the *GH28-1*, *GH28-5*, and *GH28-9* genes were designed using ZiFit Targeter version 4.2 tool (72). The output sequences of 20-nt length were tested for their specificity by using our in house transcriptome of *P*. *cochleariae* as a searchable database allowing no mismatches (*SI Appendix*, Table S1). crRNAs and tracrRNA were synthesized by Integrated DNA Technologies (IDT), and the Cas9 endonuclease was purchased from IDT. The CRISPR/Cas9 ribonucleoprotein complex (RNP) was prepared by mixing 50 pmol Cas9 nuclease with a 50 pmol tracrRNA:crRNA duplex in duplex buffer (IDT) in water followed by a 10-min incubation at room temperature. The ability of each RNP to specifically cleave its target was tested in vitro by using full-length PCR products as substrate. 250 fmol of DNA was incubated with 1,250 fmol RNP in Nuclease Reaction Buffer (NEB) at 37 °C for 60 min. Reaction products were resolved on a 1.2% agarose gel after incubating the mixture with 20 µg of Proteinase K (Qiagen) at 56 °C for 10 min. Mixtures of RNPs were injected in freshly laid eggs using the FemtoJet (Eppendorf SE) with borosilicate capillary needles that were pulled by a P-2000 micropipette puller (Sutter Instrument). Eggs were kept on moistened paper at 20 °C for 2 d and thereafter on dry paper until larval hatching.

### Genomic DNA Isolation, Genotyping, and Crossings.

Genomic DNA was isolated from the terminal tarsomere of a leg from third instar larvae. Each tip was put in 100 µL of a suspension of 10% Chelex 100 resin in water (wt/vol) (Bio-Rad) including two metal beads in a 1.5 mL reaction vial. Mixtures were homogenized in a TissueLyser LT (Qiagen) at 40 Hz for 4 min. Homogenates were heated at 95 °C at 600 rpm for 30 min in a Thermomixer (Eppendorf SE). After pulse vortexing, samples were frozen overnight at −20 °C. Thawed supernatant containing the genomic DNA was used for genotyping by either IDAA (Indel Detection by Amplicon Analysis) or sequencing (direct or after cloning). IDAA was performed as described previously with slight modifications (73). Triprimer PCR to label the amplicons with 6-FAM was set up as follows: 12.5 µL Q5 High-Fidelity 2X Master Mix, 1.0 µL genomic DNA, 1.25 µL 6-FAM-labeled primer (0.5 µM final conc.), 1.25 µL forward primer introducing the 6-FAM-labeled primer binding site (0.05 µM final conc.), 1.25 µL reverse primer (0.5 µM final conc.), 7.75 µL water. Touch-down PCR was set up as follows: initial denaturation 98 °C for 30 s followed by 12 cycles (1 °C decrease of annealing temperature per cycle) of 98 °C, 10 s; 72 °C, 15 s; 72 °C, 15 s PCR was finished with 24–26 cycles with a 61 °C annealing temperature. PCR products were purified, mixed with 0.5 µL LIZ600 size standard (ABI/Thermo Fisher) and applied to fragment analysis on ABI 3010xl sequencer (ABI/Thermo Fisher) using conditions recommended by the manufacturer. Raw data obtained were analyzed using Peak Scanner Software V1.0 (ABI/Thermo Fisher). Direct sequencing or sequencing after cloning of PCR products was done using the same genomic DNA as templates on a ABI 3010xl sequencer (ABI/Thermo Fisher). PCR was set up as follows using 2.0 µL genomic DNA, 1.0 µL primer mix, 12.5 µL Quick-Load Taq 2X Master Mix (NEB), and 9.5 µL water: initial denaturation 95 °C for 30 s followed by 35 cycles at 95 °C, 20 s; 50 °C, 30 s; 68 °C, 30 s amplicons were purified using the DNA Clean-up and Concentration Kit (Zymo) and subsequently cloned using the pCR4-TOPO TA Vector (Thermo Fisher). G0 individuals that were identified to be mosaic for target regions identified by the methods described above were mated in pools and G1 offspring was genotyped to identify individuals that carry mutations in the germline. Depending on the target, homozygous individuals were obtained by single pair mating after two to four additional generations.

Once established, triple and quadruple knockout lines were analyzed for unintended large deletions. Genomic DNA of both triple and quadruple knockouts were extracted from pooled individual samples (12 individuals for each), sequenced by MinION flow cells, and the genomes were assembled following the same protocol described in *Genome Sequencing and Assembly* section above.

The contigs harboring target *GH28* genes were identified by blastn and were aligned to the corresponding contigs of the wild type genome by nucmer version 3.1 (74). For each contig pair (KOs vs. WT), a dot plot was generated with Dot (https://github.com/MariaNattestad/dot) to search for cases of unintended large indels at the genomic region close to the PAM sequence targeted by the Cas9 protein.

In addition, a gene synteny plot was generated for each contig pair to confirm that the genomic environment of the target *GH28* regions was not disturbed apart from the intended mutations caused by CRIPSR/Cas9 manipulations. The contigs with target *GH28s* from each KO line were aligned and the flanking genes were visualized with the R package “gggenes” (https://cran.r-project.org/web/packages/gggenes/index.html). For *GH28-1* and *GH28-5*, we could only find diverged alleles in the wild-type genome assembly; therefore, we additionally sequenced other wild-type individuals and performed another assembly for this analysis.

The frequency of the newly identified *GH28-10* gene in our wild-type line was determined by Sanger sequencing individual clones of PCR products generated from genomic DNA and primers spanning intron 3. Genomic DNA was isolated from the terminal tarsomere of a leg from 96 adults using the Quick-DNA Tissue/Insect 96 Kit (Zymo Research, Germany) according to the manufacturer’s guidelines (50-µL elution volume). Diagnostic PCR was set up as follows using the Quick-Load Taq 2X Master Mix and 1.5 µL of genomic DNA isolate: initial denaturation 95 °C for 30 s followed by 35 cycles 95 °C, 20 s; 60 °C, 30 s; 68 °C, 140 s Amplicons from eight individuals were separated on a 1.2% agarose gel, bands were gel-purified using the Zymoclean Gel DNA Recovery Kit (Zymo Research) and cloned into pCR4-TOPO TA Vector (Thermo Fisher). Inserts were assigned to *GH28-9* and *GH28-10*, respectively, by sequencing several clones per band on an ABI 3010xl sequencer (ABI/Thermo Fisher). As these results confirmed intron 3 to vary in length comparing *GH28-9* and *GH28-10*, the large-scale screen was possible by just analyzing the band pattern of amplicons on agarose gels.

Sequences of primers used for genotyping are summarized in *SI Appendix*, Table S1.

### Heterologous Expression and Functional Characterization of GH28-10.

The open reading frame of GH28-10 was synthesized by the company Genscript, including codon optimization for expression in insect cells, and was subsequently cloned into pIZ/V5-His vector in frame with a C-terminal V5 epitope and a His_6_ tag. Heterologous expression was performed in Sf9 insect cells (Invitrogen) cultured in SF-900 II serum-free medium (Gibco). Cells were transfected in 6-well plates using FUGENE HD (Promega) as the transfection reagent. After 72 h, the culture medium of transfected cells was harvested, and cell debris was removed by centrifugation. Recombinant GH28 proteins were recovered by immunoprecipitation using anti-V5 agarose beads (V5-Trap, ChromoTek). After immunoprecipitation, agarose beads were resuspended in 150 μL of double distilled water. The constructs for heterologous expression of the endo-PGs *GH28-1*, *GH28-5*, and *GH28-9*, previously characterized in our laboratory (38), were transfected and used as positive controls. The proteins were detected by Western blot on an Azure 600 Imager (Azure Biosystems) using anti-V5 HRP antibody (Bethyl) and the Radiance Plus Femtogram HRP substrate (Azure Biosystems). GH28 proteins bound to anti-V5 agarose beads were incubated with pectic substrates including the de-methylated polymer polygalacturonic acid, oligomers ranging from dimer to tetramer of GalUA (all Megazyme) and a mixture of heptamer and octamer of GalUA (Elicityl). Enzyme assays (20 μL) were set by mixing agarose beads resuspended in water (14 μL) with a 1% solution of polymeric substrate (4 μL) or with a 10 µg/µL solution of oligomeric substrate (1 µL) in a 20 mM citrate/phosphate buffer pH 5.0. Enzyme assays were incubated for 16 h at 40 °C before being applied to thin layer chromatography (TLC) plates (silica gel 60, 20 × 20 cm; Merck). TLC plates were developed for 180 min in a mobile phase composed of ethyl acetate/acetic acid/formic acid/water in the ratio 9:3:1:4 and then dried at room temperature. The hydrolysis products were subsequently revealed by spraying the plates with 0.2% (wt/vol) orcinol in methanol/sulfuric acid (9:1) using a CAMAG Derivatizer (CAMAG), then heated briefly until spots appeared on the plates.

### Quantification of PG Activity.

PG activities from gut samples and incubations with GH28-1 from heterologous expression were quantified by measuring reducing groups released after pectin hydrolysis by the 3,5-dinitrosalicylic acid (DNS) method as described earlier (52, 75). De-methylated polygalacturonic acid or pectin extracts from larval frass (see below) were used.

### Expression Analysis of PG Family Members.

To compare gene expression in wild-type and triple knockout larvae, reverse transcription quantitative PCR (RT-qPCR) was performed. Each assay was set up in two technical replicates for each of the three biological replicates. RNA extraction was performed from third instar larvae (∼10 d after hatching from egg) using the innuPrep DNA/RNA Mini Kit (Analytik) following the manufacturer’s instructions. RNA integrity was checked on a 2100 Bioanalyzer (Agilent). 500 ng of total RNA from each pool was reverse-transcribed with a 3:1 mix of random and oligo-dT20 primers. RT-qPCR was performed in optical 96-well plates on a CFX Connect detection system (BioRad). All steps were performed with the Verso 2-Step QRT-PCR Kit (SYBR Green + Separate ROX Vial; Thermo Fisher) following the manufacturer’s instructions. The PCR program was as follows: 95 °C for 15 min, then 40 cycles at 95 °C for 15 s, 58 °C for 30 s, and 72 °C for 30 s, and afterward a melt cycle from 55 to 95 °C in 0.5-s increments. Primers were used from a previous study (52). Specific amplification of each transcript was verified by dissociation curve analysis. Because of their high sequence similarity, it was not possible to design primers to discriminate *GH28-9* and *GH28-10* expression level. A standard curve for each primer pair was determined in the CFX Manager (version 3.1) based on Cq values (quantitation cycle) of qPCR running with a dilution series of cDNA pools. The efficiency and amplification factors of each qPCR primer pair based on the slope of the standard curve was calculated with the help of the efficiency calculator (https://www.thermofisher.com/us/en/home/brands/thermo-scientific/molecular-biology/molecular-biology-learning-center/molecular-biology-resource-library/thermo-scientific-web-tools/qpcr-efficiency-calculator.html#/legacy=www.thermoscientificbio.com). *Elongation factor 1α* (*EF1α*; HE962191) was used as reference gene, and quantities of the genes of interest were expressed as RNA molecules of GOI/1000 RNA molecules of *EF1α*. The Cq values were determined from two technical replicates of each of the three biological replicates, and error bars indicate the SEM.

### Heterologous Expression and Purification of GH28-1.

GH28-1 was expressed in the yeast *Pichia pastoris* as previously described (76). The His_6_-tagged enzyme was purified using HisPur Cobalt Resin (Thermo Scientific) following the manufacturer’s instructions for the “Batch Method” with slight modifications. The enzyme-containing supernatant from the yeast expression was diluted with an equal volume of Equilibration/Wash Buffer and incubated with the cobalt resin for ∼2–6 h under constant stirring at 4 °C. At room temperature, the mixture was passed over 5 mL Polypropylene Columns (Qiagen), which retained the resin including captured proteins. After washing with 20 column volumes of Equilibration/Wash Buffer, a multistep elution was performed. Two column volumes of elution buffer were incubated on the column for 5 min, drained by gravity flow, and then reapplied to the column. This was repeated three times per elution for four elution fractions. All elution fractions were pooled and a buffer exchange into LiChrosolv ultrapure water (Merck) was carried out using Amicon Ultra-15 Centrifugal Filter Units (Merck) (10 kDa MWCO). The protein concentrations were determined with the Bio-Rad Protein Assay (Bio-Rad) with the help of a bovine serum albumin (BSA) standard curve. The protein solutions were frozen in individual aliquots in liquid nitrogen and stored at −20 °C until further use.

### Pectin Extraction and Immuno-Detection from Frass.

Frass (feces) from wild-type and quadruple knockout larvae was collected after they had fed on Chinese cabbage leaves. Pectin was extracted and subsequently analyzed from frass following a protocol previously developed for plant samples with some modifications (77). Freeze-dried and pulverized beetle frass was directly extracted using 50 µL of 1,2-diaminocyclohexanetetraacetic acid (CDTA) extraction buffer per mg of sample in a TissueLyser LT (Qiagen) at 50 Hz for 2 min. Homogenized samples were heated for 15 min in a Thermomixer (Eppendorf SE) at 95 °C while shaking constantly at 800 rpm. Supernatant was collected after centrifuging samples for 10 min at 10,000 × *g* and it was further used for immune-detection. Serial dilutions of pectin extracts from frass were applied to nitrocellulose membranes followed by probing the membrane using the pectin-specific antibody LM20 (Megazyme) (1:250). After probing the membrane with a goat anti-rat HRP conjugated antibody (Bethyl A110-105P) diluted 1:5,000, dot blots were developed using the SuperSignal West HisProbe Kit (Thermo Fisher).

### Bioassays on Plants.

Neonate larvae (first day after hatching), starting with 100 individuals from each line, were put on plants of Chinese cabbage or watercress to record survival, developmental time to adult eclosion, and larval growth rate. Developmental stage (larva, pupa, and adult) and number of dead individuals were recorded daily, and surviving larvae were transferred to fresh plants every 2 d. Larval weight was measured after 7 d of feeding (8-d-old larvae, early third instar) and weight gain was calculated as the difference between the weight of a third‐instar larva and the average weight of a neonate. The average weight of neonates was used, since they were too small to be weighed individually, and was calculated to be 0.14 mg (average of *n* = 100 measurements, SEM [SEM] = ±0.017 mg). Feeding experiments were stopped once the last individual became adult or died.

For the rescue experiment, 72–80 neonate quadruple knockout larvae were caged on individual leaves of Chinese cabbage (9–10 individuals per cage, leaf area 10 cm^2^) for 9 d. Each leaf area was painted with either 20 µg of purified 28-1 enzyme, heat inactivated 28-1 enzyme or BSA, 500 µg of GalUA (Sigma Aldrich) or the equivalent volume of water in a water/Silwet (Lehle Seeds) (1:2,000 wt/vol) mixture. Larvae were transferred to fresh leaves every day after their viability had been recorded. Due to their small size, watercress leaves were not suitable for this assay and the limited amount of purified enzyme limited this experiment to 9 d, not long enough to observe adult eclosion.

### Statistics.

Whether the PG-activity differed between *P. cochleariae* lines was analyzed by a one-way ANOVA followed by a Tukey's HSD test. A *t* test was used to analyze whether the amount of digestible pectin in feces differed between wild-type *P. cochleariae* and quadruple knockout mutant. These statistical analyses were performed using SigmaPlot 11.0 (Systat Software Inc.).

To compare transcript levels of different GH28 genes between wild-type and triple mutant lines, individual Student’s *t* tests were performed. In case of unequal variances, the Welch’s *t* test was applied. The false discover rate (FDR) was calculated to correct for multiple comparisons.

Cox proportional hazard models (coxph of the survival library; https://cran.r-project.org/web/packages/survival/index.html) (78) were used to investigate the influence of genotype (wild type, triple ko, quadruple ko) and food plants (cabbage, watercress) on the survival of *P. cochleariae*, and the influence of GH28-1 protein supplementation on the survival of *P. cochleariae* quadruple knockout mutants. The models were simplified by removing nonsignificant factors. *P* values were obtained by stepwise removing explanatory variables and comparison of models with a likelihood ratio test (79). Factor level reduction was applied to determine differences between groups of a certain explanatory variable (80).

To investigate whether food plant and genotype had an influence on the weight gain of *P. cochleariae* a two-way ANOVA with food plant and genotype as explanatory factors was applied. To achieve variance homogeneity data were log-transformed. Variance homogeneity and normality of the residuals were checked graphically. The Tukey HSD test was applied to reveal differences between groups of a certain explanatory factor.

In order to test whether the time needed from larval hatching to adult eclosion was different between *P. cochleariae* lines (genotypes) and dependent on the food plant Poisson generalized linear models (glm) were used. *P* values for explanatory variables were obtained by stepwise deleting explanatory variables and comparison of the more complex model with the simpler model (79). Factor level reduction was applied to determine differences between groups of a certain explanatory variable (80). These analyses were done in R 4.1.2 (https://www.R-project.org/).

## Supplementary Material

Supplementary File

## Data Availability

All study data are included in the article and/or *SI Appendix*.
