# Supplementary material for: Metabolic novelty originating from horizontal gene transfer is essential for leaf beetle survival
Source: Proc Natl Acad Sci U S A. 2022 Sep 26;119(40):e2205857119. doi: 10.1073/pnas.2205857119 (PMC9546569; doi:10.1073/pnas.2205857119)
Supplement: Supplementary File [file pnas.2205857119.sapp.pdf]

Supplementary Information for:

Metabolic novelty originating from horizontal gene transfer is essential for leaf beetle survival

Roy Kirsch*[1], Yu Okamura[1], Wiebke Haeger[1, §], Heiko Vogel[1], Grit Kunert[2], Yannick Pauchet*[1]

[1]Department of Insect Symbiosis, Max Planck Institute for Chemical Ecology, 07745 Jena, Germany. [2]Department of Biochemistry, Max Planck Institute for Chemical Ecology, 07745 Jena, Germany

[§]Present address: Department of Biology, Norwegian University of Science and Technology (NTNU), Trondheim, Norway.

*Corresponding Authors: RK, rkirsch@ice.mpg.de, 0049-3641571555; YP, ypauchet@ice.mpg.de, 0049-3641571507, Hans-Knoell-Str. 8

This PDF file includes:

Supplementary text (Results)

SI References

Figures S1 to S7

Table S1

SUPPLEMENTARY RESULTS

CRISPR/Cas9-mediated knockout of GH28 endo-PGs and characterization of gene copy number variation

Previous characterization of the *P. cochleariae* GH28 proteins revealed GH28-1, -5 and -9 to be endo-PGs hydrolyzing homogalacturonan, the polymeric backbone of pectin (1). Thus, we were aiming to knockout the three corresponding genes using the CRISPR/Cas9 system to eliminate PG activity from the beetle´s gut. Since the intron positions of beetle *GH28*s are highly conserved and known for *P*. *cochleariae GH28-2* and *GH28-4*, we used this information to identify target sites in the middle of their

exons. In order to analyze the impact of impaired PG activity, the three target *GH28* genes were knocked out in a consecutive, two-step manner. Due to the high sequence similarity of *GH28-5* and *GH28-9*, we screened for identical target sites between the two genes to knock them out simultaneously. Three crispr RNAs (crRNAs) targeting functionally important sites in exon1 and exon3 were selected (Fig. 1, Fig. S1; Table S1). Similarly, three crRNAs were selected to specifically target *GH28-1*. The functionality of pre-assembled ribonucleoprotein complexes (RNPs) were tested *in vitro* before egg injections (Fig. S1). In the first step, ~500 eggs were injected with a mixture of the three gRNA/RNPs targeting *GH28-5* and *GH28-9* simultaneously to generate a *GH28-5*/*28-9* homozygous knockout line.

Genomic DNA from G0 larval individuals was extracted to generate PCR amplicons of *GH28-5* and *GH28-9* exon1 and exon3 for Indel Detection by Amplicon Analysis (IDAA) in default of a visible phenotype indicating mosaicism (Fig. S2A, B). As none of the G0-mosaic individuals was identified to carry a mutation in both *GH28-5* and *GH28-9*, matings with a pool of G0 adults, mosaic in either of the *GH28* gene were set up. The offspring G1 was screened for inherited mutations by IDAA (Fig. S2C) followed by Sanger sequencing of PCR amplicons for IDAA-positive individuals. Whereas further crossings led to *GH28-5* homozygous knockout already in G2, a lack of wild-type signal in case of *GH28-9* IDAA, indicating a *GH28-9* homozygous knockout, was reached first in G4. The difficulty to obtain *GH28-9* homozygous knockout individuals was in line with the detection of up to four different deletions per target site in a single larva by IDAA and sequencing (Fig. S2D). This indicated the presence of an additional *GH28* sharing high sequence similarity with *GH28-9* rather than multiple alleles.

We decided to sequence the genome as well as full-length cDNAs using Oxford Nanopores and PacBio Iso-Seq sequencing methods respectively to further support the presence of an additional, so far overlooked *GH28* gene in *P. cochleariae.* Genome assembly revealed three contigs harboring *GH28-9*, with one contig containing a highly similar additional gene in close proximity to the other GH28-9 gene (Fig. S3A). Whereas contig_23458 and contig_15494 represent two haplotypes with a *GH28-9* allelic variant each, contig_23457 possesses an additional gene (*GH28-10*). This copy number variation (cnv) between individuals occurs in the same genomic environment, confirmed by the presence of identical flanking genes. In addition, we mapped the coding sequences of the *GH28s* as well as the flanking genes back to the Oxford Nanopore raw reads to exclude the possibility of assembly artefacts (Fig. S3B). This mapping approach clearly shows the presence of both *GH28-9* and *GH28-9* + *GH28-10* reads. RNA Iso-Seq further confirmed the additional *GH28-10* gene as well as its transcriptional activity. The multiple amino acid alignment of the corresponding GH28-9 and GH28-10 proteins derived from the genome assembly and the RNA Iso-Seq data highlights the high sequence similarity between the deduced amino acid sequences that can be discriminated only in 13 amino acid positions (Fig. S3C). Taken together, our *P. cochleariae* wild-type lab culture was found to contain a *GH28* cnv between individuals, either possessing nine or ten *GH28* genes. In contrast, we found no evidence of cnv for

any other *GH28*. To obtain data on the frequency of the *GH28-10* gene in our wild-type lab culture, we designed diagnostic primers spanning intron 3 that possesses a length polymorphism comparing *GH28-9* and *GH28-10* genes (Fig. S3D). Representative PCR products were cloned and sequenced verifying the lowest band (~ 1300 bp) to exclusively correspond to *GH28-10* whereas all the bands above correspond to *GH28-9* indicating allelic variability for this gene (Fig. S3E). A screening of ~ 100 wild-type beetles first, revealed about 97% of individuals possessing the *GH28-10* gene and secondly confirmed allelic variation for GH28-9 in our beetle population (Fig. S3F).

At this stage we already obtained a triple mutant and found *GH28-10* is very common in our wild-type culture. Furthermore, our reinvestigation of the *P. cochleariae* GH28s confirmed the endo-PG activity of GH28-10. In addition, the breakdown products of GH28-9 and GH28-10 are highly similar, indicating a benefit for the beetle through increased expression/ enzyme levels (i.e. gene dosage effect). However, when comparing GH28-9/10 to GH28-1 and GH28-5, breakdown products slightly differ indicating synergistic effects (Fig. S3G-J). Taking the enzymatic activity and widespread presence of GH28-10 into account, we decided to use the eggs of the *GH28-5/28-9/28-10* knockout line for the subsequent knockout of *GH28-1*. Approximately 100 eggs were injected to finally obtain a quadruple knockout line. Since the screen of G0 individuals revealed a larva with a large deletion mutation in exon3, the subsequent analysis of inheritance and homozygosity after further crossings was possible simply by agarose gel electrophoresis of the PCR amplicons (Fig. S4A).

Mutations were finally confirmed by Sanger sequencing of *GH28-1*, *GH28-5* and *GH28-9* PCR amplicons of 24 individuals from the complete knockout line. By this we not only verified the simultaneous, homozygous knockout of the three endo-PG coding genes targeted initially (Fig. S4A-D) but also confirmed the knockout of the newly identified endo-PG coding *GH28-10* (Fig. 1, Fig. S4C, D). In order to get additional confidence in the mutant genotypes, we sequenced the genomes of the triple and quadruple knockout line and analyzed the target gene environment for unintended long deletions. Previous studies in mammals revealed such long non-homologous end joining deletions that cannot be detected by amplicon sequencing as primer binding sites would be deleted (2, 3). Dot plot and gene synteny plot analyses revealed no such long deletions in the alleles fixed in our knockout lines (Fig. S4E-H).

References:

1. Kirsch R*, et al.* (2014) Horizontal gene transfer and functional diversification of plant cell wall degrading polygalacturonases: Key events in the evolution of herbivory in beetles. *Insect Biochem Mol Biol* 52:33-50.
2. Kosicki M, Tomberg K, & Bradley A (2018) Repair of double-strand breaks induced by CRISPR-Cas9 leads to large deletions and complex rearrangements. *Nature biotechnology* 36(8):765-771.
3. Shin HY*, et al.* (2017) CRISPR/Cas9 targeting events cause complex deletions and insertions at 17 sites in the mouse genome. *Nat Commun* 8:15464.

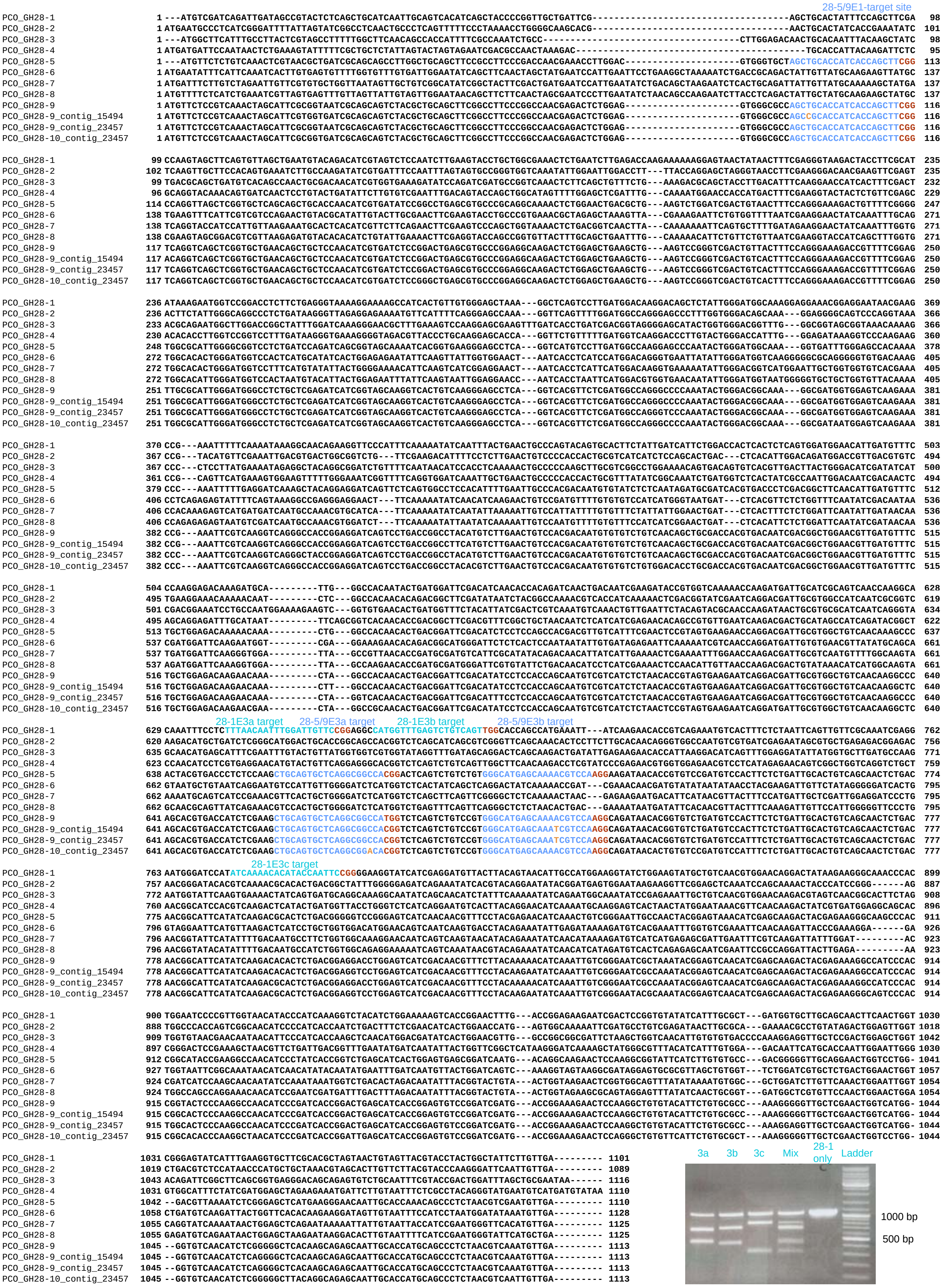

Figure S1 Multiple nucleotide alignment of *P. cochleariae GH28* coding sequences. GH28-1 to GH28-9 sequences were published previously (HE962193.1 to HE962201.1). The bottom three sequences are the ones from this study. Target sites are located in exon1 (E1) and exon3 (E3), were selected based on the previously published *GH28* sequences and are indicated in cyan for *GH28-1* and blue for *GH28-5*, *9*, *10*, respectively. PAM sites are shown in dark-red and mismatches in target sites are highlighted in orange. Functionality of sgRNAs was tested *in vitro* before injection, exemplarily shown for the digestion of the full-length PCR product of *GH28-1* by either the individual sgRNAs 3a, 3b and 3c or a mixture of them (agarose gel picture).

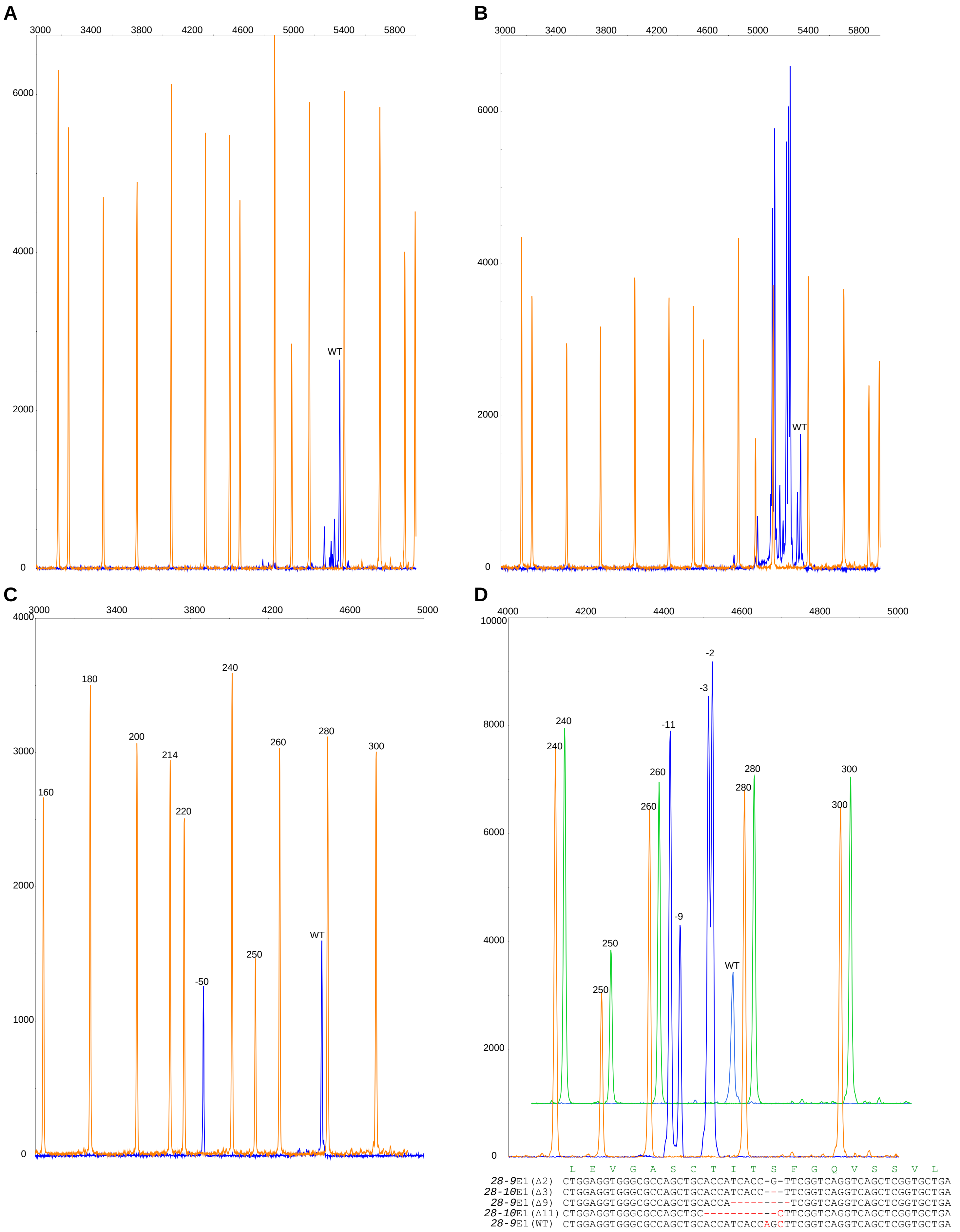

Figure S2 Indel Detection by Amplicon Analysis (IDAA) is shown by electropherograms. Each electropherogram represents the results obtained from a single specimen. Run time in seconds is indicated on the x-axis and signal intensity on the y-axis. G0 individuals were screened for mosaicism in all targeted loci shown exemplarily for A) *GH28-5* exon3 and B) *GH28-9* exon3. G1 individuals were screened for inherited mutations shown for C) *GH28-5* exon3. The detection of four different deletions in G4 individuals for *GH28-9/10* exon1 is shown in D). Wild type length of *GH28-9/10* exon1 (WT) is shown in a separate peak profile. Sequencing results and sequence asignment to either *GH28-9* or *GH28-10* are indicated below the electropherogram. Blue peaks indicate PCR amplicons and numbers of deleted nucleotides as well as wild-type peaks (WT) are indicated. The GSLIZ600 standard peaks are shown in orange (A-D) and green (D), respectively and corresponding numbers of nucleotides are indicated.

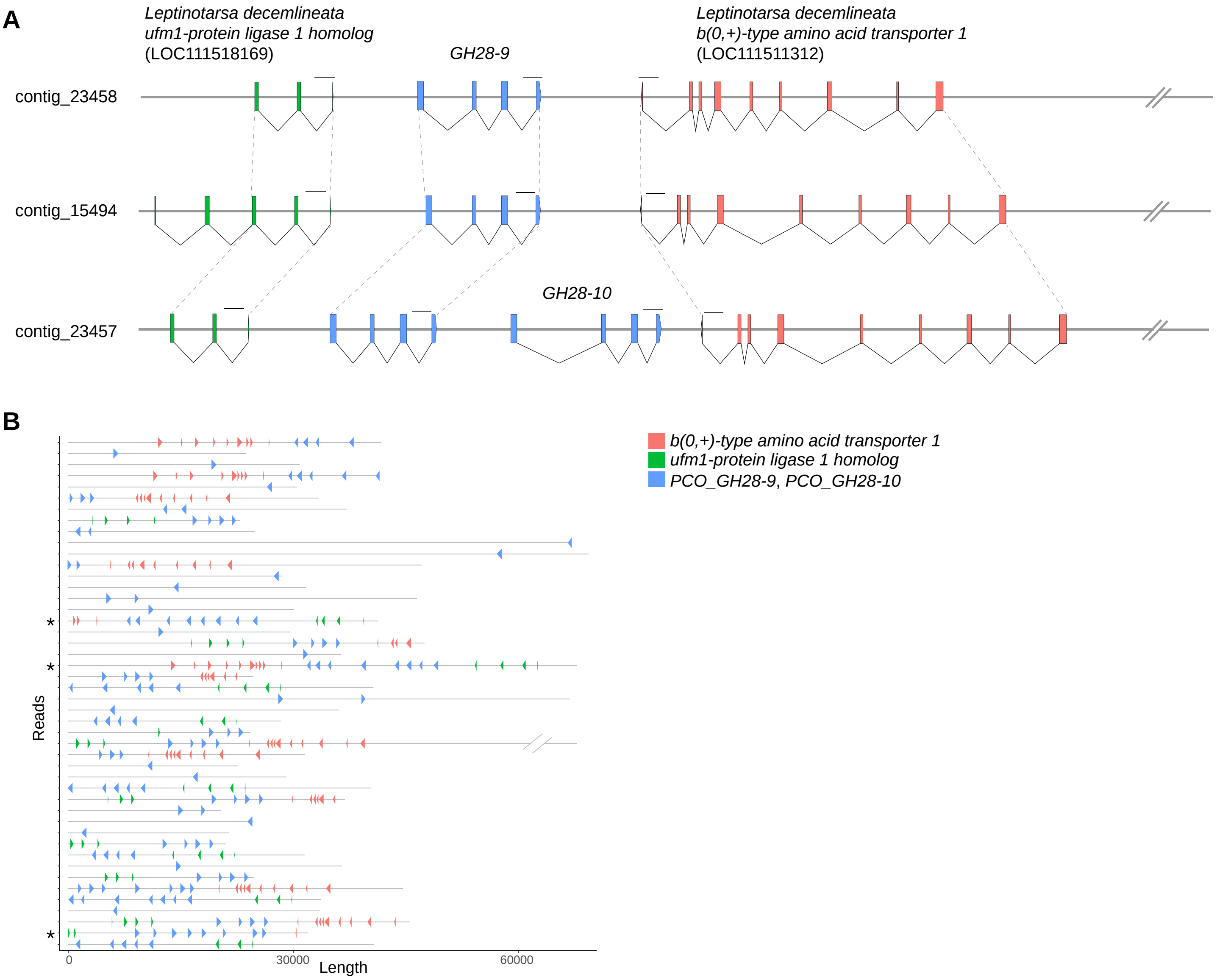

C

```
PCO_GH28-9                 1 MFSVKLAFAVIAAVYAAASAFPANETLEVGASCTITSFGQVSSVLNSCSNIVISGLSVPGGKTLELKLKSGSTVTFQGKTVFGVAHWDGPLLEIIGSKVTVKGASGHVLDGQGPKYWDGKGDGG 124
PCO_GH28-9_contig_23458    1 MFSVKLAFAVIAAVYAAASAFPANETLEVGASCTITSFGQVSSVLNSCSNIVISGLSVPGGKTLELKLKSGSTVTFQGKTVFGVAHWDGPLLEIIGSKVTVKGASGHVLDGQGPKYWDGKGDGG 124
PCO_GH28-9_contig_15494    1 MFSVKLAFVVIAAVYAAASAFPANETLEVGASRTITSFGQVSSVLNSCSNIVISGLSVPGGKTLELKLKSGSTVTFQGKTVFGVAHWDGPLLEIIGSKVTVKGASGHVLDGQGPKYWDGKGDGG 124
PCO_GH28-9_contig_23457    1 MFSVKLAFAVIAAVYAAASAFPANETLEVGASCTITSFGQVSSVLNSCSNIVISGLSVPGGKTLELKLKSGSTVTFQGKTVFGVAHWDGPLLEIIGSKVTVKGASGHVLDGQGPKYWDGKGDGG 124
Pco_HQ_transcript12018     1 MFSVKLAFAVIAAVYAAASAFPANETLEVGASCTITSFGQVSSVLNSCSNIVISGLSVPGGKTLELKLKSGSTVTFQGKTVFGVAHWDGPLLEIIGSKVTVKGASGHVLDGQGPKYWDGKGDGG 124
Pco_HQ_transcript7296      1 MFSVKLAFAVIAAVYAAASAFPANETLEVGASCTITSFGQVSSVLNSCSNIVISGLSVPGGKTLELKLKSGSTVTFQGKTVFGVAHWDGPLLEIIGSKVTVKGASGHVLDGQGPKYWDGKGDGG 124
Pco_HQ_transcript6257      1 MFSVKLAFAVIAAVYAAASAFPANETLEVGASCTITSFGQVSSVLNSCSNIVISGLSVPGGKTLELKLKSGSTVTFQGKTVFGVAHWDGPLLEIIGSKVTVKGASGHVLDGQGPKYWDGKGDGG 124
Pco_HQ_transcript10768     1 MFSVKLAFAVIAAVYAAASAFPANETLEVGASCTITSFGQVSSVLNSCSNIVISGLSVPGGKTLELKLKSGSTVTFQGKTVFGVAHWDGPLLEIIGSKVTVKGASGHVLDGQGPKYWDGKGDGG 124
Pco_HQ_transcript10701     1 MFSVKLAFAVIAAVYAAASAFPANETLEVGASCTITSFGQVSSVLNSCSNIVISGLSVPGGKTLELKLKSGSTVTFQGKTVFGVAHWDGPLLEIIGSKVTVKGASGHVLDGQGPKYWDGKGDGG 124
Pco_HQ_transcript282       1 MFSVKLAFAVIAAVYAAASAFPANETLEVGASCTITSFGQVSSVLNSCSNIVISGLSVPGGKTLELKLKSGSTVTFQGKTVFGVAHWDGPLLEIIGSKVTVKGASGHVLDGQGPKYWDGKGDGG 124
Pco_HQ_transcript12518     1 MFSVKLAFAVIAAVYAAASAFPANETLEVGASCTITSFGQVSSVLNSCSNIVISGLSVPGGKTLELKLKSGSTVTFQGKTVFGVAHWDGPLLEIIGSKVTVKGASGHVLDGQGPKYWDGKGDGG 124
PCO_GH28-10_contig_23457   1 MFSVKLAFAVIAAVYAAASAFPANETLEVGASCTITSFGQVSSVLNSCSNIVISGLSVPGGKTLELKLKSGSTVTFQGKTVFGVAHWDGPLLEIIGSKVTVKGASGHVLDGQGPKYWDGKGDNG 124
Pco_HQ_transcript11614     1 MFSVKLAFAVIAAVYAAASAFPANETLEVGASCTITSFGQVSSVLNSCSNIVISGLSVPGGKTLELKLKSGSTVTFQGKTVFGVAHWDGPLLEIIGSKVTVKGASGHVLDGQGPKYWDGKGDNG 124

PCO_GH28-9                   VKKPKFVKVRATGGSVLTGLHVLNCPRQCVSVNSCDHVTIDGWNVDVSAGDKNKLGHNTDGFDISSTSNVVISNTVVKNQDDCAVNKAQHVTISKLQCSGGHGLSLSVGMSKTSKADNTVSDV 248
PCO_GH28-9_contig_23458      VKKPKFVKVRATGGSVLTGLHVLNCPRQCVSVNSCDHVTIDGWNVDVSAGDKNKLGHNTDGFDISSTSNVVISNTVVKNQDDCAVNKAQHVTISKLQCSGGHGLSLSVGMSKSSKADNTVSDV 248
PCO_GH28-9_contig_15494      VKKPKFVKVRATGGSVLTGLHVLNCPRQCVSVNSCDHVTIDGWNVDVSAGDKNKLGHNTDGFDISSTSNVVISNTVVKNQDDCAVNKAQHVTISKLQCSGGHGLSLSVGMSKSSKADNTVSDV 248
PCO_GH28-9_contig_23457      VKKPKFVKVRATGGSVLTGLHVLNCPRQCVSVNSCDHVTIDGWNVDVSAGDKNKLGHNTDGFDISSTSNVVISNTVVKNQDDCAVNKAQHVTISKLQCSGGHGLSLSVGMSKSSKADNTVSDV 248
Pco_HQ_transcript12018       VKKPKFVKVRATGGSVLTGLHVLNCPRQCVSVNSCDHVTIDGWNVDVSAGDKNKLGHNTDGFDISSTSNVVISNTVVKNQDDCAVNKAQHVTISKLQCSGGHGLSLSVGMSKSSKADNTVSDV 248
Pco_HQ_transcript7296        VKKPKFVKVRATGGSVLTGLHVLNCPRQCVSVNSCDHVTIDGWNVDVSAGDKNKLGHNTDGFDISSTSNVVISNTVVKNQDDCAVNKAQHVTISKLQCSGGHGLSLSVGMSKSSKADNTVSDV 248
Pco_HQ_transcript6257        VKKPKFVKVRATGGSVLTGLHVLNCPRQCVSVNSCDHVTIDGWNVDVSAGDKNKLGHNTDGFDISSTSNVVISNTVVKNQDDCAVNKAQHVTISKLQCSGGHGLSLSVGMSKSSKADNTVSDV 248
Pco_HQ_transcript10768       VKKPKFVKVRATGGSVLTGLHVLNCPRQCVSVNSCDHVTIDGWNVDVSAGDKNKLGHNTDGFDISSTSNVVISNTVVKNQDDCAVNKAQHVTISKLQCSGGHGLSLSVGMSKSSKADNTVSDV 248
Pco_HQ_transcript10701       VKKPKFVKVRATGGSVLTGLHVLNCPRQCVSVNSCDHVTIDGWNVDVSAGDKNKLGHNTDGFDISSTSNVVISNTVVKNQDDCAVNKAQHVTISKLQCSGGHGLSLSVGMSKSSKADNTVSDV 248
Pco_HQ_transcript282         VKKPKFVKVRATGGSVLTGLHVLNCPRQCVSVNSCDHVTIDGWNVDVSAGDKNKLGHNTDGFDISSTSNVVISNTVVKNQDDCAVNKAQHVTISKLQCSGGHGLSLSVGMSKSSKADNTVSDV 248
Pco_HQ_transcript12518       VKKPKFVKVRATGGSVLTGLHVLNCPRQCVSVNSCDHVTIDGWNVDVSAGDKNKLGHNTDGFDISSTSNVVISNTVVKNQDDCAVNKAQHVTISKLQCSGGHGLSLSVGMSKSSKADNTVSDV 248
PCO_GH28-10_contig_23457     VKKPKFVKVRATGGSVLTGLHVLNCPRQCVSVDTCDHVTIDGWNVDVSAGDKNELGRNTDGFDISSTSNVVISNSVVKNQDDCAVNKAQHVTISKLQCSGGHGLSLSVGMSKTSKADNTVSDV 248
Pco_HQ_transcript11614       VKKPKFVKVRATGGSVLTGLHVLNCPRQCVSVDTCDHVTIDGWNVDVSAGDKNELGRNTDGFDISSTSNVVISNSVVKNQDDCAVNKAQHVTISKLQCSGGHGLSLSVGMSKTSKADNTVSDV 248

PCO_GH28-9                   HFSDCTVSNSNDNGIHIKTHSDGGPGVIDNVSYKNIKLSGIAKYGVNIEQDYEKGHPTGTPKANIPITGLSITGVSGSMTGKNSKAVYILCAKGGCSNWSWGVNISGAHKQSNCTMQPSNVKC*  371
PCO_GH28-9_contig_23458      HFSDCTVSNSNDNGIHIKTHSDGGPGVIDSVSYKNIKLSGIAKYGVNIEQDYEKGHPTGTPKANIPITGLSITGVSGSMTGKNSKAVYILCAKGGCSNWSWGVNISGAHKQNNCTMQPSNVKC*  371
PCO_GH28-9_contig_15494      HFSDCTVSNSNDNGIHIKTHSDGGPGVIDNVSYKNIKLSGIAKYGVNIEQDYEKGHPTGTPKANIPITGLSITGVSGSMTGKNSKAVYILCAKGGCSNWSWGVNISGAHKQSNCTMQPSNVKC*  371
PCO_GH28-9_contig_23457      HFSDCTVSNSNDNGIHIKTHSDGGPGVIDNVSYKNIKLSGIAKYGVNIEQDYEKGHPTGTPKANIPITGLSITGVSGSMTGKNSKAVYILCAKGGCSNWSWGVNISGAHKQSNCTMQPSNVKC*  371
Pco_HQ_transcript12018       HFSDCTVSNSNDNGIHIKTHSDGGPGVIDNVSYKNIKLSGIAKYGVNIEQDYEKGHPTGTPKANIPITGLSITGVSGSMTGKNSKAVYILCAKGGCSNWSWGVNISGAHKQSNCTMQPSNVKC*  371
Pco_HQ_transcript7296        HFSDCTVSNSNDNGIHIKTHSDGGPGVIDNVSYKNIKLSGIAKYGVNIEQDYEKGHPTGTPKANIPITGLSITGVSGSMTGKNSKAVYILCAKGGCSNWSWGVNISGAHKQSNCTMQPSNVKC*  371
Pco_HQ_transcript6257        HFSDCTVSNSNDNGIHIKTHSDGGPGVIDNVSYKNIKLSGIAKYGVNIEQDYEKGHPTGTPKANIPITGLSITGVSGSMTGKNSKAVYILCAKGGCSNWSWGVNISGAHKQSNCTMQPSNVKC*  371
Pco_HQ_transcript10768       HFSDCTVSNSNDNGIHIKTHSDGGPGVIDNVSYKNIKLSGIAKYGVNIEQDYEKGHPTGTPKANIPITGLSITGVSGSMTGKNSKAVYILCAKGGCSNWSWGVNISGAHKQSNCTMQPSNVKC*  371
Pco_HQ_transcript10701       HFSDCTVSNSNDNGIHIKTHSDGGPGVIDNVSYKNIKLSGIAKYGVNIEQDYEKGHPTGTPKANIPITGLSITGVSGSMTGKNSKAVYILCAKGGCSNWSWGVNISGAHKQSNCTMQPSNVKC*  371
Pco_HQ_transcript282         HFSDCTVSNSNDNGIHIKTHSDGGPGVIDNVSYKNIKLSGIAKYGVNIEQDYEKGHPTGTPKANIPITGLSITGVSGSMTGKNSKAVYILCAKGGCSNWSWGVNISGAHKQSNCTMQPSNVKC*  371
Pco_HQ_transcript12518       HFSDCTVSNSNDNGIHIKTHSDGGPGVIDNVSYKNIKLSGIAKYGVNIEQDYEKGHPTGTPKANIPITGLSITGVSGSMTGKNSKAVYILCAKGGCSNWSWSVNISGAHKQSNCTMQPSNVKC*  371
PCO_GH28-10_contig_23457     HFSDCTVSNSNDNGIHIKTHSDGGPGVIDNVSYKNIKLSGIRKYGVNIEQDYEKGSPTGTPKANIPITGLSITGVSGSMTGKNSRAVFILCAKGGCSNWSWGVNISGAYRQSNCTMQPSNVNC*  371
Pco_HQ_transcript11614       HFSDCTVSNSNDNGIHIKTHSDGGPGVIDNVSYKNIKLSGIRKYGVNIEQDYEKGSPTGTPKANIPITGLSITGVSGSMTGKNSRAVFILCAKGGCSNWSWGVNISGAYRQSNCTMQPSNVNC*  371
```

D

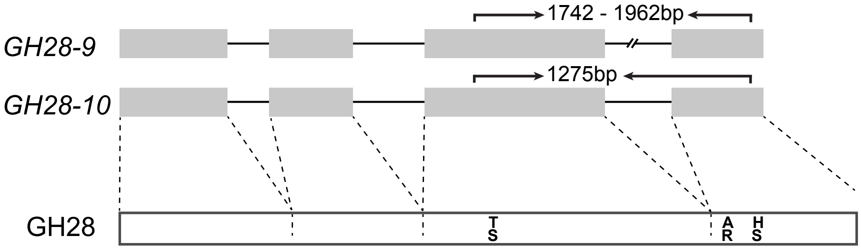
1742 - 1962bp
GH28-9
1275bp
GH28-10
GH28
T
S
A
R
H
S

E

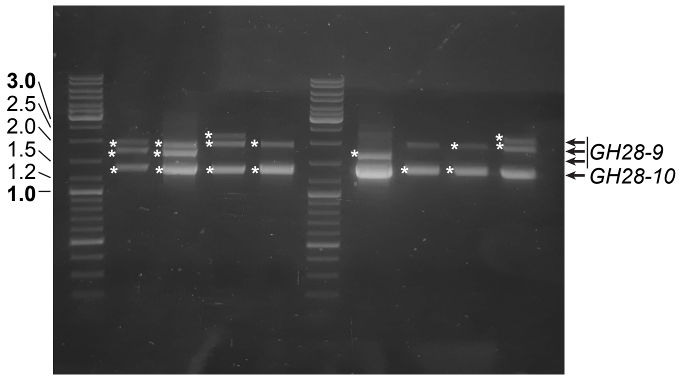
3.0
2.5
2.0
1.5
1.2
1.0
GH28-9
GH28-10

F

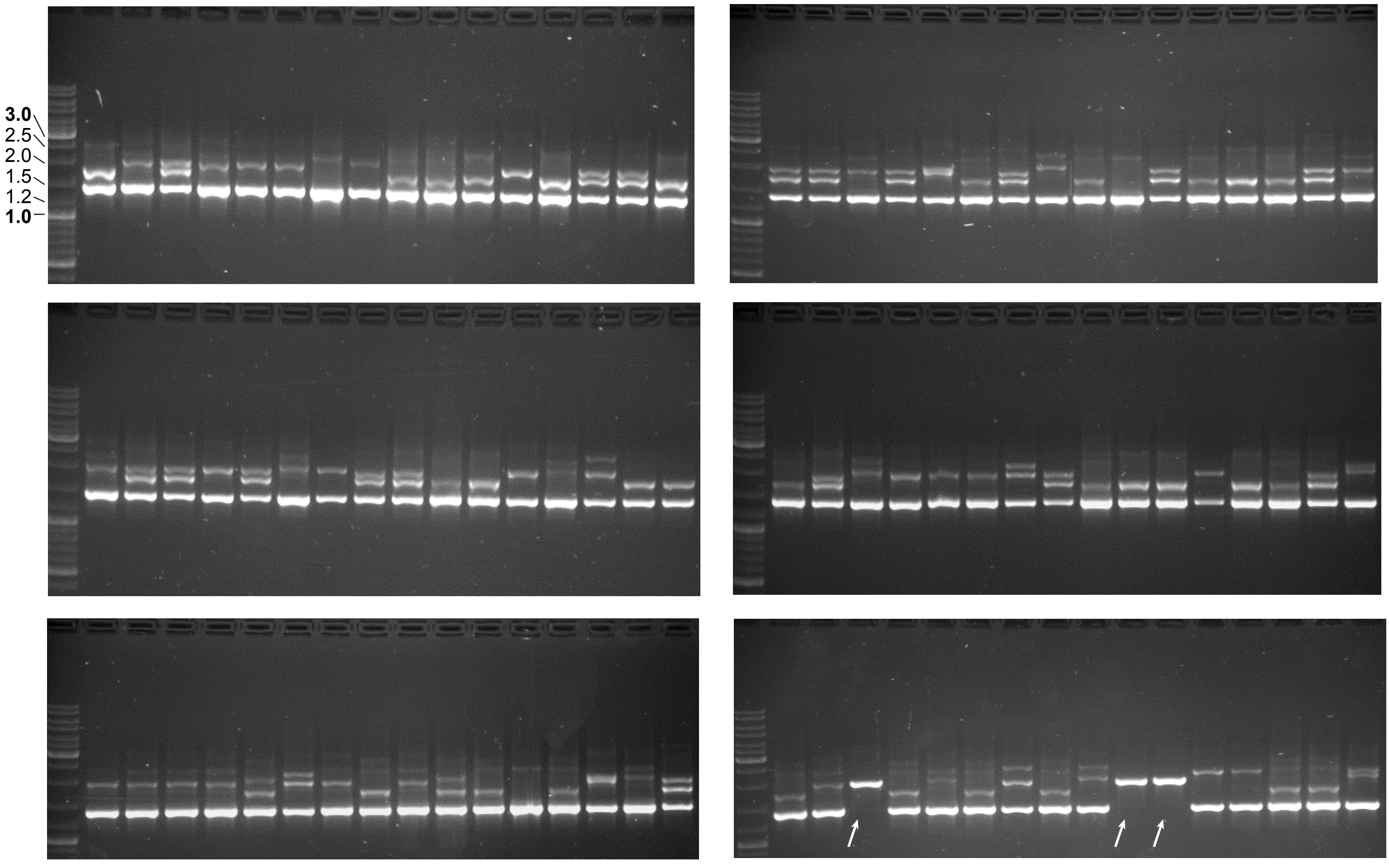
3.0
2.5
2.0
1.5
1.2
1.0

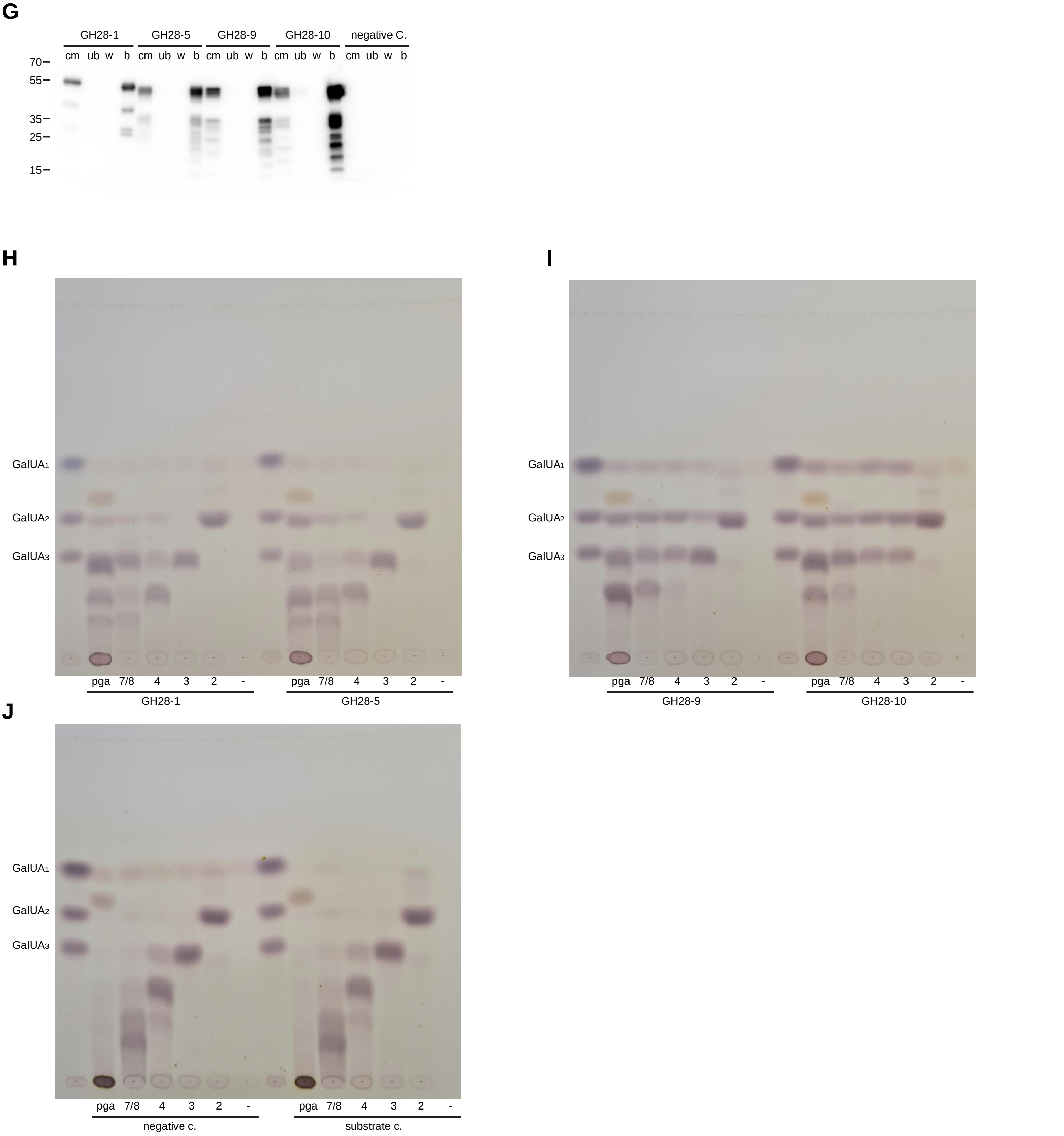

Figure S3 *GH28* copy number variation, haplotype frequency and functional characterization of the recent *GH28-10* copy in our *P. cochleariae* wild-type population. (A) Genome assembly generated from genomic DNA pooled from three individuals revealed three contigs with a *GH28-9*. Unlike in contig_23458 and contig_15494, in contig_23457 not only *GH28-9* but also an additional *GH28* (*GH28-10*) was found. Flanking genes were shown to be identical and their names refer to homologs in *Leptinotarsa decemlineata*. Bars above each gene represent 1000 nt. (B) Mapping the coding sequences of both *GH28* genes and flanking genes back to the raw reads confirmed *GH28* copy number variation. Asterisks indicate raw reads with *GH28-9* and *GH28-10*. (C) Multiple amino acid alignment of GH28-9 and GH28-10 sequences derived from RNA ISO-SEQ of RNA pooled from 10 individuals. Amino acid residues highlighted in blue are discriminating between the two *GH28* genes but those highlighted in grey are not. This confirms both genes to be transcribed in the beetle. PCO_GH28-9 corresponds to the published sequence (CCJ09449.1). (D) The exon-intron structure of the PG encoding genes *GH28-9* and *GH28-10* as well as a corresponding primary structure of a consensus GH28 protein are shown schematically. Binding sites of PCR primers to discriminate both genes based on length variability of the third intron are indicated with arrows and the estimated length of PCR products based on the genome assembly are written. Discriminative amino acids are highlighted below.
(E) Agarose gel-based separation of PCR products using genomic DNA from eight wild-type individuals and primers indicated in panel D. Bands indicated by asterisks were characterized by sequencing and their asignment to *GH28-9* and *GH28-10* respectively is shown next to the gel. (F) Large-scale screening of wild-type population revealed only three out of 96 individuals to lack *GH28-10* indicated by the absence of the corresponding band (arrows).
(G) Western blot verifying the heterologous expression and anti-V5 bead-based purification of the endo-PGs GH28-1, 5, 9 including the uncharacterized GH28-10. Culture medium of the Sf9 cells (cm), bead-unbound fraction (ub), bead-wash fraction (w) and bead-bound fraction (b) were analyzed. A negative control of non-transfected Sf9 cells was included. (H-J) Heterologously expressed proteins were tested for their pectolytic activity towards different substrates including the polymer polygalacturonic acid (pga) and oligomers ranging from octamer to dimer. "-" corresponds to the enzyme-only control. Hydrolysis products were separated by thin-layer chromatography along with the monomer to trimer galacturonic acid standard ($GalUA_1$ to $GalUA_3$). (J) The negative control (from non-transfected Sf9 cells) as well as the substrate control (substrate-only) were included.

A

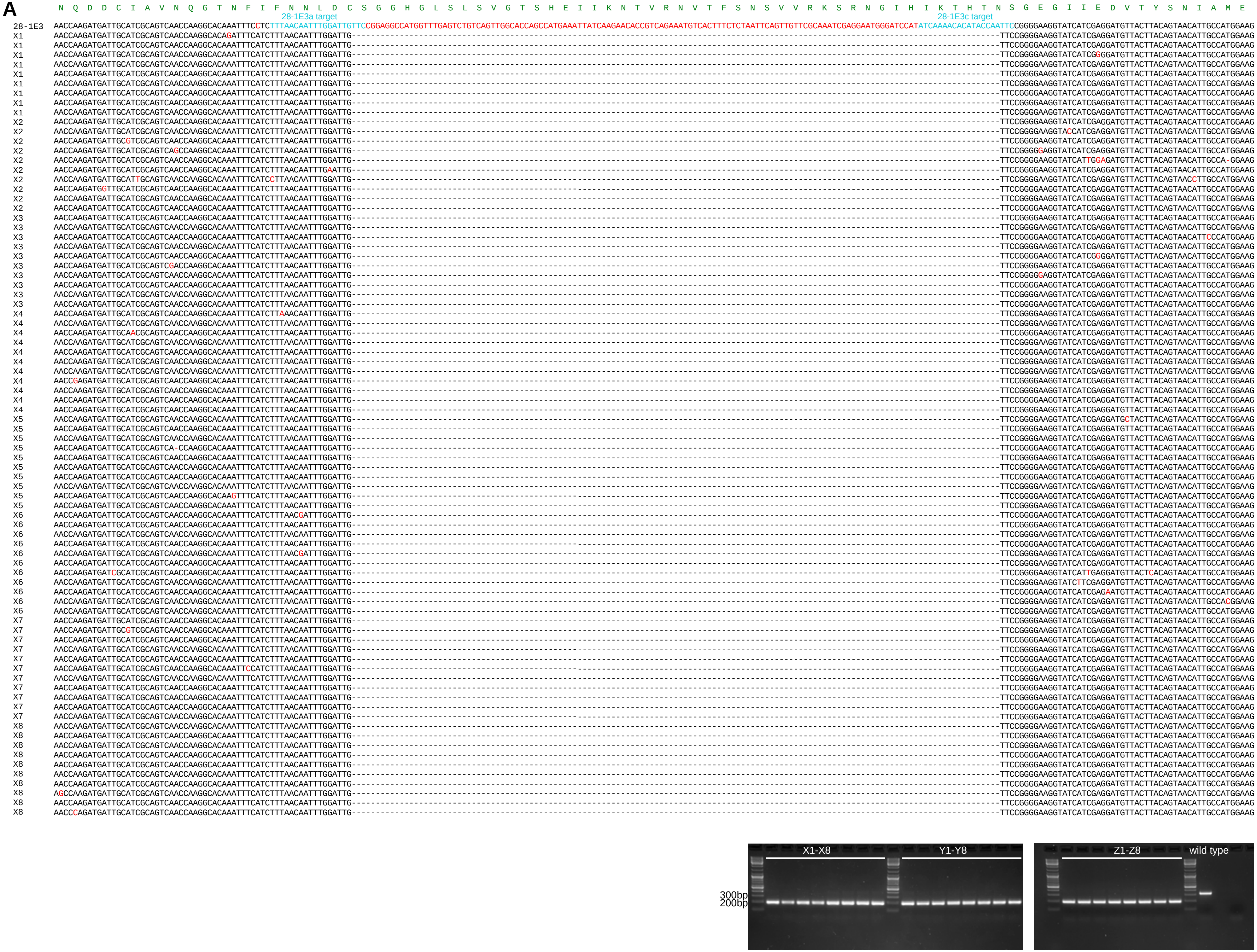
N Q D D C I A V N Q G T N F I F N N L D C S G G H G L S L S V G T S H E I I K N T V R N V T F S N S V V R K S R N G I H I K T H T N S G E G I I E D V T Y S N I A M E
28-1E3a target
28-1E3c target
28-1E3
X1
X2
X3
X4
X5
X6
X7
X8
X1-X8
Y1-Y8
Z1-Z8
wild type
300bp
200bp

B

```
         N Q D D C V A V N K A H Y V T L S K L Q C S G G H G L S L S V G M S K T S K E D N T V S D V H F
                                                              28-5/9E3a target                28-5/9E3b target
28-5E3  AACCAGGACGATTGCGTGGCTGTCAACAAGGCCCACTACGTGACCCTCTCCAAGCTGCAGTGCTCAGGCGGCCACGGACTCAGTCTGTCTGTGGGCATGAGCAAAACGTCCAAGGAAGATAACACCGTGTCCGATGTCCACTTCT
X1      AACCAGGACGATTGCGTGGCTGTCAACAAGGCC-----------------------------------------CGGACTCAGTCTGTCTGTGGGCATGAG---------CAAGGAAGATAACACCGTGTCCGATGTCCACTTCT
X1      AACCAGGACGATTGCGTGGCTGTCAACAAGGCC-----------------------------------------CGGACTCAGTCTGTCTGTGGGCATGAG---------CAAGGAAGATAACACCGTGTCCGATGTCCACTTCT
X2      AACCAGGACGATTGCGTGGCTGTCAACAAGGCC-----------------------------------------CGGACTCAGTCTGTCTGTGGGCATGAG---------CAAGGAAGATAACACCGTGTCCGATGTCCACTTCT
X3      AACCAGGACGATTGCGTGGCTGTCAACAAGGCC-----------------------------------------CGGACTCAGTCTGTCTGTGGGCATGAG---------CAAGGAAGATAACACCGTGTCCGATGTTCACTTCT
X3      AACCAGGACGATTGCGTGGCTGTCAACAAGGCC-----------------------------------------CGGACTCAGTCTGTCTGTGGGCATGAG---------CAAGGAAGATAACACCGTGTCCGATGTCCACTTCT
X3      AACCAGGACGATTGCGTGGCTGTCAACAAGGCC-----------------------------------------CGGACTCAGTCTGTCTGTGGGCATGAG---------CAAGGAAGATAACACCGTGTCCGATGTCCACTTCT
X3      AACCAGGACGATTGCGTGGCTGTCAACAAGGCC-----------------------------------------CGGACTCAGTCTGTCTGTGGGCATGAG---------CAAGGAAGATAACACCGTGTCCGATGTCCACTTCT
X3      AACCAGGACGATTGCGTGGCTGTCAACAAGGCC-----------------------------------------CGGACTCAGTCTGTCTGTGGGCATGAG---------CAAGGAAGATAACACCGTGTCCGATGTCCACTTCT
X4      GACCAGGACGATTGCGTGGCTGTCAACAAGGCC-----------------------------------------CGGACTCAGTCTGTCTGTGGGCATGAG---------CAAGGAAGATAACACCGTGTCCGATGTCCACTTCT
X4      AACCAGGACGATTGCGTGGCTGTCAACAAGGCC-----------------------------------------CGGACTCAGTCTGTCTGTGGGCATGAG---------CAAGGAAGATAACACCGTGTCCGATGTCCACTTCT
X5      AACCAGGACGATTGCGTGGCTGTCAACAAGGCC-----------------------------------------CGGACTCAGTCTGTCTGTGGGCATGAG---------CAAGGAAGATAACACCGTGTCCGATGTCCACTTCT
X5      AACCAGGACGATTGCGTGGCTGTCAACAAGGCC-----------------------------------------CGGACTCAGTCTGTCTGTGGGCATGAG---------CAAGGAAGATAACACCGTGTCCGATGTCCACTTCT
X5      AACCAGGACGATTGCGTGGCCGTCAACAAGGCC-----------------------------------------CGGACTCAGTCTGTCTGTGGGCATGAG---------CAAGGAAGATAACACCGTGTCCGATGTCCACTTCT
X5      AACCAGGACGATTGCGTGGCTGTCAACAAGGCC-----------------------------------------CGGACTCAGTCTGTCTGTGGGCATGAG---------CAAGGAAGATAACACCGTGTCCGATGTCCACTTCT
X6      AACCAGGACGATTGCGTGGGTGTCAACAAGGCC-----------------------------------------CGGACTCAGTCTGTCTGTGGGCATGAG---------CAAGGAAGATAACACCGTGTCCGATGTCCACTTCT
X6      AACCAGGACGATTGCGTGGCTGTCAACAAGGCC-----------------------------------------CGGACTCAGTCTGTCTGTGGGCATGAG---------CAAGGAAGATAACACCGTGTCCGATGTCCACTTCT
X7      AACCAGGACGATTGCGTGGCTGTCAACAAGGCC-----------------------------------------CGGACTCAGTCTGTCTGTGGGCATGAG---------CAAGGAAGATAACACCGCGTCCGATGTCCACTTCT
X7      AACCAGGACGATTGCGTGGCTGTCAACAAGGCC-----------------------------------------CGGACTCAGTCTGTCTGTGGGCATGAG---------CAAGGAAGATAACACCGTGTCCGATGTCCACTTCT
X7      AACCAGGACGATTGCGTGGCTGTCAACAAGGCC-----------------------------------------CGGACTCAGTCTGTCTGTGGGCATGAG---------CAAGGAAGATAACACCGTGACCGATGTCCACTTCT
X8      AACCAGGACGATTGCGTGGCTGTCAACAAGGCC-----------------------------------------CGGACTCAGTCTGTCTGTGGGCATGAG---------CAAGGAAGACAACACCGTGTCCGATGTCCACTTCT
Y1      AACCAGGACGATTGCGTGGCTGTCAACAAGGCC-----------------------------------------CGGACTCAGTCTGTCTGTGGGCATGAG---------CAAGGAAGATAACACCGTGTCCGATGTCCACTTCT
Y1      AACCAGGACGATTGCGTGGCTGTCAACAAGGCC-----------------------------------------CGGACTCAGTCTGTCTGTGGGCATGAG---------CAAGGAAGATAACACCGTGTCCGATGTCCACTTCT
Y1      AACCAGGACGATTGCGTGGCTGTCAACAAGGCC-----------------------------------------CGGACTCAGTCTGTCTGTGGGCATGAG---------CAAGGAAGATAACACCGTGTCCGATGTCCACTTCT
Y1      AACCAGGACGATTGCGTGGCTGTCAACAAGGCC-----------------------------------------CGGACTCAGTCTGTCTGTGGGCATGAG---------CAAGGAAGATAACACCGTGTCCGATGTCCACTTCT
Y1      AACCAGGACGATTGCGTGGCTGTCAACAAGGCC-----------------------------------------CGGACTCAGTCTGTCTGTGGGCATGAG---------CAAGGAAGATAACACCGTGTCCGATGTCCACTTCT
Y2      AACCAGGACGATTGCGTGGCTGTCAACAAGGCC-----------------------------------------CGGACTCAGTCTGTCTGTGGGCATGAG---------CAAGGAAGATAACACCGTGTCCGATGTCCACTTCT
Y2      AACCAGGACGATTGCGTGGCTGTCAACAAGGCC-----------------------------------------CGGACTCAGTCTGTCTGTGGGCATGAG---------CAAGGAAGATAACACCGTGTCCGATGTCCACTTCT
Y2      AACCAGGACGATTGCGTGGCTGTCAACAAGGCC-----------------------------------------CGGACTCAGTCTGTCTGTGGGCATGAG---------CAAGGAAGATAACACCGTGTCCGATGTCCACTTCT
Y2      AACCAGGACGATTGCGTGGCTGTCAACAAGGCC-----------------------------------------CGGACTCAGTCTGTCTGTGGGCATGAG---------CAAGGAAGATAACACCGTGTCCGATGTCCACTTCT
Y2      AACCAGGACGATTGCGTGGCTGTCAACAAGGCC-----------------------------------------CGGACTCAGTCTGTCTGTGGGCATGAG---------CAAGGAAGATAATACCGTGTCCGATGTCCACTTCT
Y3      AACCAGGACGATTGCGTGGCTGTCAACAAGGCC-----------------------------------------CGGACTCAGTCCGTCTGTGGGCATGAG---------CAAGGAAGATAACACCGTGTCCGATGTCCACTTCT
Y3      AACCAGGACGATTGCGTGGCTGTCAACAAGGCC-----------------------------------------CGGACTCAGTCTGTCTGTGGGCATGAG---------CAAGGAAGATAACACCGTGTCCGATGTCCACTTCT
Y3      AACCAGGACGATTGCGTGGCTGTCAACAAGGCC-----------------------------------------CGGACTCAGTCTGTCTGTGGGCATGAG---------CAAGGAAGATAACACCGTGTCCGATGTCCACTACT
Y3      AACCAGGACGATTGCGTGGCTGTCAACAAGGCC-----------------------------------------CGGACTCAGTCTGTCTGTGGGCATGAG---------CAAGGAAGATAACACCGTGTCCGATGTCCACTTCT
Y4      AACCAGGACGATTGCGTGGCTGTCAACAAGGCC-----------------------------------------CGGACTCAGTCTGTCTGTGGGCATGAG---------CAAGGAAGATAACACCGTGTCCGATGTCCACTTCT
Y4      AACCAGGACGATTGCGTGGCTGTCAACAAGGCC-----------------------------------------CGGACTCAGTCTGTCTGTGGGCATGAG---------CAAGGAAGATAACACCGTGTCCGATGTCCACTTCT
Y4      AACCAGGA-GATTGCGTGGCTGTCAACAAGGCC-----------------------------------------CGGACTCAGTCTGTCTGTGGGCATGAG---------CAAGGAAGATAACACCGTGTCCGATGTCCACTTCT
Y4      AACCAGGACGATTGCGTGGCTGTCAACAAGGCC-----------------------------------------CGGACTCAGTCTGTCTGTGGGCATGAG---------CAAGGAAGATAACACCGTGTCCGATGTCCACTTCT
Y4      AACCAGGACGATTGTGTGGCTGTCGACAAGGCC-----------------------------------------CGGACTCCGTCTGTCTGTGGGCATGAG---------CAAGGAAGATAACACCGTGTCCGATGTCCACTTCT
Y4      AACCAGGACGATTGCGTGGCTGTCAACAAGGCC-----------------------------------------CGGACTCAGTCTGTCTGTGGGCATGAG---------CAAGGAAGATAACACCGTGTCCGATGTCCACTTCT
Y5      AACCAGGACGATTGCGTGGCTGTCAACAAGGCC-----------------------------------------CGGACTCAGTCTGTCTGTGGGCATGAG---------CAAGGAAGATAACACCGTGTCCGATGTCCACTTCT
Y5      AACCAGGACGATTGCGTGGCTGTCAACAAGGCC-----------------------------------------CGGACTCAGTCTGTCTGTGGGCATGAG---------CAAGGAAGATAACACCGTGTCCGATGTCCACTTCT
Y5      AACCAGGACGATTGCGTGGCTGTCAACAGGGCC-----------------------------------------CGGACTCAGTCTGTCTGTGGGCACGAG---------CAAGGAAGATAACACCGTGTCCGATGTCCACTTCT
Y5      AACCAGGACGATTGCGTGGCTGTCAACAAGGCC-----------------------------------------CGGACTCAGTCTGTCTGTGGGCATGAG---------CAAGGAAGATAACACCGTGTCCGATGTCCACTTCT
Y5      AACCAGGACGATTGCGTGGCTGTCAACAAGGCC-----------------------------------------CGGACTCAGTCTGTCTGTGGGCATGAG---------CAAGGAAGATAACACCGTGTCCGATGTCCACTTCT
Y6      AACCAGGACGATTGCGTGGCTGTCAACAAGGCC-----------------------------------------CGGACTCAGTCTGTCTGTGGGCATGAG---------CAAGGAAGATAACACCGTGTCCGATGTCCACTTCT
Y6      AACCAGGACGATCGCGTGGCTGTCAACAAGGCC-----------------------------------------CGGACTCAGTCTGTCTGTGGGCATGAG---------CAAGGAAGATAACACCGTGTCCGATGTCCACTTCT
Y6      AACCAGGACGATTGCGCGGCTGTCAACAAGGCC-----------------------------------------CGGACTCAGCCTGTCTGTGGGCATGAG---------CAAGGAAGATAACACCGTGTCCGATGTCCACTTCT
Y6      AACCAGGACGATTGCGTGGCTGTCAACAAGGCC-----------------------------------------CGGACTCAGTCTGTCTGTGGGCATGAG---------CAAGGAAGATAACACCGTGTCCGATGTCCACTTCT
Y6      AACCAGGACGATTGCGTGGCTGTCAACAAGGCC-----------------------------------------CGGACTCAGTCTGTCTGTGGGCATGAG---------CAAGGAAGATAACACCGTGTCCGATGTCCACTTCT
Y6      AACCAGGACGATTGCGTGGCTGTCAACAAGGCC-----------------------------------------CGGACTCAGTCTGTCTGTGGGCATGAG---------CAAGGAAGATAACACCGTGTCCGATGTCCACTTCT
Y7      AACCAGGACGATTGCGTGGCTGTCAACAAGGCC-----------------------------------------CGGACTCAGTCTGTCTGTGGGCATGAG---------CAAGGAAGATAACACCGTGTCCGATGTCCACTTCT
Y7      AACCAGGACGATTGCGTGGCTGTCAACAAGGCC-----------------------------------------CGGACTCAGTCTGTCTGTGGGCATGAG---------CAAGGAAGATAACACCGTGTCCGATGTCCACTTCT
Y7      AACCAGGACGATTGCGTGGCTGTCAACAAGGCC-----------------------------------------CGGACTCAGTCTGTCTGTGGGCATGAG---------CAAGGAAGATAACACCGTGTCCGTTGTCCACTTCT
Y7      AACCAGGACGATTGCGTGGCTGTCAACAAGGCC-----------------------------------------CGGACTCAGTCTGTCTGTGGGCATGAG---------CAAGGAAGATAACACCGTGTCCGATGTCCACTTCT
Y7      AACCAGGACGATTGCGTGGCTGTCAACAAGGCC-----------------------------------------CGGACTCAGTCTGTCTGTGGGCATGAG---------CAAGGAAGATAACACCGTGTCCGATGTCCACTTCT
Y7      AACCAGGACGATTGCGTGGCTGTCAACAAGGCC-----------------------------------------CGGACTCAGTCTGTCTGTGGGCATGAG---------CAAGGAAGATAACACCGTGTCCGATGTCCACTTCT
Y7      AACCAGGACGATTGC--GGCTGTCAACAAGGCC-----------------------------------------CGGACTCAGTCTGTCTGTGGGCATGAG---------CAAGGAAGATAACACCGTGTCCGATGTCCACTTCT
Y8      AACCAGGACGATTGCGTGGCTGTCAACAAGGCC-----------------------------------------CGGACTCAGTCTGTCTGTGGGCATGAG---------CAAGGAAGATAACACCGTGTCCGATGTCCACTTCT
Y8      AACCAGGACGATTGCGTGGCTGTCAACAAGGCC-----------------------------------------CGGACTCAGTCTGTCTGTGGGCATGAG---------CAAGGAAGATAACACCGTGTCCGATGTCCACTTCT
Y8      AACCAGGACGATTGCGTGGCTGTCAACAAGGCC-----------------------------------------CGGACTCAGTCTGTCTGTGGGCATGAG---------CAAGGAAGATAACACCGTGTCCGATGTCCACTTCT
Z1      AACCAGGACGATTGCGTGGCTGTCAACGTGGCC-----------------------------------------CGGACTCAGTCTGTCTGTGGGCATGAG---------CATGGAAGATAACACCGTGTCCGATGTCCACTTCT
Z1      AACCAGGACGATTGCGTGGCTGTCAACAAGGCC-----------------------------------------CGGACTCAGTCTGTCTGTGGGCATGAG---------CAAGGAAGATAACACCGTGTCCGATGTCCACTTCT
Z1      AACCAGGACGATTGCGTGGCTGTCAACAAGGCC-----------------------------------------CGGACTCAGTCTGTCTGTGGGCATGAG---------CAAGGAAGATAACACCGTGTCCGATGTCCACTTCT
Z1      AACCAGGACGATTGCGTGGCTGTCAACAAGGCC-----------------------------------------CGGACTCAGTCTGTCTGTGGGCATGAG---------CAAGGAAGATAACACCGTGTCCGATGTCCACTTCT
Z1      AACCAGGACGATTGCGTGGCTGTCAACAAGGCC-----------------------------------------CGGACTCAGTCTGTCTGTGGGCATGAG---------CAAGGAAGATAACACCGTGTCCGATGTCCACTTCT
Z2      AACCAGGACGATTGCGTGGCTGTCAACAAAGCC-----------------------------------------CGGACTCAGTCTGTCTGTGGGCATGAG---------CAAGGAAGATAACACCGTGTCCGATGTCCACTTCT
Z2      AACCAGGACGATTGCGTGGCTGTCAACAAGGCC-----------------------------------------CGGACTCAGTCTGTCTGTGGGCATGAG---------CAAGGAAGATAACACCGTGTCCGATGTCCACTTCT
Z2      AACCAGGACGATTGCGTGGCTGTCAACAAGGCC-----------------------------------------CGGACTCAGTCTGTCTGTGGGCATGAG---------CAAGGAAGATAACACCGTGTCCGATGTCCACTTCT
Z2      AACCAGGACGATTGCGTGGCTGTCAACAAGGCC-----------------------------------------CGGACTCAGTCTGTCTGTGGGCATGAG---------CAAGGAAGATAACACCGTGTCCGATGTCCACTTCT
Z2      AACCAGGACGATTGCGTGGCTGTCAACAAGGCC-----------------------------------------CGGACTCAGTCTGTCTGTGGGCATGAG---------CAAGGAAGATAACACCGTGTCCGATGTCCACTTCT
Z3      AACCAGGGCGATTGCGTGGCTGTCAACAAGGCC-----------------------------------------CGGACTCAGTCTGTCTGTGGGCATGAG---------CAAGGAAGATAACACCGTGTCCGATGTCCACTTCT
Z3      AACCAGGACGATTGCGTGGCTGTCAACAAGGCC-----------------------------------------CGGACTCAGTCTGTCTGTGGGCATGAG---------CAAGGGAGATAACACCGTGTCCGATGTCCACTTCT
Z3      AACCAGGACGATTGCGTGGCTGTCAACAAGGCC-----------------------------------------CGGACTCAGTCTGTCTGTGGGCATGAG---------CAAGGAAGATAACACCGTGTCCGATGTCCACTTCT
Z3      AACCAGGACGATTGCGTGGCTGTCAACAAGGAC-----------------------------------------CGGACTCAGTCTGTCTGTGGGCATGAG---------CAAGGAAGATAACACCGTGTCCGATGTCCACTTCT
Z3      AACCAGGACGATTGCGTGGCTGTCAACAAGGCC-----------------------------------------CGGACTCAGTCTGTCTGTGGGCATGAG---------CAAGGAAGATAACACCGTGTCCGATGTCCACTTCT
Z4      AACCAGGACGATTGCGTGGCCGTCAACAAGGCC-----------------------------------------CGGACTCAGTCTGTCTGTGGGCATGAG---------CAAGGAAGATAACACCGTGTCCGATGTCCACTTCT
Z4      AACCAGGACGATTGCGTGGCTGTCAACAAGGCC-----------------------------------------CGGACTCAGTCTGTCCGTGGGCATGAG---------CAAGGAAGATAACACCGTGTCCGATGTCCACTTCT
Z4      AACCAGCACGATTGCGTGGCGGTCAACAACAAC-----------------------------------------CGCAGTCAGTCGTTCTGTGGGCATCAG---------CAACAAAGAAAACACCGCGTCCGATGTCCACCTCT
Z5      AACCAGGACGATTGCGTGGCTGTCAACAAGGCC-----------------------------------------CGGACTCAGTCTGTCTGTGGGCATGAG---------CAAGGAAGATAACACCGTGTCCGATGTCCACTTCT
Z5      AACCAGGACGATTGCGTGGCTGTCAACAAGGCC-----------------------------------------CGGACTCAGTCTGTCTGTGGGCATGAG---------CAAGGAAGATAACACCGTGTCCGATGTCCACTTCT
Z5      AACCAGGACAATTGCGTGGCTGTCAACAAGGCC-----------------------------------------CGGACTCAGTCTGTCTGTGGGCATGAG---------CAAGGAAGATAACACCGTGTCCGATGTCCACTTCT
Z5      AACCAGGACGATTGCGTGGCTGTCAACAAGGCC-----------------------------------------CGGACTCAGTCTGTCTGTGGGCATGAG---------CAAGGAAGATAACACCGTGTCCGATGTCCACTTCT
Z5      AACCAGGACGATTGCGTGGCTGTCAACAAGGCC-----------------------------------------CGGACTCAGTCTGTCTGTGGGCATGAG---------CAAGGAAGATAACACCGTGTCCGATGTCCACTTCT
Z5      AACCAGGACGATTGCGTGGCTGTCAACAGGGCC-----------------------------------------CGGACTCCGTCTGTCTGTGGGCATGAG---------CAAGGAAGATAACACCGTGTCCGATGTCCACTTCT
Z5      AACCAGGACGATTGCGTGGCTGTCAACAAGGCC-----------------------------------------CGGACTCAGTCTGTCTGTGGGCATGAG---------CAAGGAAGATAACACCGTGTCCGATGTCCACTTCT
Z6      AACCAGGACGATTGCGTGGCTGTCAACAAGGCC-----------------------------------------CGGACTCAGTCTGTCTGTGGGCATGAG---------CAAGGAAGATAACACCGTGTCCGATGTCCACTTCT
Z6      AACCAGGACGATTGCGTGGCTGTCAACAAGGCC-----------------------------------------CGGACTCAGTCTGTCTGTGGGCATGAG---------CAAGGAAGATAACACCGTGTCCGATGTCCACTTCT
Z6      AACCAGGACGATTGCGTGGCTGTCAACAAGGCC-----------------------------------------CGGACTCAGTCTGTCTGTGGGCATGAG---------CGAGGAAGATAACACCGTGTCCGATGTCCACTTCT
Z6      AACCAGGACGATTGCGTGGCTGTCAACAAGGAC-----------------------------------------CGGACTCAGTCTGTCTGTGGGCATGAG---------CAAGGAAGATAACACCGTGTCCGATGTCCACTTCT
Z6      AACCAGGACGATTGCGTGGCTGTCAACAAGGCC-----------------------------------------CGGACTCAGTCTGTCTGTGGGCATGAG---------CAAGGAAGATAACACCGTGTCCGATGTCCACTTCT
Z6      AACCAGGACGATTGCGTGGCTGTCAACAAGGCC-----------------------------------------CGGACTCAGTCTGTCTGTGGGCATGAG---------CAAGGAAGATAACACCGTGTCCGATGTCCACTTCT
Z7      AACCAGGACGATTGCGTGGCTGTCAACAAGGCC-----------------------------------------CGGACTCAGTCTGTCTGTGGGCATGAG---------CAAGGAAGATAACACCGTGTCCGATGTCCACTTCT
Z7      AACCAGGACGATTGCGTGGCTGTCAACAAGGCC-----------------------------------------CGGACTCAGTCTGTCTGTGGGCATGAG---------CAAGGAAGATAACACCGTGTCCGGTGTCCACTTCT
Z7      AACCAGGACGATTGCGTGGCTGTCAACAAGGCC-----------------------------------------CGGACTCAGTCTGTCTGTGGGCATGAG---------CAAGGAAGATAACACCGTGTCCGATGTCCACTTCT
Z7      AACCAGGACGATTGCGTGGCTGTCAACAAGGCC-----------------------------------------CGGACTCAGTCTGTCTGT-GGCATGAG---------CAAGGAAGATAACACCGTGTCCGATGTCCACTTCT
Z7      AACCAGGACGATTGCGTGGCTGTCAACAAGGCC-----------------------------------------CGGACTCAGTCTGTCTGTGGGCATGAG---------CAAGGAAGATAACACCGTGTCCGATGTCCACTTCT
Z7      AACCAGGACGATTGCGTGGCTGTCAACAAGGCC-----------------------------------------CGGACTCAGTCTGTCTGTGGGCATGAG---------CAAGGAAGATAACACCGTGTCCGATGTCCACTTCT
Z8      AACCAGGACGATTGCGTGGCTGTCAACAAGGCC-----------------------------------------CGGACTCAGTCTGTCTGTGGGCATGAG---------CAAGGAAGATGGCACCGTGTCCGATGTCCACTTCT
Z8      AACCAGGACGATTGCGTGGCTGTCAACGAGGCC-----------------------------------------CGGACTCAGTCTGTCCGTGGGCATGAG---------CAAGGAAGATAACACCGTGTCCGATGTCCACTTCT
Z8      AACCAGGACGATTGCGTGGCTGTCAACAAGGCC-----------------------------------------CGGACTCAGTCTGTCTGTGGGCATGAG---------CAAGGAAGATAACACCGTGTCCGATGTCCACTTCT
Z8      AACCAGGACGATTGCGTGGCTGTCAACAAGGCC-----------------------------------------CGGACTCAGTCTGTCTGTGGGCATGAG---------CAAGGAAGATAACACCGTGTCCGATGTCCACTTCT
Z8      AACCAGGACGATTGCGTGGCTGTCAACAAGGCC-----------------------------------------CGGGCTCAGTCTGTCTGTGGGCATGAG---------CAAGGAAGATAACACCGTGTCCGATGTCCACTTCT
Z8      AACCAGGACGATTGCGTGGCTGTCAACAAGGCC-----------------------------------------CGGACTCAGTCTGTCTGTGGGCATGAG---------CAAGGAAGATAACACCGTGTCCGATGTCCACTTCT
Z8      AACCAGGACGATTGCGTGGCTGTCAACAAGGCC-----------------------------------------CGGACTCAGTCTGTCTGTGGGCATGAG---------CAAGGAAGATAACACCGTGTCCGATGTCCACTTCT
Z8      AACCAGGACGATTGCGTGGCTGTCAACAAGGCC-----------------------------------------CGGACTCAGTCTGTCCGTGGGCATGAG---------CAAGGAAGATAACACCGTGTCCGATGTCCACTTCT
```

C

```
           L  A  F  A  V  I  A  A  V  Y  A  A  A  S  A  F  P  A  N  E  T  L  E  V  G  A  S  C  T  I  T  S  F  G  Q  V  S  S  V  L  N  S  C  S  N  I
                                                                                        28-5/9E1 target
28-9E1     CTAGCATTCGCGGTAATCGCAGCAGTCTACGCTGCAGCTTCGGCCTTCCCGGCCAACGAGACTCTGGAGGTGGGCGCCAGCTGCACCATCACCAGCTTCGGTCAGGTCAGCTCGGTGCTGAACAGCTGCTCCAACATCGT
X1  28-9   CTAGCATTCGCGGTAATCGCAGCAGTCTACGCTGCAGCTTCGGCCTTCCCGGCCAACGAGACTCTGGAGGTGGGCGCCAGCTGCACCA---------TCGGTCAGGTCAGCTCGGTGCTGAACAGCTGCTCCAACATCGT
X1         CTAGCATTCGCGGTAATCGCAGCAGTCTACGCTGCAGCTTCGGCCTTCCCGGCCAACGAGACTCTGGAGGTGGGCGCCAGCTGCACCA---------TCGGTCAGGTCAGCTCGGTGCTGAACAGCTGCTCCAACATCGT
X1         CTAGCATTCGCGGTAATCGCAGCAGTCTACGCTGCAGCTTCGGCCTTCCCGGCCAACGAGACTCTGGAGGTGGGCGCCAGCTGCACCA---------TCGGTCAGGTCAGCTCGGTGCTGAACAGCTGCTCCAACATCGT
X1         CTAGCATTCGCGGTAATCGCAGCAGTCTACGCTGCAGCTTCGGCCTTCCCGGCCAACGAGACTCTGGAGGTGGGCGCCAGCTGCACCA---------TCGGTCAGGTCAGCTCGGTGCTGAACAGCTGCTCCAACATCGT
X1         CTAGCATTCGCGGTAATCGCAGCAGTCTACGCTGCAGCTTCGGCCTTCCCGGCCAACGAGACTCTGGAGGTGGGCGCCAGCTGCACCA---------TCGGTCAGGTCAGCTCGGTGCTGAACAGCTGCTCCAACATCGT
X1  28-10  CTAGCATTCGCGGTAATCGCAGCAGTCTACGCTGCAGCTTCGGCCTTCCCGGCCAACGAGACTCTGGAGGTGGGCGCCAGCTGC-----------CTTCGGTCAGGTCAGCTCGGTGCTGAACAGCTGCTCCAACATCGT
X1         CTAGCATTCGCGGTGATCGCAGCAGTCTACGCTGCAGCTTCGGCCTTCCCGGCCAACGAGACTCTGGAGGTGGGCGCCAGCTGC-----------CTTCGGCCAGGTCAGCTCGGTGCTGAACAGCTGCTCCAACATCGT
X1         CTAGCATTCGCGGTGATCGCAGCAGTCTACGCTGCAGCTTCGGCCTTCCCGGCCAACGAGACTCTGGAGGTGGGCGCCAGCTGC-----------CTTCGGTCAGGTCAGCTCGGTGCTGAACAGCTGCTCCAACATCGT
X2         CTAGCATTCGCGGTAATCGCAGCAGTCTACGCTGCAGCTTCGGCCTTCCCGGCCAACGAGACTCTGGAGGTGGGCGCCAGCTGCACCA---------TCGGTCAGGTCAGCTCGGTGCTGAACAGCTGCTCCAACATCGT
X2         CTAGCATTCGCGGTAATCGCAGCAGTCTACGCTGCAGCTTCGGCCTTCCCGGCCAACGAGACTCTGGAGGTGGGCGCCAGCTGCACCA---------TCGGTCAGGTCAGCTCGGTGCTGAACAGCTGCTCCAACATCGT
X2         CTAGCATTCGCGGTAATCGCAGCAGTCTACGCTGCAGCTTCGGCCTTCCCGGCCAACGAGACTCTGGAGGTGGGCGCCAGCTGCACCA---------TCGGTCAGGTCAGCTCGGTGCTGAACAGCTGCTCCAACATCGT
X2         CTAGCATTCGCGGTAATCGCAGCGGTCTACGCTGCAGCTTCGGCCTTCCCGGCCAACGAGACTCTGGAGGTGGGCGCCAGCTGCACCA---------TCGGTCAGGTCAGCTCGGTGCTGAACAGCTGCTCCAACATCGT
X2         CTAGCATTCGCGGTAATCGCAGCAGTCTACGCTGCAGCTTCGGCCTTCCCGGCCAACGAGACTCTGGAGGTGGGCGCCAGCTGCACCA---------TCGGTCAGGTCAGCTCGGTGCTGAACAGCAGCTCCAACATCGT
X2         CTAGCATTCGTGGTGATCGCAGCAGTCTACGCTGCAGCTTCAGCCTTCCCGGCCAACGAGACTCTGGAGGTGGGCGCCAGCTGC-----------CTTCGGTCAGGTCAGCTCGGTGCTGAACAGCTGCTCCAACATCGT
X2         CTAGCATTCGCGGTGATCGCAGCAGTCTACGCTGCAGCTTCGGCCTTCCCGGCCAACGAGACTCTGGAGGTGGGCGCCAGCTGC-----------CTTCGGTCAGGTCAGCTCGGTGCTGAACAGCTGCTCCAACATCGT
X2         CTAGCATTCGCGGTGATCGCAGCAGTCTACGCTGCAGCTTCGGCCTTCCCGGCCAACGAGACTCTGGAGGTGGGCGCCAGCTGC-----------CTTCGGTCAGGTCAGCTCGGTGCTGAACAGCTGCTCCAACATCGT
X3         CTAGCATTCGCGGTAATCGCAGCAGTCTACGCTGCAGCTTCGGCCTTCCCGGCCAACGAGACTCTGGAGGTGGGCGCCAGCTGCACCA---------TCGGTCAGGTCAGCTCGGTGCTGAACAGCTGCTCCAACATCGT
X3         CTAGCATTCGCGGTAATCGCAGCAGTCTACGCTGCAGCTTCGGCCTTCCCAGCCAACGAGACTCTGGAGGTGGGCGCCAGCTGCACCA---------TCGGTCAGGTCAGCTCGGTGCTGAACAGCTGCTCCAACATCGT
X3         CTAGCATTCGCGGTAATCGCAGCAGTCTACGCTGCAGCTTCGGCCTTCCCGGCCAACGAGGCTCTGGAGGTGGGCGCCAGCTGCACCA---------TCGGTCAGGTCAGCTCGGTGCTGAACAGCTGCTCCAACATCGT
X3         CTAGCATTCGCGGTAATCGCAGTAGTCTACGCTGCAGCTTCGGCCTTCCCGGCCAACGAGACTCTGGAGGTGGGCGCCAGCTGCACCA---------TCGGTCAGGTCAGCTCGGTGCTGAACAGCTGCTCCAACATCGT
X3         CTAGCATTCGCGGTGATCGCAGCAGTCTACGCTGCAGCTTCGGCCTTCCCGGCCAACGAGACTCTGGAGGTGGGCGCCAGCTGC-----------CTTCGGTCAGGTCAGCTCGGTGCCGAACAGCTGCTCCAACATCGT
X3         CTAGCATTCGCGGTGATCGCAGCAGTCTACGCTGCAGCTTCGGCCTTCCCGGCCAACGAGACTCTGGAGGTGGGCGCCAGCTGC-----------CTTCGGTCAGGTCAGCTCGGTGCTGAACAGCTGCTCCAACATCGT
X3         CTAGCATTCGCGGTGATCGCAGCAGTCTACGCTGCAGCTTCGGCCTTCCCGGCCAACGAGACTCTGGAGGTGGGCGCCAGCTGC-----------CTTCGGTCAGGTCAGCTCGGTGCTGAACAGCTGCTCCAACATCGT
X4         CTAGCATTCGCGGTAATCGCAGCAGTCTACGCTGCAGCTTCGGCCTTCCCGGCCAACGAGACTCTGGAGGTGGGCGCCAGCTGCACCA---------TCGGTCAGGTCAGCTCGGTGCTGAACAGCTGCTCCAACATCGT
X4         CTAGCATTCGCGGTAATCGCAGCAGTCTACGCTGCAGCTTCGGCCTTCCCGGCCAACGAGACTCTGGAGGTGGGCGCCAGCTGCACCA---------TCGGTCAGGTCAGCTCGGTGCTGAACAGCTGCTCCAACATCGT
X4         CTAGCATTCGCGGTAATCGCAGCAGTCTACGCTGCAGCTTCGGCCTTCCCGGCCAACGAGACTCTGGAGGTGGGCGCCAGCTGCACCA---------TCGGTCAGGTCAGCTCGGTGCTGAACAGCTGCTCCAACACCGA
X4         CTAGCATTCGCGGTAATCGCAGCAGTCTACGCTGCAGCTTCGGCCTTCCCGGCCAACGAGACTCTGGAGGTGGGCGCCAGCTGCACCA---------TCGGTCAGGTCAGCTCGGTGCTGAACAGCTGCTCCAACATCGT
X4         CTAGCATTCGCGGTAATCGCAGCAGTCTACGCTGCAGCTTCGGCCTTCCCGGCCAACGAGACTCTGGAGGTGGGCGCCAGCTGC-----------CTTCGGTCAGGTCAGCTCGGTGCTGAACAGCTGCTCCAACATCGT
X4         CTAGCATTCGCGGTGATCGCAGCAGTCTACGCTGCAGCTTCGGCCTTCCCGGCCAACGAGACTCTGGAGGTGGGCGCCAGCTGC-----------CTTCGGTCAGGTCAGCTCGGTGCTGAACAGCTGCTCCAACATCGT
X4         CTAGCATTCGCGGTGATCGCAGCAGTCTACGCTGCAGCTTCGGCCTTCCCGGCCAACGAGACTCTGGAGGTGGGCGCCAGCTGC-----------CTTCGGTCAGGTCAGCTCGGTGCTGAACAGCTGCTCCAACATCGT
X4         CTAGCATTCGCGGTGATCGCAGCAGTCTACGCTGCAGCTTCGGCCTTCCCGGCCAGCGAGACTCTGGAGGTGGGCGCCAGCTGC-----------CTTCGGTCAGGTCAGCTCGGTGCTGAACAGCTGCTCCAACATCGT
X5         CTAGCATTCGCGGTAATCGCAGCAGTCTACGCTGCAGCTTCGGCCTTCCCGGCCAACGAGACTCTGGAGGTGGGCGCCAGCTGCACCA---------TCGGTCAGGTCAGCTCGGTGCTGAACAGCTGCTCCAACATCGT
X5         CTAGCATTCGCGGTAATCGCAGCAGTCTACGCTGCAGCTTCGGCCTTCCCGGCCAACGAGACTCTGGAGGTGGGCGCCAGCTGCACCA---------TCGGTCAGGTCAGCTCGGTGCTGAACAGCTGCTCCAACATCGT
X5         CTAGCATTCGCGGTAATCGCAGCAGTCTACGCTGCAGCTTCGGCCTTCCCGGCCAACGAGACTCTGGAGGTGGGCGCCAGCTGCACCA---------TCGGTCAGGTCAGCTCGGTGCTGAACAGCTGCTCCAACATCGT
X5         CTAGCATTCGCGGTAATCGCAGCAGTCTACGCTGCAGCTTCGGCCTTCCCGGCCAACGAGACTCTGGAGGTGGGCGCCAGCTGCACCA---------TCGGTCAGGTCAGCTCGGTGCTGAACAGCTGCTCCAACATCGT
X5         CTAGCATTCGCGGTGATCGCAGCAGTCTACGCTGCAGCTTCGGCCTTCCCGGCCAACGAGACTCTGGAGGTGGGCGCCAGCTGC-----------CTTCGGTCAGGTCAGCTCGGTGCTGAACAGCTGCTCCAACATCGT
X5         CTAGCATTCGCGGTGATCGCAGCAGTCTACGCTGCAGCTTCGGCCTTCCCGGCCAACGAGACTCTGGAGGTGGGCGCCAGCTGC-----------CTTCGGTCAGGTCAGCTCGGTGCTGAACAGCTGCTCCAACATCGT
X5         CTAGCCTTCGCGGTGATTGCAGCAGTCTACGCTGCAGCTTCGGCCTTCCCGGCCAACGAGACTCTGGAGGTGGGCGCCAGCTGC-----------CTTCGGTCAGGTCAACTCGGTGCTGAACAGCTGCTCCAACATCGT
X5         CTAGCATTCGCGGTGATCGCAGCAGTCTACGCTGCAGCTTCGGCCTTCCCGGCCAACGAGACTCTGGAGGTGGGCGCCAGCTGC-----------CTTCGGTCAGGTCAGCTCGGTGCTGAACAGCTGCTCCAACATCGT
X5         CTAGCATTCGCGGTGATCGCAGCAGTCTACGCTGCAGCTTCGGCCTTCCCGGCCAACGAGACTCTGGAGGTGGGCGCCAGCTGC-----------CTTCGGTCAGGTCAGCTCGGTGCTGAACAGCTGCTCCAACATCGT
X6         CTAGCATTCGCGGTAATCGCAGCAGTCTACGCTGCAGCTTCGGCCTTCCCGGCCAACGAGACTCTGGAGGTGGGCGCCAGCTGCACCA---------TCGGTCAGGTCAGCTCGGTGCTGAACAGCTGCTCCAACATCGT
X6         CTAGCATTCGCGGTAATCGCAGCAGTCTACGCTGCAGCTTCGGCCTTCCCGGCCAACGAGACTCTGGAGGTGGGCGCCAGCTGCACCA---------TCGGTCAGGTCAGCTCGGTGCTGAACAGCTGCTCCAACATCGT
X6         CTAGCATTCGCGGTAATCGCAGCAGTCTACGCTGCAGCTTCGGCCTTCCCGGCCAACGAGACTCTGGAGGTGGGCGCCAGCTGCACCA---------TCGGTCAGGTCAGCTCGGTGCTGAACAGCTGCTCCAACATCGT
X6         CTAGCATTCGCGGTAATCGCAGCAGTCTACGCTGCAGCTTCGGCCTTCCCGGCCAACGAGACTCTGGAGGTGGGCGCCAGCTGCACCA---------TCGGTCAGGTCAGCTCGGTGCTGAACAGCTGCTCCAACATCGT
X6         CTAGCATTCGCGGTAATCGCAGCAGTCTACGCTGCAGCTTCGGCCTTCCCGGCCAACGAGACTCTGGAGGTGGGCGCCAGCTGCACCA---------TCGGTCAGGTCAGCTCGGTGCTGAACAGCTGCTCCAACATCGT
X6         CTAGCATTCGCGGTAATCGCAGCAGTCTACGCTGCAGCTTCGGCCTTCCCGGCCAACGAGACTCTGGAGGTGGGCGCCAGCTGCACCA---------TCGGTCAGGTCAGCTCGGTGCTGAACAGCTGCTCCAACATCGT
X6         CTAGCATTCGCGGTGATCGCAGCAGTCTACGCTGCAGCTTCGGCCTTCCCGGCCAACGAGACTCTGGAGGTGGGCGCCAGCTGC-----------CTTCGGTCAGGTCAGCTCGGTGCTGAACAGCTGCTCCAACATCGT
X6         CTAGCATTCGCGGTGATCGCAGCAGTCTACGCTGCAGCCTCGGCCTTCCCGGCCAACGAGACTCTGGAGGTGGGCGCCAGCTGC-----------CTTCGGTCAGGTCAGCTCGGTGCTGAACAGCTGCTCCAACATCGT
X7         CTAGCATTCGCGGTAATCGCAGCAGTCTACGCTGCAGCTTCGGCCTTCCCGGCCAACGAGACTCTGGAGGTGGGCGCCAGCTGCACCA---------TCGGTCAGGTCAGCTCGGTGCTGAACAGCTGCTCCAACATCGT
X7         CTAGCCTTCGCGGTAATCGCAGCAGTCTACGCTGCAGCTTCGGCCTTCCCGGCCAACGAGACTCTGGAGGTGGGCGCCAGCTGCACCA---------TCGGTCAGGTCAGCTCGGTGCTGAACAGCTGCTCCAACATCGT
X7         CTAGCATTCGCGGTAATCGCAGCAGTCTACGCTGCAGCTTCGGCCTTCCCGGCCAACGAGACTCTGGAGGTGGGCGCCAGCTGCACCA---------TCGGTCAGGTCAGCTCGGTGCTGAACAGCTGCTCCAACATCGT
X7         CTAGCATTCGCGGTGATCGCAGCAGTCTACGCTGCAGCTTCGGCCTTCCCGGCCAACGAGACTCTGGAGGTGGGCGCCAGCTGC-----------CTTCGATCAGGTCAGCTCGGTGCTGAACAGCTGCTCCAACATCGT
X7         CTAGCATTCGCGGTGATCGCAGCAGTCTACGCTGCGGCTTCGGCCTTCCCGGCCAACGAGACTCTGGAGGTGGGCGCCAGCTGC-----------CTTCGGTCAGGTCAGCTCGGTGCTGAACAGCTGCTCCAACATCGT
X7         CTAGCATTCGCGGTGATCGCAGCAGTCTACGCCGCAGCTTCGGCCTTCCCGGCCAACGAGACTCTGGAGGTGGGCGCCAGCTGC-----------CTTCGGTCAGGTCAGCTCGGTGCTGAACAGCTGCTCCAACATCGT
X7         CTAGCATTCGCGGTGATCGCAGCAGTCTACGCTGCAGCTTCGGCCTTCCCGGCCAACGAGACTCTGGAGGTGGGCGCCAGCTGC-----------CTTCGGTCAGGTCAGCTCGGTGCTGAACAGCTGCTCCAACATCGT
X8         CTAGCATTCGCGGTAATCGCAGCAGTCTACGCTGCAGCTTCGGCCTTCCCGGCCAACGAGACTCTGGAGGTGGGCGCCAGCTGCACCA---------TCGGTCAGGTCAGCTCGGTGCTGAACAGCTGCTCCAACATCGT
X8         CTAGCATTCGCGGTAATCGCAGCAGTCTACGCTGCAGCTTTGGCCTTCCCGGCCAACGAGACTCTGGAGGTGGGCGCCAGCTGCACCA---------TCGGTCAGGTCAGCTCGGTGCTGAACAGCTGCTCCAACATCGT
X8         CTAGCATTCGCGGTAATCGCAGCAGTCTACGCTGCAGCTTCGGCCTTCCCGGCCAACGAGACTCTGGAGGTGGGCGCCAGCTGCACCA---------TCGGTCAGGTCAGCTCGGTGCTGAACAGCTGCTCTAACATCGT
X8         CTAGCCTTCGCGGTGATCGCAGCAGTCTACGCTGCAGCTTCGGCCTTCCCGGCCAACGAGACTCTGGAGGTGGGCGCCAGCTGC-----------CTTCGGTCAGGTCAGCTCGGTGCTGAACAGCTGCTCCAACATCGT
X8         CTAGCATTCGCGGTGATCGCAGCAGTCTACGCTGCAGCTTCGGCCTTCCCGGCCAACGAGACTCTGGAGGTGGGCGCCAGCTGC-----------CTTCGGTCAGGTCAGCTCGGTGCTGAACAGCTGCTCCAACATCGT
X8         CTAGCATTCGCGGTGATCGCAGCAGTCTACGCTGCAGCTTCGGCCTTCCCGGCCAACGAGACTCTGGAGGTGGGCGCCAGCTGC-----------CTTCGGTCAGGTCAGCTCGGTGCTGAACAGCTGCTCCAACATCGT
X8         CTAGCATTCGCGGTGATCGCAGCAGTCTACGCTGCAGCTTCGGCCTTCCCGGCCAACGAGACTCTGGAGGTGGGCGCCAGCTGC-----------CTTCGGTCAGGTCAGTTCGGTGCTGAACAGCTGCTCCAACATCGT
X8         CTAGCATTCGCGGTGATCGCAGCAGTCTACGCTGCAGCTTCGGCCTTCCCGGCCAACGAGACTCTGGAGGTGGGCGCCAGCTGC-----------CTTCGGTCAGGTCAGCTCGGTGCTGAACAGCTGCTCCAACATCGT
Y1         CTAGCATTCGCGGTAATCGCAGCAGTCTACGCTGCAGCTTCGGCCTTCCCGGCCAACGAGACTCTGGAGGTGGGCGCCAGCTGCACCA---------TCGGTCAGGTCAGCTCGGTGCTGAACAGCTGCTCCAACATCGT
Y1         CTAGCATTCGCGGTAATCGCAGCAGTCTACGCTGCAGCTTCGGCCTTCCCGGCCAACGAGACTCTGGAGGTGGGCGCCAGCTGCACCA---------TCGGTCAGGTCAGCTCGGTGCTGAACAGCTGATCCAACATCGT
Y1         CTAGCATTCGCGATGATCGCAGCAGTCTACGCTGCAGCTTCGGCCTTCCCGGCCAACGAGACTCTGGAGGTGGGCGCCAGCTGC-----------CTTCGGTCAGGTCAGCTCGGTGCTGAACAGCTGCTCCAACATCGT
Y1         CTAGCATTCGCGGTAATCGCAGCAGTCTACGCTGCAGCTTCGGCCTTCCCGGCCAACGAGACTCTGGAGGTGGGCGCCAGCTGC-----------CTTCGGTCAGGTCAGCTCGGTGCTGAACAGCTGCTCCAACATCGT
Y1         CTAGCATTCGCGGTGATCGCAGCAGTCCACGCTGCAGCTTCGGCCTTCCCGGCCAACGAGACTCTGGAGGTGGGCGCCAGCTGC-----------CTTCGGTCAGGTCAGCTCGGTGCTGAACAGCTGCTCCAACATCGT
Y1         CTAGCATTCGCGGTGATCGCAGCAGTCTACGCTGCAGCTTCGGCCTTCCCGGCCAACGAGACTCTGGAGGTGGGCGCCAGCTGC-----------CTTCGGTCAGGTCAGCTCGGTGCTGAACAGCTGCTCCAACATCGT
Y1         CTAGCATTCGCGGTGATCGCAGCAGTCTACGCTGCAGCTTCGGCCTTCCCGGCCAACGAGACTCTGGAGGTGGGCGCCAGCTGC-----------CTTCGGTCAGGTCAGCTCGGTGCTGAACAGCTGCTCCAACATCGT
Y1         CTAGCATTCGCGGTGATCGCAGCAGTCTACGCTGCAGCTTCGGCCTTCCCGGCCAACGAGACTCTGGAGGTGGGCGCCAGCTGC-----------CTTCGGTCAGGTCAGCTCGGTGCTGAACAGCTGCTCCAACATCGT
Y2         CTAGCATTCGCGGTAATCGCAGCAGTCTACGCTGCAGCTTCGGCCTTCCCGGCCAACGGGACTCTGGAGGTGGGCGCCAGCTGCACCA---------TCGGTCAGGTCAGCTCGGTGCTGAACAGCTGCTCCAACATCGT
Y2         CTAGCATTCGCGGTAATCGCAGCAGTCTACGCTGCAGCTTCGGCCTTCCCGGCCAACGAGACTCTGGAGGTGGGCGCCAGCTGCACCA---------TCGGTCAGGTCAGCTCGGTGCTGAACAGCTGCTCCAACATCGT
Y2         CTAGCATTCGCGGTAATCGCAGCAGTCTACGCTGCAGCTTCGGCCTTCCCGGCCAACGGGACTCTGGAGGTGGGCGCCAGCTGCACCA---------TCGGTCAGGTCAGCTCGGTGCTGAACAGCTGCTCCAACATCGT
Y2         CTAGCATTCGCGGTAATCGCAGCAGTCTACGCTGCAGCTTCGGCCTTCCCGGCCAACGAGACTCTGGAGGTGGGCGCCAGCTGCACCA---------TCGGTCAGGTCAGCTCGGTGCTGAACAGCTGCTCCAACATCGT
Y2         CTAGCATTCGCGGTAATCGCAGCAGTCAACGCTGCAGCTTCGGCCTTCCCGGCCAACGAGACTCTGGAGGTGGGCGCCAGCTGCACCA---------TCGGTCAGGTCAGCTCGGTGCTGAACAGCTGCTCCAACATCGT
Y2         CTAGCATTCGCGGTAATCGCAGCAGTCTACGCTGCAGCTTCGGCCTTCCCGGCCAACGAGACTCTGGAGGTGGGCGCCAGCTGCACCA---------TCGGTCAGGTCAGCTCGGTGCTGAACAGCTGCTCCAACATCGT
Y2         CTAGCATTCGCGGTGATCGCAGCAGTCTACGCTGCAGCTTCGGCCTTCCCGGCCAACGAGACTCTGGAGGTGGGCGCCAGCTGC-----------CTTCGGTCAGGTCAGCTCGGTGCTGAACAGCTGCTCCAACATCGT
Y2         CTAGCATTCGCGGTGATCGCAGCAGTCTACGCTGCAGCTTCGGCCTTCCCGGCCAACGAGACTCTGGAGGTGGGCGCCAGCTGC-----------CTTCGGTCAGGTCAGCTCGGTGCTGAACAGCTGCTCCAACATCGT
Y2         CTAGCATTCGCGGTGATCGCAGCAGTCTACGCTGCAGCTTCGGCCTTCCCGGCCAACGAGACCCTGGAGGTGGGCGCCAGCTGC-----------CTTCGGTCAGGTCAGCTCGGTGCTGAACAGCTGCTCCAACATCGT
Y3         CTAGCATTCGCGGTAATCGCAGCAGTCTACGCTGCAGCTTCGGCCTTCCCGGCCAACGAGACTCTGGAGGTGGGCGCCAGCTGCACCA---------TCGGTCAGGTCAGCTCGGTGCTGAACAGCTGCTCCAACATCGT
Y3         CTAGCATTCGCGGTAATCGCAGCAGTCTACGCTGCAGCTTCGGCCTTCCCGGCCAACGAGACTCTGGAGGTGGGCGCCAGCTGCACCA---------TCGGTCAGGTCAGCTCGGTGCTGAACAGCTGCTCCAACATCGT
Y3         CTAGCATTCGCGGTAATCGCAGCAGTCTACGCTGCAGCTTCGGCCTTCCCGGCCAACGAGACTCTGGGGGTGGGCGCCAGCTGCACCA---------TCGGTCAGGTCAGCTCGGTGCTGAACAGCTGCTCCAACATCGT
Y3         CTAGCATTCGCGGTAATCGCAGCAGTCTACGCTGCAGCTTCGGCCTTCCCGGCCAACGAGACTCTGGAGGTGGGCGCCAGCTGCACCA---------TCGGTCAGGTCAGCTCGGTGCTGAACAGCTGCTCCAACATCGT
Y3         CTAGCATTCGCGGTAATCGCAGCAGTCTACGCTGCAGCTTCGGCCTTCCCGGCCAACGAGACTCTGGAGGTGGGCGCCAGCTGCACCA---------TCGGTCAGGTCAGCTCGGTGCTGAACAGCTGCTCCAACATCGT
Y3         CTAGCATTCGCGGTGATCGCAGCAGTCTACACTGCAGCTTCGGCCTTCCCGGCCAACGAGACTCTGGAGGTGGGCGCCAGCTGC-----------CTTCGGTCAGGTCAGCTCGGTGCTGAACAGCTGCTCCAACATCGT
Y3         CTAGCATTCGCGGTGATCGCAGCAGTCTACGCTGCAGCTTCGGCCTTCCCGGCCAACGAGACTCTGGAGGTGGGCGCCAGCTGC-----------CTTCGGTCAGGTCAGCTCGGTGCTGAACAGCTGCTCCAACATCGT
Y3         CTAGCATTCGCGGTGATCGCAGCAGTCTACGCTGCAGCTTCGGCCTTCCCGGCCAACGAGACTCTGGAGGTGGGCGCCAGCTGC-----------CTTCGGTCAGGTCAGCTCGGTGCTGAACAGCTGCTCCAACATCGT
Y3         CTAGCATTCGCGGTGATCGCAGCAGTCTACGCTGCAGCTTCGGCCTTCCCGGCCAACGAGACTCTGGAGGTGGGCGCCAGCTGC-----------CTTCGGTCAGGTCAGCTCGGTGCTGAACAGCTGCTCCAACATCGT
Y4         CTAGCATTCGCGGTAATCGCAGCAGTCTACGCTGCAGCTTCGGCCTTCCCGGCCAACGAGACTCTGGAGGTGGGCGCCAGCTGCACCA---------TCGGTCAGGTCAGCTCGGTGCTGAACAGCTGCTCCAACATCGT
Y4         CTAGCATTCGCGGTAATCGCAGCAGTCTACGCTGCAGCCTCGGCCTTCCCGGCCAACGAGACTCTGGAGGTGGGCGCCAGCTGCACCA---------TCGGTCAGGTCAGCTCGGTGCTGAACAGCTGCTCCAACATCGT
Y4         CTAGCATTCGCGGTAATCGCAGCAGTCTACGCTGCAGCTTCGGCCTTCCCGGCCAACGAGACTCTGGAGGTGGGCGCCAGCTGCACCA---------TCGGTCAGGTCAGCTCGGTGCTGAACAGCTGCTCCAACATTGT
Y4         CTAGCATTCGCGGTAATCGCAGCAGTCTACGCTGCAGCTTCGGCCTTCCCGGCCAACGAGACTCTGGAGGTGGGCGCCAGCTGCACCA---------TCGGTCAGGTCAGCTCGGTGCTGAACAGCTGCTCCAACATCGT
Y4         CTAGCATTCGCGGTAATCGCAGCAGTCTACGCTGCAGCTTCGGCCTTCCCGGCCAACGAGACTCTGGAGGTGGGCGCCAGCTGCACCA---------TCGGTCAGGTCAGCTCGGTGCTGAACAGCTGCTCCAACATCGT
Y4         CTAGCATTCGCGGTAATCGCAGCAGTCTACGCTGCAGCTTCGGCCTTCCCGGCCAACGAGACTCTGGAGGGGGGCGCCAGCTGCACCA---------TCGGTCAGGTCAGCTCGGTGCTGAACAGCTGCTCCAACATCGT
Y4         CTAGCATTCGCGGTAATCGCAGCAGTCTACGCTGCAGCTTCGGCCTTCCCGGCCAACGAGACTCTGGAGGTGGGCGCCAGCTGCACCA---------TCGGTCAGGTCAGCTCGGTGCTGAACAGCTGCTCCAACATCGT
Y4         CTAGCATTCGCGGTGATCGCAGCAGTCTACGCTGCAGCTTCGGCCTTCCCGGCCAACGAGACTCTGGAGGTGGGCGCCAGCTGC-----------CTTCGGTCAGGTCAGCTCGGTGCTGAACAGCTGCTCCAACATCGT
Y5         CTAGCATTCGCGGTAATCGCAGCAGTCTACGCTGCAGCTTCGGCCTTCCCGGCCAACGAGACTCTGGAGGTGGGCGCCAGCTGCACCA---------TCGGTCAGGTCAGCTCGGTGCTGAACAGCTGCTCCAACATCGT
Y5         CTAGCATTCGCGGCAATCGCAGCAGTCTACGTTGCAGCTTCGGCCTTCCCGGCCAACGAGACTCTGGAGGTGGGCGCCAGCTGCACCA---------TCGGTCAGGTCAGCTCGGTGCTGAACAGCTGCTCCAACATCGT
Y5         CTAGCATTCGCGGTAATCGCAGCAGTCTACGCTGCAGCTTCGGCCTTCCCGGCCAACGAGACTCTGGAGGTGGGCGCCAGCTGCACCA---------TCGGTCAGGTCAGCTCGGTGCTGAACAGCTGCTCCAACATCGT
Y5         CTAGCATTCGCGGTGATCGCAGCAGTCTACGCTGCAGCTTCGGCCTTCCCGGCCAACGAGACTCTGGAGGTGGGCGCCAGCTGCACCA---------TCGGTCAGGTCAGCTCGGTGCTGAACAGCTGCTCCAACATCGT
Y5         CTAGCATTCGCGGTAATCGCAGCAGTCTACGCTGCAGCTTCGGCCTTCCCGGCCAACGAGACTCTGGAGGTGGGCGCCAGCTGCACCA---------TCGGTCAGGTCAGCTCGGTGCTGAACAGCTGCTCCAACATCGT
Y5         CTAGCATTCGCGGTAATCGCAGCAGTCTACGCTGCAGCTTCGGCCTTCCCGGCCAACGAGACTCTGGAGGTGGGCGCCAGCTGCACCA---------TCGGTCAGGTCAGCTCGGTGCTGAACAGCTGCTCCAACATCGT
Y5         CTAGCATTCGCGGTGATCGCAGCAGTCTACGCTGCAGCTTCGGCCTTCCCGGCCAACGAGACTCTGGAGGTGGGCGCCAGCTGC-----------CTTCGGTCAGGTCAGCTCGGTGCTGAACAGCTGCTCCAACATCGT
Y5         CTAGCATTCGCGGTGATCGCAGCAGCCTACGCTGCAGCTTCGGCCTTCCCGGCCAACGAGACTCTGGAGGTGGGCGCCAGCTGC-----------CTTCGGTCAGGTCAGCTCGGTGCTGAACAGCTGCTCCAACATCGT
```

C continued

```
        L  A  F  A  V  I  A  A  V  Y  A  A  A  S  A  F  P  A  N  E  T  L  E  V  G  A  S  C  T  I  T  S  F  G  Q  V  S  S  V  L  N  S  C  S  N  I
                                                                                             28-5/9E1 target
28-9E1       CTAGCATTCGCGGTAATCGCAGCAGTCTACGCTGCAGCTTCGGCCTTCCCGGCCAACGAGACTCTGGAGGTGGGCGCCAGCTGCACCATCACCAGCTTCGGTCAGGTCAGCTCGGTGCTGAACAGCTGCTCCAACATCGT
Y6   28-9 |  CTAGCATTCGCGGTAATCGCAGCAGTCTACGCTGCAGCTTCGGCCTTCCCGGCCAACGAGACTCTGGAGGTGGGCGCCAGCTGCACCA---------TCGGTCAGGTCAGCTCGGTGCTGAACAGCTGCTCCAACATCGT
Y6        |  CTAGCATTCGCGGTAATCGCAGCAGTCTACGCTGCAGCTTCGGCCTTCCCGGCCAACGAGACTCTGGAGGTGGGCGCCAGCTGCACCA---------TCGGTCAGGTCAGCTCGGTGCTGAACAGCTGCTCCAACATCGT
Y6  28-10 |  CTAGCATTCGCGGTGATCGCAGCAGTCTACGCTGCAGCTTCGGCCTTCCCGGCCAACGAGACTCTGGAGGTGGGCGCCAGCTGC-----------CTTCGGTCAGGTCAGCTCGGTGCTGAACAGCTGCTCCAACATCGT
Y6        |  CTAGCATTCGCGGTGATCGCAGCAGTCTACGCTGCAGCTTCGGCCTTCCCGGCCAACGAGACTCTGGAGGTGGGCGCCAGCTGC-----------CTTCGGTCAGGTCAGCTCGGTGCTGAACAGCTGCTCCAACATCGT
Y6        |  CTAGCATTCGCGGTGATCGCAGCAGTCTACGCTGCAGCTTCGGCCTTCCCGGCCAACGAGACTCTGGAGGTGGGCGCCAGCTGC-----------CTTCGGTCAGGTCAGCTCGGTGCTGAACAGCTGCTCCAACATCGT
Y6        |  CTAGCATTCGCGGTGATCGCAGCAGTCTACGCTGCAGCTTCGGCCTTCCCGGCCAACGAGACTCTGGAGGTGGGCGCCAGCTGC-----------CTTCGGTCAGGTCAGCTCGGTGCTGAACAGCTGCTCCAACATCGT
Y6        |  CTAGCATTCGCGGTGATCGCAGCAGTCTACGCTGCAGCTTCGGCCTTCCCGGCCAACGAGACTCTGGAGGTGGGCGCCAGCTGC-----------CTTCGGTCAGGTCAGCTCGGTGCTGAACAGCTGCTCCAACATCGT
Y7           CTAGCCTTCGCGGT-ATCGCAGCAGTCTACGCTGCAGCTTCGGCCTTCCCGGCCAACGAGACTCTGGAGGTGGGCGCCAGCTGCACCA---------TCGGTCAGGTCAGCTCGGTGCTGAACAGCTGCTCCAACATCGT
Y7           CTAGCATTCGCGGTGATCGCAGCAGTCTACGCTGCAGCTTCGGCCTTCCCGGCCAACGAGTCTCTGGAGGTGGGCGCCAGCTGCACCA---------TCGGTCAGGTCAGCTCGGTGCTGAACAGCTGCTCCAACATCGT
Y7           CTAGCATTCGCGGTAATCGCAGCAGTCTACGCTGCAGCTTCGGCCTTCCCGGCCAACGAGACTCTGGAGGTGGGCGCCAGCTGCACCA---------TCGGTCAGGTCAGCTCGGTGCTGAGCAGCTGCTCCAACATCGT
Y7           CTAGCATTCGCGGTAATCGCAGCAGTCTACGCTGCAGCTTCGGCCTTCCCGGCCAACGAGACTCTGGAGGTGGGCGCCAGCTGCACCG---------TCGGTCAGGTCAGCTCGGTGCTGAACAGCTGCTCCAACATCGT
Y7           CTAGCATTCGCGGTAATCGCAGCAGTCTACGCTGCAGCTTCGGCCTTCCCGGCCAACGAGACTCTGGAGGTGGGCGCCAGCTGCACCA---------TCGGTCAGGTCAGCTCGGTGCTGAACAGCTGCTCCAACATCGT
Y7           CTAGCATTCGCGGTGATCGCAGCAGTCTATGCTGCAGCTTCGGCCTTCCCGGCCAACGAGACTCTGGAGGTGGGCGCCAGCTGC-----------CTTCGGTCAGGTCAGCTCGGTGCTGAACAGCTGCTCCAACATCGT
Y7           CTAGCATTCGCGGTGATCGCAGCAGTCTACGCTGCAGCTTCGGCCTTCCCGGCCAACGAGACTCTGGAGGTGGGCGCCAGCTGC-----------CTTCGGTCAGGTCAGCTCGGTGCTGAACAGCTGCTCCAACATCGT
Y7           CTAGCATTCGCGGTGATCGCAGCAGTCTACGCTGCAGCTTCGGCCTTCCCGGCCAACGAGACTCTGGAGGTGGGCGCCAGCTGC-----------CTTCGGTCAGGTCAGCTCGGTGCTGAACAGCTGCTCCAACATCGT
Y8           CTAGCATTCGCGGTAATCGCAGCAGTCTACGCTGCAGCTTCGGCCTTCCCGGCCAACGAGACTCTGGAGGTGGGCGCCAGCTGCGCCA---------TCGGTCAGGTCAGCTCGGTGCTGAACAGCTGCTCCAACATCGT
Y8           CTAGCATTCGCGGTAATCGCAGCAGTCTACGCTGCAGCTTCGGCCTTCCCGGCCAACGAGACTCTGGAGGTGGGCGCCAGCTGCACCA---------TCGGTCAGGTCAGCTCGGTGCTGAACAGCTGCTCCAACATCGT
Y8           CTAGCATTCGCGGTAATCGCAGCAGTCTACGCTGCAGCTTCGGCCTTCCCGGCCAACGAGACTCTGGAGGTGGGCGCCAGCTGCACCA---------TCGGTCAGGTCAGCTCGGTGCTGAACAGCTGCCCCAACATCGT
Y8           CTAGCATTCGCGGTGATCGCAGCAGTCTACGCTGCAGCTTCGGCCTTCCCGGCCAACGAGACTCTGGAGGTGGGCGCCAGCTGC-----------CTTCGGTCAGGTCAGCTCGGTGCTGAACAGCTGCTCCAACATCGT
Y8           CTAGCATTCGCGGTGATCGCAGCAGTCTACGCTGCAGCTTCGGCCTTCCCGGCCAACGAGACTCTGGAGGTGGGCGCCAGCTGC-----------CTTCGGTCAGGTCAGCTCGGTGCTGAACAGCTGCTCCAACATCGT
Y8           CTAGCATTCGCGGTGATCGCAGCAGTCTACGCTGCAGCTTCGGCCTTCCCGGCCAACGAGACTCTGGAGGTGGGCGCCAGCTGC-----------CTTCGGTCAGGTCAGCTCGGTGCTGAACAGCTGCTCCAACATCGT
Y8           CTAGCATTCGCGGTGATCGCAGCAGTCTACGCTGCAGCTTCGGCCTTCCCGGCCAACGAGACTCTGGAGGTGGGCGCCAGCTGC-----------CTTCGGTCAGGTCAGCTCGGTGCTGAACAGCTGCTCCAACATCGT
Y8           CTAGCATTCGCGGTGATCGCAGCAGTCTACGCTGCAGCTTCGGCCTTCCCGGCCAACGAGACTCTGGAGGTGGGCGCCAGCTGC-----------CTTCGGTCAGGTCAGCTCGGTGCTGAACAGCTGCTCCAACATCGT
Z1           CTAGCATTCGCGGTAATCGCAGCAGTCTACGCTGCAGCTTCGGCCTTCCCGGCCAACGAGACTCTGGAGGTGGGCGCCAGCTGCACCA---------TCGGTCAGGTCAGCTCGGTGCTGAACAGCTGCTCCAACATCGT
Z1           CTAGCATTCGCGGTAATCGCAGCAGTCTACGCTGCAGCTTCGGCCTTCCCGGCCAACGAGACTCTGGAGGTGGGCACCAGCTGCACCA---------TCGGTCAGGTCAGCTCGGTGCTGAACAGCTGCTCCAACATCGT
Z1           CTAGCATTCGCGGTAATCGCAGCAGTCTACGCTGCAGCTTCGGCCTTCCCGGCCAACGAGACTCTGGAGGTGGGCGCCAGCTGCACCA---------TCGGTCAGGTCAGCTCGGTGCTGAACAGCTGCTCCAACATCGT
Z1           CTAGCATTCGCGGTAATCGCAGCAGTCTACGCTGCAGCTTCGGCCTTCCCGGCCAACGAGACTCTGGAGGTGGGCGCCAGCTGCACCA---------TCGGTCAGGTCAGCTCGGTGCTGAACAGCTGCTCCAACATCGT
Z1           CTAGCATTCGCGGTAATCGCAGCAGTCTACGCTGCAGCTTCGGCCTTCCCGGCCAACGAGACTCTGGAGGTGGGCGCCAGCTGCACCA---------TCGGTCAGGTCAGCTCGGTGCTGAACAGCTGCTCCAACATCGT
Z1           CTAGCATTCGCGGTAATCGCAGCAGTCTACGCTGCAGCTTCGGCCTTCCCGGCCAACGAGACTCTGGAGGTGGGCGCCAGCTGCACCA---------TCGGTCAGGTCAGCTCGGTGCTGAACAGCTGCTCCAACATCGT
Z1           CTAGCATTCGCGGTAATCGCAGCAGTCTACGCTGCAGCTTCGGCCTTCCCGGCCAACGAGACTCTGGAGGTGGGCGCCAGCTGCACCA---------TCGGTCAGGTCAGCTCGGTGCTGAACAGCTGCTCCAACATCGT
Z1           CTAGCATTCGCGGTGATCGCAGCAGTCTACGCTGCAGCTTCGGCCTTCCCGGCCAACGAGACTCTGGAGGTGGGCGCCAGCTGC-----------CTTCGGTCAGGTCAGCTCGGTGCTGAACAGCTGCTCCAACATCGT
Z2           CTAGCATTCGCGGTAATCGCAGCAGTCTACGCTGCAGCTTCGGCCTTCCCGGCCAACGAGACTCTGGAGGTGGGCGCCAGCTGCACCA---------TCGGTCAGGTCAGCTCGGTGCTGAACAGCTGCTCCAACATCGT
Z2           CTAGTATTCCCGGTAATCGCAGCAGTCTACGCTGCAGCTTCGGCCTTCCCGGCCAACGAGACTCTGGAGGTGGGCGCCAGCTGGACCA---------TCGGTCAGGTCAGCTCGGTGCTGAACAGCTGCTCCAACATCGT
Z2           CTAGCATTCGCGGTAATCGCAGCAGTCTACGCTGCAGCTTCGGCCTTCCCGGCCAACGAGACTCTGGAGGTGGGCGCCAGCTGCACCA---------TCGGTCAGGTCAGCTCGGTGCTGAACAGCTGCTCCAACATCGT
Z2           CTAGCATTCGCGGTAATCGCAGCAGTCTACGCTGCAGCTTCGGCCTTCCCGGCCAACGAGACTCTGGAGGTGGGCGCCAGCTGCACCA---------TCGGTCAGGTCAGCTCGGTGCTGAACAGCTGCTCCAACATCGT
Z2           CTAGCATTCGCGGTAATCGCAGCAGTCTACGCTGCAGCCTCGGCCTTCCCGGCCAACGAGACTCTGGAGGTGGGCGCCAGCTGCACCA---------TCGGTCAGGTCAGCTCGGTGCTGAACAGCTGCTCCAACATCGT
Z2           CTAGCATTCGCGGTAATCGCAGCAGTCTACGCTGCAGCTTCGGCCTTCCCGGCCAACGAGACTCTGGAGGTGGGCGCCAGCTGCACCA---------TCGGTCAGGTCAGCTCGGTGCTGAACAGCTGCTCCAACATCGT
Z2           CTAGCATTCGCGGTGATCGCAGCAGTCTACGCTGCAGCTTCGGCCTTCCCGGCCAACGAGACTCTGGAGGTGGGCGCCAGCTGC-----------CTTCGGTCAGGTCAGCTCGGTGCTGAATAGCTGCTCCAACATCGT
Z2           CTAGCATTCGCGGTGATCGCAGCAGTCTACGCTGCAGCTTCGGCCTTCCCGGCCAACGAGACTCTGGAGGTGGGCGCCAGCTGC-----------CTTCGGTCAGGTCAGCTCGGTGCTGAACAGCTGCTCCAACATCGT
Z3           CTAGCATTCGCGGTAATCGCAGCAGTCTACGCTGCAGCTTCGGCCTTCCCGGCCAACGAGACTCTGGAGGTGGGCGCCAGCTGCACCA---------TCGGTCAGGTCAGCTCGGTGCTGAACAGCTGCTCCAACATCGT
Z3           CTAGCATTCGCGGTAATCGCAGCAGTCTACGCTGCAGCTTCGGCCTTCCCGGCCAACGAGACTCTGGAGGTGGGCGCCAGCTGCACCA---------TCGGTCAGGTCAGCTCGGTGCTGAACAGCTGCTCCAACATCGT
Z3           CTAGCATTCGCGGTAATCGCAGCAGTCTACGCTGCAGCTTCGGCCTTCCCGGCTAACGAGACTCTGGAGGTGGGCGCCAGCTGCACCA---------TCGGTCAGGTCAGCTCGGTGCTGAACAGCTGCTCCAACATCGT
Z3           CTAGCATTCGCGGTAATCGCAGCAGTCTACGCTGCAGCTTCGGCCTTCCCGGCCAACGAGACTCTGGAGGTGGGCGCCAGCTGCACCA---------TCGGTCAGGTCAGCTCGGTGCTGAACAGCTGCTCCAACATCGT
Z3           CTAGCATTCGCGGTAATCGCAGCAGTCTACGCTGCAGCTTCGGCCTTCCCGGCCAACGAGACTCTGGAGGTGGGCGCCAGCTGCACCA---------TCGGTCAGGTCAGCTCGGTGCTGAACAGCTGCTCCAACATCGT
Z3           CTAGCATTCGCGGTAATCGCAGCAGTCTACGCTGCAGCTTCGGCCTTCCCGGCCAACGAGACTCTGGAGGTGGGCGCCAGCTGCACCA---------TCGGTCAGGTCAGCTCGGTGCTGAACAGCTGCTCCAACATCGT
Z3           CTAGCATTCGCGGTAATCGCAGCAGTCTACGCTGCAGCTTCGGCCTTCCCGGCCAACGAGACTCTGGAGGTGGGCGCCAGCTGCACCA---------TCGGTCAGGTCAGCTCGGTGCTGAACAGCTGCTCCAACATCGT
Z3           CTAGCATTCGCGGTGATCGCAGCAGTCTACGCTGCAGCTTCGGCCTTCCCGGCCAACGAGACTCTGGAGGTGGGCGCCAGCTGC-----------CTTCGGTCAGGTCAGCTCGGTGCTGAACAGCTGCTCCAACATCGT
Z4           CTAGCATTCGCGGTAATCGCAGCAGTCTACGCTGCAGCTTCGGCCTTCCCGGCCAACGAGACTCTGGAGGTGGGCGCCAGCTGCACCA---------TCGGTCAGGTCAGCTCGGTGCTGAACAGCTGCTCCAACATCGT
Z4           CTAGCATTCGCGGTAATCGCAGCAGTCTACGCTGCAGCTTCGGCCTTCCCGGCCAACGAGACTCTGGAGGTGGGCGCCAGCTGCACCA---------TCGGTCAGGTCAGCTCGGTGCTGAACAGCTGCTCCAACATCGT
Z4           CTAGCATTCGCGGTAATCGCAGCAGTCTACGCTGCAGCTTCGGCCTTCCCGGCCGACGAGACTCTGGAGGTGGGCGCCAGCTGCACCA---------TCGGTCAGGTCAGCTCGGTGCTGAACAGCTGCTCCAACATCGT
Z4           CTAGCATTCGCGGTGATCGCAGCAGTCTACGCTGCAGCTTCGGCCTTCCCGGCCAACGAGACTCTGGAGGTGGGCGCCAGCTGCACCA---------TCGGTCAGGTCAGCTCGGTGCTGAACAGCTGCTCCAACATCGT
Z4           CTAGCCTTCGCGGTGATCGCAGCAGTCTACGCTGCAGCTTCGGCCTTCCCGGCCAACGAGACTCTGGAGGTGGGCGCCAGCTGC-----------CTTCGGTCAGGTCAGCTCGGTGCTGAACAGCTGCTCCAACATCGT
Z4           CTAGCATTCGCGGTGATCGCAGCAGTCTACGCTGCAGCTTCGGCCTTCCCGGCCAACGAGACTCTGGAGGTGGGCGCCAGCTGC-----------CTTCGGTCAGGTCAGCTCGGTGCTGAACAGCTGCTCCAACATCGT
Z4           CTAGCATTCGCGGTGATCGCAGCAGTCTACGCTGCAGCTTCGGCCTTCCCGGCCAACGAGACTCTGGAGGTGGGCGCCAGCTGC-----------CTTCGGTCAGGTCAGCTCGGTGCTGAACAGCTGCTCCAACATCGT
Z4           CTAGCATTCGCGGTGATCGCAGCAGTCTACGCTGCAGCTTCGGCCTTCCCGGCCAACGAGGCTCTGGAGGTGGGCGCCAGCTGC-----------CTTCGGTCAGGTCAGCTCGGTGCTGAACAGCTGCTCCAACATCGT
Z5           CTAGCATTCGCGGTAATCGCAGCAGTCTACGCTGCAGCTTCGGCCTTCCCGGCCAACGAGACTCTGGAGGTGGGCGCCAGCTGCACCA---------TCGGTCAGGTCATCTCGGTGCTGAACAGCTGCTCCAACATCGT
Z5           CTAGCATTCGCGGTAATCGCAGCAGTCTACGCTGCAGCTTCGGCCTTCCCGGCCAACGAGACTCTGGAGGTGGGCGCCAGCTGCACCA---------TCGGTCAGGTCAGCTCGGTGCTGAACAGCTGCTCCAACATCGT
Z5           CTAGCATTCGCGGTAATCGCAGCAGTCTACGCTGCAGCTTCGGCCTTCCCGGCCAACGAGACTCTGGAGGTGGGCGCCAGCTGCACCA---------TCGGTCAGGTCAGCTCGGTGCTGAACAGCTGCTCCAACATCGT
Z5           CTAGCATTCGCGGTAATCGCAGCAGTCTACGCTGCAGCTTCGGCCTTCCCGGCCAACGAGACTCTGGAGGTGGGCGCCAGCTGCACCA---------TCGGTCAGGTCAGCTCGGTGCTGAACAGCTGCTCCAACATCGT
Z5           CTAGCATTCGCGGTAATCGCAGCAGTCTACGCTGCAGCTTCGGCCTTCCCGGCCAACGAGACTCTGGAGGTGGGCGCCAGCTGCACCA---------TCGGTCAGGTCAGCTCGGTGCTGAACAGCTGCTCCAACATCGT
Z5           CTAGCATTCGCGGTAATCGCAGCAGTCTACGCTGCAGCTTCGGCCTTCCCGGCCAACGAGACTCTGGAGGTGGGCGCCAGCTGCACCA---------TCGGTCAGGTCAGCTCGGTGCTGAACAGCTGCTCCAACATCGT
Z5           CTAGCATTCGCGGTAATCGCAGCAGTCTACGCTGCAGCTTCGGCCTTCCCGGCCAACGAGACCCTGGAGGTGGGCGCCAGCTGCACCA---------TCGGTCAGGTCAGCTCGGTGCTGAACAGCTGCTCCAACATCGT
Z5           CTAGCATTCGCGGTGATCGCAGCAGTCTACGCTGCAGCTTCGGCCTTCCCGGCCAACGAGACTCTGGAGGTGGGCGCCAGCTGC-----------CTTCGGTCAGGTCAGCTCGGTGCTGAACAGCTGCTCCAACATCGT
Z6           CTAGCATTCGCGGTAATCGCAGCAGTCTACGCTGCAGCTTCGGCCTTCCCGGCCAGCGAGACTCTGGAGGTGGGCGCCAGCTGCACCA---------TCGGTCAGGTCAGCTCGGTGCTGAACAGCTGCTCCAACATCGT
Z6           CTAGCATTCGCGGTAATCGCAGCAGTCTACGCTGCAGCTTCGGCCCTCCCGGCCAACGAGACTCTGGAGGTGGGCGCCAGCTGCACCA---------TCGGTCAGGTCAGCTCGGTGCTGAACAGCTGCTCCAACATCGT
Z6           CTAGCATTCGCGGTAATCGCAGCAGTCTACGCTGCAGCTTCGGCCTTCCCGGCCAACGAGACTCTGGAGGTGGGCGCCAGCTGCACCA---------TCGGTCAGGTCAGCTCGGTGCTGAACAGCTGCTCCAACATCGT
Z6           CTAGCATTCGCGGTAATCGCAGCAGTCTACGCTGCAGCTTCGGCCTTCCCGGCCAACGAGACTCTGGAGGTGGGCGCCAGCTGCCCCA---------TCGGTCAGGTCAGCTCGGTGCTGAACAGCTGCTCCAACATCGT
Z6           CTAGCATTCGCGGTGATCGCAGCAGTCTACGCTGCAGCTTCGGCCTTCCCGGCCAACGAGACTCTGGAGGTGGGCGCCAGCTGC-----------CTTCGGTCAGGTCAGCTCGGTGCTGAACAGCTGCTCCAACATCGT
Z6           CTAGCATTCGCGGTGATCGCAGCAGTCTACGCTGCAGCTTCGGCCTTCCCGGCCAACGAGACTCTGGAGGTGGGCGCCAGCTGC-----------CTTCGGTCAGGTCAGCTCGGTGCTGAACAGCTGCTCCAACATCGT
Z6           CTAGCATTCGCGGTGATCGCAGCAGTCTACGCTGCAGCTTCGGCCTTCCCGGCCAACGAGACTCTGGAGGTGGGCGCCAGCTGC-----------CTTCGGTCAGGTCAGCTCGGTGCTGAACGGCTGCTCCAACATCGT
Z6           CTAGCATTCGCGGTAATCGCAGCAGTCTACGCTGCAGCTTCGGCCTTCCCGGCCAACGAGACTCTGGAGGTGGGCGCCAGCTGCACCA---------TCGGTCAGGTCAGCTCGGTGCTGAACAGCTGCTCCAACATCGT
Z7           CTAGCATTCGCGGTAATCGCAGCAGTCTACGCTGCAGCTTCGGCCTTCCCGGCCAACGAGACTCTGGAGGTGGGCGCCAGCTGCACCA---------TCGGTCAGGTCAGCTCGGTGCTGAACAGCTGCTCCAACATCGT
Z7           CTAGCATTCGCGGTAATCGCAGCAGTCTACGCTGCAGCTTCGGCCTTCCCGGCCAACGAGACTCTGGGGGTGGGCGCCAGCTGCACCA---------TCGGTCAGGTCAGCTCGGTGCTGAACAGCAGCTCCAACATCGT
Z7           CTAGCATTCGCGGTGATCGCAGCAGTCTACGCTGCAGCTTCGGCCTTCCCGGCCAACGAGACTCTGGAGGTGG-CGCCCGCTGC-----------CTTCGGTCAGGTCAGCTCGGTGCTGAACAGCTGCTCCAGCATCGT
Z7           CTAGCATTCGCGGTGATCGCAGCAGTCTACGCTGCAGCTTCGGCCTTCCCGGCCAACGAGACTCTGGAGGTGGGCGCCAGCTGC-----------CTTCGGTCAGGTCAGCTCGGTGCTGAACAGCTGCTCCAACATCGT
Z7           CTAGCATTCGCGGTGATCGCAGCAGTCTACGCTGCAGCTTCGGCCTTCCCGGCCAACGAGACTCTGGAGGTGGGCGCCAGCTGC-----------CTTCGGTCAGGTCAGCTCGGTGCTGAACAGCTGCTCCAACATCGT
Z7           CTAGCATTCGCGGTGATCGCAGCAGTCTACGCTGCAGCTACGGCCTTCCCGGCCAACGAGACTCTGGAGGTGGGCGCCAGCTGC-----------CTTCGGTCAGGTCAGCTCGGTGCTGAACAGCTGCTCCAACATCGT
Z7           CTAGCATTCGCGGTGATCGCAGCAGTCTACGCTGCAGCTTCGGCCTTCCCGGCCAACGAGACTCTGGAGGTGGGCGCCAGCTGC-----------CTTCGGTCAGGTCAGCTCGGTGCTGAACAGCTGCTCCAACATCGT
Z8           CTAGCATTCGCGGTAATCGCAGCAGTCTACGCTGCAGCTTCGGCCTTCCCGGCCAACGAGACTCTGGAGGTGGGCGCCAGCTGCACCA---------TCGGTCAGGTCAGCTCGGTGCTGAACAGCTGCTCCAACATCGT
Z8           CTAGCATTCGCGGTAATCGCAGCAGTCTACGCTGCAGCTTCGGCCTTCCCGGCCAACGAGACTCTGGAGGTGGGCGCCAGCTGCACCA---------TCGGTCAGGTCAGCTCGGTGCTGAACAGCTGCTCCAACATCGT
Z8           CTAGCATTCGCGGTAATCGCAGCAGTCTACGCTGCAGCTTCGGCCTTCCCGGCCAACGAGACTCTGGAGGTGGGCGCCAGCTGCACCA---------TCGGTCAGGTCAGCTCGGTGCTGAACAGCTGCTCCAACATCGT
Z8           CTAGCATTCGCGGTGATCGCAGCAGTCTACGCTGCAGCTTCGGCCTTCCCGGCCAACGAGACTCTGGAGGTGGGCGCCAGCTGC-----------CTTCGGTCAGGTCAGCTCGGTGCTGAACAGCTGCTCCAACATCGT
Z8           CTAGCATTCGCGGTGATCGCAGCAGTCTACGCTGCAGCTTCGGCCTTCCCGGCCAACGAGACTCTGGAGGTGGGCGCCAGCTGC-----------CTTCGGTCAGGTCAGCTCGGTGCTGAACAGCTGCTCCAACATCGT
Z8           CTAGCATTCGCGGTGATCGCAGCAGTCTACGCTGCAGCTTCGGCCTTCCCGGCCAACGAGACTCTGGAGGTGGGCGCCAGCTGC-----------CTTCGGTCAGGTCAGCTCGGTGCTGAACAGCTGCTCCAACATCGT
Z8           CTAGCATTCGCGGTGATCGCAGCAGTCTACGCTGCAGCTTCGGCCTTCCCGGCCAACGAGACTCTGGAGGTGGGCGCCAGCTGC-----------CTTCGGTCAGGTCAGCTCGGTGCTGAACAGCTGCTCCAACATCGT
Z8           CTAGCATTCGCGGTGATCGCAGCAGTCTACGCTGCAGCTTCGGCCTTCCCGGCCAACGAGACTCTGGAGGTGGGCGCCAGCTGC-----------CTTCGGTCAGGTCAGCTCGGTGCTGAACAGCTGCTCCAACATCGT
```

D

```
         I  S  N  X  V  V  K  N  Q  D  D  C  V  A  V  N  K  A  Q  H  V  T  I  S  K  L  Q  C  S  G  G  H  G  L  S  L  S  V  G  M  S  K  S  S  K  A  D  N  T  V  S  D
                                                                                          28-5/9E3a target                        28-5/9E3b target
28-9E3   ATCTCTAACACCGTAGTGAAGAATCAGGACGATTGCGTGGCTGTCAACAAGGCCCAGCACGTGACCATCTCGAAGCTGCAGTGCTCAGGCGGCCATGGTCTCAGTCTGTCCGTGGGCATGAGCAAAACGTCCAAGGCAGATAACACGGTGTCTGAT
X1 28-9  ATCTCTAACACCGTAGTGAAGAATCAGGACGATTGCGTGGCTGTCAACAAGGCCCAGCACGTGACCATCTCGAAGCTGCAGTGCTCAGGCGG------------TCTGTCCGTGGGCATGAGCAAATCGTCCAAGGCAGATAACACGGTGTCTGAT
X1       ATCTCTAACACCGTAGTGAAGAATCGGGATGATTGCGTGGCTGTCAACAAGGCCCAGCACGTGACCATCTCGAAGCTGCAGTGCTCAGGCGG------------TCTGTCCGTGGGCATGAGCAAATCGTCCAAGGCAGATAACACGGTGTCTGAT
X1       ATCTCTAATACCGTAGTGAAGAATCAGGACGATTGCGTGGCTGTCAACAAGGCCCAGCACGTGACCATCTCGAAGCTGCAGTGCTCAGGCGG------------TCTGTCCGTGGGCATGAGCAAATCGTCCAAGGCAGATAACACGGTGTCTGAT
X1       ATCTCTAACACCGTAGTGAAGAATCAGGACGATTGCGTGGCTGTCAACAAGGCCCAGCACGTGACCATCTCGAAGCTGCAGTGCTCAGGCGG------------TCTGTCCGTGGGCATGAGCAAATCGTCCAAGGCAGATAACACGGTGTCTGAT
X1 28-10 ATCTCTAACTCCGTAGTGAAGAATCAGGATGATTGCGTGGCTGTCAACAAGGCTCAGCACGTGACCATCTCGAAGCTGCAGTGCTCAGGCGGACACGGTCTCAGTCTGTCCGTGGGCATGAGC----CA-CCAAGGCAGATAACACTGTGTCCGAT
X1       ATCTCTAACTCCGTAGTGAAGAATCAGGATGATTGCGTGGCTGTCAACAAGGCTCAGCACGTGACCATCTCGAAGCTGCAGTGCTCAGGCGGGCACGGTCTCAGTCTGTCCGTGGGCATGAGC----CA-CCAAGGCAGATAACACTGTGTCCGAT
X1       ATCTCTAACTCCGTAGTGAAGAATCAGGATGGTTGCGTGGCTGTCAACAAGGCTCAGCACGTGACCATCTCGAAGCTGCAGTGCTCAGGCGGACACGGTCTCAGTCTGTCCGTGGGCATGAGC----CA-CCAAGGCAGATAACACTGTGTCCGAT
X2       ATCTCTAACACCGTAGTGAAGAATCAGGACGATTGCGTGGCTGTCAACAAGGCCCAGCACGTGACCATCTCGAAGCTGCAGTGCTCAGGCGG------------TCTGTCCGTGGGCATGAGCAAATCGTCCAAGGCAGATAACACGGTGTCTGAT
X2       ATCTCTAACACCGTAGTGAAGAATCAGGACGATTGCGTGGCTGTCAACAAGGCCCAGCACGTGACCATCTCGAAGCTGCAGTGCTCAGGCGG------------TCTGTCCGTGGGCATGAGCAAATCGTCCAAGGCAGATAACACGGTGTCTGAT
X2       ATCTCTAACTCCGTAGTGAAGAATCAGGATGATTGCGTGGCTGTCAACAAGGCTCAGCACGTGACCATCTCGAAGCTGCAGTGCTCAGGCGGACACGGTCTCAGTCTGTCCGTGGGCATGAGC----CA-CCAAGGCAGATAACACTGTGTCCGAT
X2       ATCTCTAACTCCGTAGTGAAGAATCAGGATGATTGCGTGGCTGTCAACAAGGCTCAGCACGTGACCATCTCGAAGCTGCAGTGCTCAGGCGGACACGGGCTCAGTCTGTCCGTGGGCATGAGC----CA-CCAAGGCAGATAACACTGTGTCCGAT
X2       ATCTCTAACTCCGTAGTGAAGAATCAGGATGATTGCGTGGCTGTCAACAAGGCTCAGCACGTGACCATCTCGAAGCTGCAGTGCTCAGGCGGACACGGTCTCAGTCTGTCCGTGGGCATGAGC----CA-CCAAGGCAGATAACACTGTGTCCGAT
X2       ATCTCTAACTCCGTAGTGAAGAATCAGGATGATTGCGTGGCTGTCAACAAGGCTCAGCACGTGACCATCTCGAAGCTGCAGTGCTCAGGCGGACACGGTCTCAGTCTGTCCGTGGGCATGAGC----CA-CCAAGGCAGGTAACACTGTGTCCGAT
X2       ATCTCTAACTCCGTAGTGAAGAATCAGGATGATTGCGTGGCTGTCAACAAGGCTCAGCACGTGACCATTTCGAAGCTGCAGTGCTCAGGCGGACACGGTCTCAGTCTGTCCGTGGGCATGAGC----CA-CCAAGGCAGATAACACTGTGTCCGAT
X3       ATCTCTAACACCGTAGTGAAGAATCAGGACGATTGCGTGGCTGTCAACAAGGCCCAGCACGTGACCATCTCGAAGCTGCAGTGCTCAGGCGG------------TCTGTCCGTGGGCATGAGCAAATCGTCCAGGGCAGATAACACGGTGTCTGAT
X3       ATCTCTAACACCGTAGTGAAGAATCAGGACGATTGCGTGGCTGTCAACAAGGCCCAGCACGTGACCATCTCGAAGCTGCAGTGCTCAGGCGG------------TCTGTCCGTGGGCATGAGCAAATCGTCCAAGGCAGATAACACGGTGTCTGAT
X3       ATCTCTAACACCGTAGTGAAGAATCAGGACGATTGCGTGGCTGTCAACAAGGCCCAGCACGTGACCATCTCGAAGCTGCAGTGCTCAGGCGG------------TCTGTCCGTGGGCATGAGCAAATCGTCCAAGGCAGATAACACGGTGTCTGAT
X3       ATCTCTAACACCGTAGTGAAGAATCAGGACGATTGCGTGGCTGTCAACAAGGCCCAGCACGTGACCATCTCGAAGCTGCAGTGCTCAGGCGG------------TCTGTCCGTGGGCATGAGCAAATCGTCCAAGGCAGATAACACGGTGTCTGAT
X3       ATCTCTAACACTGTAGTGAAGAATCAGGACGATTGCGTGGCTGTCAACAAGGCCCAGCACGTGACCATCTCGAAGCTGCAGTGCTCAGGCGG------------TCTGTCCGTGGGCATGAGCAAATCGTCCAAGGCAGATAACACGGTGTCTGAT
X3       ATCTCTAACACCGTAGTGAAGAATCAGGACGATTGCGTGGCTGTCAACAAGGCCCAGCACGTGACCATCTCGAAGCTGCAGTGCTCAGGCGG------------TCTGTCCGTGGGCATGAGCAAGTCGTCCAAGGCAGATAACACGGTGTCTGAT
X3       ATCTCTAACACCGTAGTGAAGAATCAGGACGATTGCGTGGCTGTCAACAAGGCCCAGCACGTGACCATCTCGAAGCTGCAGTGCTCAGGCGG------------TCTGTCCGTGGGCATGAGCAAATCGTCCAAGGCAGATAACACGGTGTCTGAT
X3       ATCTCTAACTCCGTAGTGAAGAATCAGGATGATTGCGTGGCTGTCAACAAGGCTCAACACGTGACCATCTCGAAGCTGCAGTGCTCAGGCGGACACGGTCTCAGTCTGTCCGTGGGCATGAGC----CA-CCAAGGCAGATAACACTGTGTCCGAT
X4       ATCTCTAACACCGTAGTGAAGAATCAGGACGATTGCGTGGCTGTCAACAAGGCCCAGCACGTGACCATCTCGAAGCTGCAGTGCTCAGGCGG------------TCTGTCCGTGGGCATGAGCAAATCGTCCAAGGCAGATAACACGGTGTCTGAT
X4       ATCTCTAACACCGTAGTGAAGAATCAGGACGATTGCGTGGCTGTCAACAAGGCCCAGCACGTGACCATCTCGAAGCTGCAGTGCTCAGGCGG------------TCTGTCCGTGGGCATGAGCAAATCGTCCAAGGCAGGTAACACGGTGTCTGAT
X4       ATCTCTAACACCGTAGTGAAGAATCAGGACGATTGCGTGGCTGTCAACAAGGCCCAGCACGTGACCATCTCGAAGCTGCAGTGCTCAGGCGG------------TCTGTCCGTGGGCATGAGCAAATCGTCCAAGGCAGATAACACGGTGTCTGAT
X4       ATCTCTAACTCCGTAGTGAAGAATCAGGATGATTGCGTGGCTGTCAACAAGGCTCAGCACGTGACCATCCCGAAGCTGCAGTGCTCAGGCGGACTGGGCCTGGGTCTGTCCGTGGGCATGAGC----CA-CCAAGGCAGATAACCCTGTGTCCGAT
X4       ATCTCTAACTCCGTAGTGAAGAATCAGGATGATTGCGTGGCTGTCAACAAGGCTCAGCACGTGACCATCTCGAAGCTGCAGTGCTCAGGCGGACACGGTCTCAGTCTGTCCGTGGGCATGAGC----CA-CCAAGGCAGATAACACTGTGTCCGAT
X4       ATCTCTAACTCCGTAGTGAAGAATCAGGATGATTGCGTGGCTGTCAACAAGGCTCAGCACGTGACCATCTCGAAGCTGCAGTGCTCAGGCGGACAC-GTCTCAGTCTGTCCGTGGGCATGAGC----CA-CCAAGGCAGATAACACTGTGTCCGAT
X5       ATCTCTAACACCGTAGTGAAGAATCAGGACGATTGCGTGGCTGTCAACAAGGCCCAGCACGTGACCATCTCGAAGCTGCAGTGCTCAGGCGG------------TCTGTCCGTGGGCATGAGCAAATCGTCCAAGGCAGATAACACGGTGTCTGAT
X5       ATCTCTAACACCGTAGTGAAGAATCAGGACGATTGCGTGGCTGTCAACAAGGCCCAGCACGTGACCATCTCGAAGCTGCAGTGCTCAGGCGG------------TCTGTCCGTGGGCATGAGCAAATCGTCCAAGGCAGATAACACGGTGTCTGAT
X5       ATCTCTAACACCGTAGTGAAGAATCAGGACGATTGCGTGGCTGTCAACAAGGCCCAGCACGTGACCATCTCGAAGCTGCAGTGCTCAGGCGG------------TCTGTCCGTGGGCATGAGCAAATCGTCCAAGGCAGATAACACGGTGTCTGAT
X5       ATCTCTAACACCGTAGAGAAGAATCAGGACGATTGCGTGGCTGTCAACAAGGCCCAGCACGTGACCATCTCGAAGCTGCAGTGCTCAGGCGG------------TCTGTCCGTGGGCATGAGCAAATCGTCCAAGGCAGATAACACGGTGTCTGAT
X5       ATCTCTAACACCGTAGTGAAGAATCAGGACGATTGCGTGGCTGTCAACAAGGCCCAGCACGTGACCATCTCGAAGCTGCAGTGCTCAGGCGG------------TCTGTCCGTGGGCATGAGCAAATCGTCCAAGGCAGATAACACGGTGTCTGAT
X5       ATCTCTAACTCCGTAGTGAAGAATCAGGATGATTGCGTGGCTGTCAACAAGGCTCAGCACGTGACCATCTCGAAGCTGCAGTGCTCAGGCGGACACGGTCTCAGTCTGTCCGTGGGCATGAGC----CA-CCAAGGCAGATAACACTGTGTCCGAT
X6       ATCTCTAACACCGTAGTGAAGAATCAGGACGATTGCGTGGCTGTCAACAAGGCCCAGCACGTGACCATCTCGAAGCTGCAGTGCTCAGGCGG------------TCTGTCCGTGGGCATGAGCAAATCGTCCAAGGCAGATAACACGGTGTCTGAT
X6       ATCTCTAACACCGTA-TGAAAAATCAGGACGATTGCGTGGCTGTCAACAAGGCCCAGCACGTGACCATCTCGAAGCTGCAGTGCTCAGGCGG------------TCTGTCCGCGGGCATGAGCAAATCGTCCAAGGCAGATAACACGGTGTCTGAT
X6       ATCTCTAACTCCGTAGTGAAGAATCAGGATGATTGCGTGGCTGTCAACAAGGCTCAGCACGTGACCATCTCGAAGCTGCAGTGCTCAGGCGGACACGGTCTCAGTCTGTCCGTGGGCATGAGC----CA-CCAAGGCAGATAACACTGTGTCCGAT
X6       ATCTCTAACTCCGTAGTGAAGAATCAGGATGATTGCGTGGCTGTCAACAAGGCTCAGCACGTGACCATCTCGAAGCTGCAGTGCTCAGGCGGACACGGTCTCAGTCTGTCCGTGGGCATGAGC----CA-CCAAGGCAGATAACACGGTGTCTGAT
X6       ATCTCTAACTCCGTAGTGTAGAATCAGGATGATTGCGTGGCTGTCAACAAGGCTCAGCACGTGACCATCTCGAAGCTGCAGTGCTCAGGCGGACACGGTCTCAGTCTGTCCGTGGGCATGAGC----CA-CCAAGGCAGATAACACTGTGTCCGAT
X6       ATCTCTAACTCCGTAGTGAAGAATCAGGATGATTGCGTGGCTGTCAACAAGGCTCAGCACGTGTCCATCTCGAAGCTGCAGTGCTCAGGCGGACACGGTCTCAGTCTGTCCGTGGGCATGAGC----CA-CCAAGGCAGATAACACTGTGTCCGAT
X6       ATCTCTAACTCCGTAGTGAAGAATCAGGATGATTGCGTGGCTGTCAACAAGGCTCAGCACGTGACCATCTCGGAGCTGCAGTGCTCAGGCGGACACGGTCTCAGTCTGTCCGTGGGCATGAGC----CA-CCAAGGCAGATAACACTGTGTCCGAT
X7       ATCTCTAACACCGTAGTGAAGAATCAGGACGATTGCGTGGCTGTCAACAAGGCCCAGCACGTGACCATCTCGAAGCTGCAGTGCTCAGGCGG------------TCTGTCCGTGGGCATGAGCAAATCGTCCAAGGCAGATAACACGGTGTCCGAT
X7       ATCTCTAACACCGTAGTGAAGAATCAGGACGATTGCGTGGCTGTCAACAAGGCCCAGCACGTGACCATCTCGAAGCTGCAGTGCTCAGGCGG------------TCTGTCCGTGGGCATGAGCAAATCGTCCAAGGCAGATAACACGGTGTCTGAT
X7       ATCTCTAACTCCGTAGTGAAGAATCAGGATGATTGCGTGGCTGTCAACAAGGCTCAGCACGTGACCATCTCGAAGCTGCAGTGCTCAGGCGGACACGGTCTCAGTCTGTCCGTGGGCATGAGC----CA-CCAAGGCAGATAACACTGTGTCCGAT
X7       ATCTCTAACTCCGTAGTGAAGAATCAGGATGATTGCGTGGCTGTCAACAAGGCTCAGCACGTGACCATCTCGAAGCTGCAGTGCTCAGGCGGACACGGTCTCAGTCTGTCCGTGGGCATGAGC----CA-CCAAGGCAGATAACACTGTGTCCGAT
X8       ATCTCTAACTCCGTAGTGAAGAATCAGGATGATTGCGTGGCCGTCAACAAGGCTCAGCACGTGACCATCTCGAAGCTGCAGTGCTCAGGCGG------------TCTGTCCGTGGGCATGAGCAAATCGTCCAAGGCAGATAACACGGTGTCTGAT
X8       ATCTCTAACACCGTAGTGAAGAATCAGGACGATTGCGTGGCTGTCAACAAGGCCCAGCACGTGACCATCTCGAAGCTGCAGTGCTCAGGCGG------------TCTGTCCGTGGGCATGAGCAAATCGTCCAAGGCAGATAACACGGTGTCTGAT
X8       ATCTCTAACACCGTAGTGAAGAATCAGGACGATTGCGTGGCTGTCAACAAGGCCCAGCACGTGACCATCTCGAAGCTGCAGTGCTCAGGCGG------------TCTGTCCGTGGGCATGAGCAAATCGTCCAAGGCAGATAACACGGTGTCTGAT
X8       ATCTCTAACACCGTAGTGAAGAATCAGGACGATTGCGTGGCTGTCAACAAGGCCCAGCACGTGACCATCTCGAAGCTGCAGTGCTCAGGCGG------------TCTGTCCGTGGGCATGAGCAAATCGTCCAAGGCAGATAACACGGTGTCTGAT
X8       ATCTCTAACACCGTAGTGAAGAATCAGGACGATTGCGTGGCTGTCAACAAGGCCCAGCACGTGACCATCTCGAAGCTGCAGTGCTCAGGCGG------------TCTGTCCGTGGGCATGAGCAAATCGTCCAAGGCAGATAACACGGTGTCTGAT
X8       ATCTCTAACACCGTAGTGAAGAATCAGGACGATTGCGTGGCTGTCAACAAGGCCCAGCACGTGACCATCTCGAAGCTGCAGTGCTCAGGCGG------------TCTGTCCGTGGGCATGAGCAAATCGTCCAAGGCAGATAACACGGTGTCTGAT
X8       ATCTCTAACTCCGTAGTGAAGAATCAGGATGATTGCGTGGCTGTCAACAAGGCTCAGCACGTGACCATCTCGAAGCTGCAGTGCTCAGGCGGACACGGTCTCAGTCTGTCCGTGGGCATGAGC----CA-CCAAGGCAGATAACACTGTGTCCGAT
Y1       ATCTCTAACACCGTAGTGAAGAATCAGGACGATTGCGTGGCTGTCAACAAGGCCCAGCACGTGACCATCTCGAAGCTGCAGTGCTCAGGCGG------------TCTGTCCGTGGGCATGAGCAAATCGTCCTAGGCAGATAACACGGTGTCTGAT
Y1       ATCTCTAACACCGTAGTGAAGAATCAGGACGATTGCGTGGCTGTCAACAAGGCCCAGCACGTGACCATCTCGAAGCTGCAGTGCTCAGGCGG------------TCTGTCCGTGGGCATGAGCAAATCGTCCAAGGCAGATAACACGGTGTCTGAT
Y1       ATCTCTAACACCGTAGTGAAGAATCAGGACGATTGCGTGGCTGTCAACAAGGCCCAGCACGTGACCATCTCGAAGCTGCAGTGCTCAGGCGG------------TCTGTCCGTGGGCATGAGCAAATCGTCCAAGGCAGATAACACGGTGTCTGAT
Y1       ATCTCTAACACCGTAATGAATAATCAGGACGATTGCGTGGCTGTCAACAAGGCCCAGCACGTGACCATCTCGAAGCTGCAGTGCTCAGGCGG------------TCTGTCCGTGGGCATGAGCAAATCGTCCAAGGCAGATAACACGGTGTCCGAT
Y1       ATCTCTAACACCGTAGTGAAGAATCAGGACGATTGCGTGGCTGTCAACAAGGCCCAGCACGTGACCATCTCGAAGCTGCAGTGCTCAGGCGG------------TCTGTCCGTGGGCATGAGCAAATCGTCCAAGGCAGATAACACGGTGTCTGAT
Y1       ATCTCTAACACCGTAGTGAAGAATCAGGACGATTGCGTGGCTGTCAACAAGGCCCAGCACGTGACCATCTCGAAGCTGCAGTGCTCAGGCGG------------TCTGTCCGTGGGCATGAGCAAATCGTCCAAGGCAGATAACACGGTGTCTGAT
Y1       ATCTCTAACTCCGTAGTGAAGAATCAGGATGATTGCGTGGCTGTCAACAAGGCTCAGCACGTGACCATCTCGAAGCTGCAGTGCTCAGGCGGACACGGTCTCAGTCTGTCCGTGGGCATGAGC----CA-CCAAGGCAGATAACACTGTGTCCGAT
Y1       ATCTCTAACTCCGTAGTGAAGAATCAGGATGATTGCGTGGCTGTCAACAAGGCTCAGCACGTGGCCATCTCGAAGCTGCAGTGCTCAGGCGGACACGGTCTCAGTCTGTCCGTGGGCATGAGC----CA-CCAAGGCAGATAACACTGTGTCCGAT
Y2       ATCTCTAACACCGTAGTGAAGAATCAGGACGATTGCGTGGCTGTCAACAAGGCCCAGCACGTGACCATCTCGAAGCTACAGTGCTCAGGCGG------------TCTGTCCGTGGGCATGAGCAAATCGTCCAAGGCAGATAACACGGTGTCTGAT
Y2       ATCTCTAACACCGTAGTGAAGAATCAGGACGATTGCGTGGCTGTCAACAAGGCCCAGCACGTGACCATCTCGAAGCTGCAGTGCTCAGGCGG------------TCTGTCCGTGGGCATGAGCAAATCGTCCAAGGCAGATAACACGGTGTCTGAT
Y2       ATCTCTAACACCGTAGTGAAGAATCAGGACGATTGCGTGGCTGTCAACAAGGCCCAGCACGTGACCATCTCGAAGCTGCAGTGCTCAGGCGG------------TCTGTCCGTGGGCATGAGCAAATCGTCCAAGGCAGATAACACGGTGTCTGAT
Y2       ATCTCTAACACCGTAGTGAAGAATCAGGACGATTGCGTGGCTGTCAACAAGGCCCAGCACGTGACCATCTCGAAGCTGCAGTGCTCAGGCGG------------TCTGTCCGTGGGCATGAGCAAATCGTCCAAGGCAGATAACACGGTGTCTGAT
Y2       ATCTCTAACTCCGTAGTGAAGAATCAGGATGATTGCGTGGCTGTCAACAAGGCCCAGCACGTGACCATCTCGAAGCTGCAGTGCTCAGGCGG------------TCTGTCCGTGGGCATGAGCAAATCGTCCAAGGCAGATAACACGGTGTCTGAT
Y2       ATCTCTAACACCGTAGTGAAGAATCAGGACGATTGCGTGGCTGTCAACAAGGCCCAGCACGTGACCATCTCGAAGCTGCAGTGCTCAGGCGG------------TCTGTCCGTGGGCATGAGCAAATCGTCCAAGGCAGATAACACGGTGTCTGAT
Y2       ATCTCTAACACCGTAGTGAAGAATCAGGACGATTGCGTGGCTGTCAACAAGGCCCAGCACGTGACCATCTCGAAGCTGCAGTGCTCAGGCGG------------TCTGTCCGTGGGCATGAGCAGTACGTCCAAGGCAGATAACACGGTGTCTGAT
Y2       ATCTCTAACTCCGTAGTGAAGAATCAGGATGATTGCGTGGCTGTCAACAAGGCTCAGCACGTGACCATCTCGAAGCTGCAGTGCTCAGGCGGACACGGTCTCAGTCTGTCCGTGGGCGTGAGC----CA-CCAAGGCAGATAACACTGTGTCCGAT
Y3       ATCTCTAACACCGTAGTGAAGAATCAGGACGATTGCGTGGCTGTCAACAAGGCCCAGCACGTGACCATCTCGAAGCTGCAGTGCTCAGGCGG------------TCTGTCCGTGGGCATGAGCAAATCGTCCAAGGCAGATAACACGGTGTCTGAT
Y3       ATCTCTAACACCGTAGTGAAGAATCAGGACGATTGCGTGGCTGTCAACAAGGCCCAGCACGTGACCATCTCGAAGCTGCAGTGCTCAGGCGG------------TCTGTCCGTGGGCATGAGCAAATCGTCCAAGGCAGATAACACGGTGTCTGAT
Y3       ATCTCTAACACCGTAGTGAAGAATCAGGACGATTGCGTGGCTGTCAACAAGGCCCAGCACGTGGCCATCTCGAAGCTGCAGTGCTCAGGCGG------------TCTGTCCGTGGGCATGAGCAAATCGTCCAAGGCAGATAACACGGTGTCTGAT
Y3       ATCTCTAACACCGTAGTGAAGAGTCAGGACGATTGCGTGGCTGTCAACAAGGCCCAGCACGTGACCATCTCGAAGCTGCAGTGCTCAGGCGG------------TCTGTCCGTGGGCATGAGCAAATCGTCCAAGGCAGATAACACGGTGTCTGAT
Y3       ATCTCTAACACCGTAGTGAAGAATCAGGACGATTGCGTGGCTGTCAACAAGGCCCAGCACGTGACCATCTCGAAGCTGCAGTGCTCAGGCGG------------TCTGTCCGTGGGCATGAGCAAATCGTCCAAGGCAGATAACACGGTGTCCGAT
Y3       ATCTCTAACTCCGTAGTGAAGAATCAGGATGATTGCGTGGCTGTCAACAAGGCTCAGCACGTGACCATCTCGAAGCTGCAGTGCTCAGGCGGACACGGTCTCAGTCTGTCCGTGG-CATGAGC----CA-CCAAGGCAGATAACACCGTGTCCGAT
Y4       ATCTCTAACACCGTAGTGAAGAATCAGGACGATTGCGTGGCTGTCAACAAGGCCCAGCACGTGACCATCTCGAAGCTGCAGTGCTCAGGCGG------------TCTGTCCGTGGGCATGAGCAAATCGTCCAAGGCAGATAACACGGTGTCTGAT
Y4       ATCTCTAACACCGTAGTGAAGAATCAGGACGATTGCGTGGCTGTCAACAAGGCCCAGCACGTGACCATCTCGAAGCTGCAGTGCTCAGGCGG------------TCTGTCCGTGGGCATGAGCAAATCGCCCAAGGCAGATAACACGGTGTCTGAT
Y4       ATCTCTAACACCGTAGTGAAGAATCAGGACGATTGCGTGGCTGTCAACAAGGCCCAGCACGTGACCATCTCGAAGCTGCAGTGCTCAGGCGG------------TCTGTCCGTGGGCATGAGCAAATCGTCCAAGGCAGATAACACGGTGTCTGAT
Y4       ATCTCTAACACCGTAGTGAAGAATCAGGACGATTGCGTGGCTGTCAACAAGGCCCAGCACGTGACCATCTCGAAGCTGCAGTGCTCAGGCGG------------TCTGTCCGTGGGCATGAGCAAATCGTCCAAGGCAGATAACACGGTGTCTGAT
Y4       ATCTCTAACACCGTAGTGAAGAATCAGGACGATTGCGTGGCTGTCAACAAGGCCCAGCACGTGACCATCTCGAAGCTGCAGTGCTCAGGCGG------------TCTGTCCGTGGGCATGAGCAAATCGTCCAAGGCAGATAACACGGTGTCTGAT
Y4       ATCTCTAACTCCGTAGTGAAGAATCAGGATGATTGCGTGGCTGTCAACAAGGCTCAGCACGTGACCATCTCGAAGCTGCAGTGCTCAGGCGGACACGGTCTCAGTCTGTCCGTGGGCATGAGC----CA-CCAAGGCAGATAACACTGTGTCCGAT
Y5       ATCTCTAACACCGTAGTGAAGAATCAGGACGATTGCGTGGCTGTCAACAAGGCCCAGCACGTAACCATCTCGAAGCTGCAGTGCTCAGGCGG------------TCTGTCCGTGGGCATGAGCAAATCGTCCAAGGCAGATAACACGGTGTCTGAT
Y5       ATCTCTAACACCGTAGTGAAGAATCAGGACGATTGCGTGGCTGTCAACAAGGCCCAGCACGTGACCATCTCGAAGCTGCAGTGCTCAGGCGG------------TCTGTCCGTGGGCATGAGCAAATCGTCCAAGGCAGATAACACGGTGTCTGAT
Y5       ATCTCTAACACCGTAGTGAAGAATCAGGACGATTGCGTGGCTGTCAACAAGGCCCAGCACGTGACCATCTCGAAGCTGCAGTGCTCAGGCGG------------TCTGTCCGTGGGCATGAGCAAATCGTCCAAGGCAGATAACACGGTGTCTGAT
Y5       ATCTCTAACACCGTAGTGAAGAATCAGGACGATTGCGTGGCTGTCAACAAGGCCCAGCACGTGACCATCTCGAAGCTGCAGTGCTCAGGCGG------------TCTGTCCGTGGGCATGAGCAAATCGTCCAAGGCAGATAACACGGTGTCTGAT
Y5       ATCTCTAACACCGTAGTGA-GAATCAGGACGATTGCGTGGCTGTCAACAAGGCCCAGCACGTGACCATCTCGAAGCTGCAGTGCTCAGGCGG------------TCTGCCCGTGGGCATGAGCAAATCGTCCAAGGCAGATAACACGGTGTCTGAT
Y5       ATCTCTAACTCCGTAGTGAAGAATCAGGATGATTGCGTGGCTGTCAACAAGGCTCAGCACGTGACCATCTCGAAGCTGCAGTGCTCAGGCGGACACGGTCTCAGTCTGTCCGTGGGCATGAGC----CA-CCAAGGCAGATAACACTGTGTCCGAT
Y5       ATCTCTAACTCCGTAGTGAAGAATCAGGATGATTGCGTGGCTGTCAACAAGGCTCAGCACGTGACCATCTCGAAGCTGCAGTGCTCAGGCGGACACGGTCTCAGTCTGTCCGTGGGCATGAGC----CA-CCAAGGCAGATAACACTGTGTCCGAT
Y5       ATCTCTAACTCCGTAGTGAAGAATCAGGATGATTGCGTGGCTGTCAACAAGGCTCAGCACGTGACCATCTCGAAGCTGCAGTGCTCAGGCGGACACGGTCTCAGTCTGTCCGTGGGCATGAGC----CA-CCAAGGCAGATAACACTGTGTCCGAT
```

D continued

```
             I  S  N  X  V  V  K  N  Q  D  D  C  V  A  V  N  K  A  Q  H  V  T  I  S  K  L  Q  C  S  G  G  H  G  L  S  L  S  V  G  M  S  K  S  S  K  A  D  N  T  V  S  D
                                                                                            28-5/9E3a target                    28-5/9E3b target
28-9E3       ATCTCTAACACCGTAGTGAAGAATCAGGACGATTGCGTGGCTGTCAACAAGGCCCAGCACGTGACCATCTCGAAGCTGCAGTGCTCAGGCGGCCATGGTCTCAGTCTGTCCGTGGGCATGAGCAAAACGTCCAAGGCAGATAACACGGTGTCTGAT
Y6          |ATCTCTAACACCGTAGTGAAGAATCAGGACGATTGCGTGGCTGTCAACAAGGCCCAGCACGTGACCATCTCGAAGCTGCAGTGCTCAGGCGG------------TCTGTCCGTGGGCATGAGCAAATCGTCCAAGGCAGATAACACGGTGTCTGAT
Y6     28-9 |ATCTCTAACACCGTAGTGAAGAATCAGGACGATTGCGTGGCTGTCAACAAGGCCCAGCACGTGACCATCTCGAAGCTGCAGTGCTCAGGCGG------------TCTGTCCGTGGGCATGAGCAAATCGTCCAAGGCAGATAACACGGTGTCTGAT
Y6          |ATCTCTAACACCGTAGTGAAGAATCAGGACGATTGCGTGGCTGTCAACAAGGCCCAGCACGTGACCATCTCGAAGCTGCAGCGCTCAGGCGG------------TCTGTCCGTGGGCATGAGCAAATCGTCCAAGGCAGATAACACGGTGTCTGAT
Y6          |ATCTCTAACACCGTAGTGAAGAATCAGGACGATTGCGTGGCTGTCAACAAGGCCCAGCACGTGACCATCTCGAAGCTGCAGTGCTCAGGCGG------------TCTGTCCGTGGGCATGAGCAAATCGTCCAAGGCAGATAACACGGTGTCTGAT
Y6          |ATCTCTAACACCGTAGTGAAGAATCAGGACGATTGCGTGGCTGTCAACAAGGCCCAGCACGTGACCATCTCGAAGCTGCAGTGCTCAGGCGG------------TCTGTCCGTGGGCATGAGCAAATCGTCCAAGGCAGATAACACGGTGTCTGAT
Y6    28-10 |ATCTCTAACTCCGTAGTGAAGAATCAGGATGATTGCGTGGCTGTCAACAAGGCTCAGCACGTGACCATCTCGAAGCTGCAGTGCTCAGGCGGACACGGTCTCAGTCTGTCCGTGGGCATGAGC----CA-CCAAGGCAGATAACACTGTGTCCGAT
Y7           ATCTCTAACACCGTAGTGAAGAATCAGGACGATTGCGTGGCTGTCAACAAGGCCCAGCACGTGACCATCTCGAAGCTGCAGTGCTCAGGCGG------------TCTGTCCGTGGGCATGAGCAAATCGTCCAAGGCAGATAACACGGTGTCTGAT
Y7           ATCTCTAACACCGTAGTGAAGAATCAGGACGATTGCGTGGCTGTCAACTAGGCCCAGCACGTGACCATCTCGAAGCTGCAGTGCTCAGGCGG------------TCTGTCCGTGGGCATGAGCAAATCGTCCAAGGCAGATAACACGGTGTCTGAT
Y7           ATCTCTAACACCGTAGTGAAGAATCAGGGCGATTGCGTGGCTGTCAACAAGGCCCAGCACGTGACCATCTCGAAGCTGCAGTGCTCAGGCGG------------TCTGTCCGTGGGCATGAGCAAACCGTCCAAGGCAGATAACACGGTGTCTGAT
Y7           ATCTCTAACACCGTAGTGAAGAATCAGGACGATTGCGTGGCTGTCAACAAGGCCCAGCACGTGACCATCTCGAAGCTGCAGTGCTCAGGCGG------------TCTGTCCGTGGGCATGAGCAAATCGTCCAAGGCAGATAACACGGTGTCTGAT
Y7           ATCTCTAACACCGTAGTGAAGAATCAGGACGATTGCGTGGCTGTCAACAAGGCCCAGCACGTGACCATCTCGAAGCTGCAGTGCTCAGGCGG------------TCTGTCCGTGGGCATGAGCAAATCGTCCAAGGCAGAAAACACGGCGTCTGAT
Y7           ATCTCTAACACCGTAGTGAAGAATCAGGACGATTGCGTGGCTGTCAACAAGGCCCAGCACGTGACCATCTCGAAGCTGCAGTGCTCAGGCGG------------TCTGTCCGTGGGCATGAGCAAATCGTCCAAGGCAGATAACACGGTGTCTGAT
Y7           ATCTCTAACACCGTATTGAAGAATCAGGACGATTGCGTGGCTGTCAACAAGGCCCAGCACGTGACCATCTCGAAGCTGCAGTGCTCAGGCGG------------TCTGTCCGTGGGCATGAGCAAATCGTCCAAGGCAGATAACACGGTGTCTGAT
Y7           ATCTCTAACACCGTAGTGAAGAATCAGGACGATTGCGTGGCTGTCAACAAGGCCCAGCACGTGACCATCTCGAAGCTGCAGTGCTCAGGCGG------------CCTGTCCGTGGGCATGAGCAAATCGTCCAAGGCAGATAACACGGTGTCTGAT
Y8           ATCTCTAACACCGTAGTGAAGAATCAGGACGATTGCGTGGCTGTCAACAAGGCCCAGCACGTGACCATCTCGAAGCTGCAGTGCTCAGGCGG------------TCTGTCCGTGGGCATGAGCAAATCGTCCAAGGCAGATAACACGGTGTCTGAT
Y8           ATCTCTAACACCGTAGTGAAGAATCAGGATGATTGCGTGGCTGTCAACAAGGCCCAGCACGTGACCATCTCGAAGCCGCAGTGCTCAGGCGG------------TCTGTCCGTGGGCATGAGCAAATCGTCCATGGCAGATAACACGGTGTCTGTT
Y8           ATCTCTAACACCGTAGTGAAGAATCAGGACGATTGCGTGGCTGTCAACAAGGCCCAGCACGTGACCATCTCGAAGCTGCAGTGCTCAGGCGG------------TCTGTCCGTGGGCATGAGCAAATCGTCCAAGGCAGATAACACGGTGTCTGAT
Y8           ATCTCTAACACCGTAGTGAAGAATCAGGACGATTGCGTGGCTGTCAACAAGGCCCAGCACGTGACCATCTCGAAGCTGCAGTGCTCAGGCGG------------TCTGTCCGTGGGCATGAGCAAATCGTCCAAGGCAGATAACACGGTGTCTGAT
Y8           ATCTCTAACACCGTAGTGAAGAATCAGGACGATTGCGTGGCTGTCAACAAGGCCCAGCACGTGACCATCTCGAAGCTGCAGTGCTCAGGCGG------------TCTGTCCGTGGGCATGAGCAAATCGTCCAAGGCAGATAACACGGTGTCTGAT
Y8           ATCTCTAACTCCGTAGTGAAGAATCAGGATGATTGCGTGGCTGTCAACAAGGCTCAGCACGTGACCATCTCGAAGCTGCAGTGCTCAGGCGGACACGGTCTCAGTCTGTCCGTGGGCATGAGC----CA-CCAAGGCAGATAACACTGTGTCCGAT
Z1           ATCTCTAACACCGCAGTGAAGAATCAGGACGATTGCGTGGCTGTCAACAAGGCCCAGCACGTGACCATCTCGAAGCTGCAGTGCTCAGGCGG------------TCTGTCCGTGGGCATGAGCAAATCGTCCAAGGCAGATGACACGGTGTCTGAT
Z1           ATCTCTAACACCGTAGTGAAGAATCAGGACGATTGCGTGGCTGTCAACAAGGCCCAGCACGTGACCATCTCGAAGCTGCAGTGCTCAGGCGG------------TCTGTCCGTGGGCATGAGCAAATCGTCCAAGGCAGATAACACGGTGTCTGAT
Z1           ATCTCTAACACCGCAGTGAAGAATCAGGACGATTGCGTGGCTGTCAACAAGGCCCAGCACGTGACCATCTCGAAGCTGCAGTGCTCAGGCGG------------TCTGTCCGTGGGCATGAGCAAATCGTCCAAGGCAGATGACACGGTGTCTGAT
Z1           ATCTCTAACACCGTAGTGAAGAATCAGGACGATTGCGTGGCTGTCAACAAGGCCCAGCACGTGACCATCTCGAAGCTGCAGTGCTCAGGCGG------------TCTGTCCGTGGGCATGAGCAAATCGTCCAAGGCAGATAACACGGTGTCTGAT
Z1           ATCTCTAACACCGTAGTGAAGAATCAGGACGATTGCGTGGCTGTCAACAAGGCCCAGCACGTGACCATCTCGAAGCTGCAGTGCTCAGGCGG------------TCTGTCCGTGGGCATGAGCAAATCGTCCAAGGCAGATAACACGGTGTCTGAT
Z1           ATCTCTAACTCCGTAGTGAAGAATCAGGATGATCGCGTGGCTGTCAACAAGGCTCAGCACGTGACCATCTCGAAGCTGCAGTGCTCAGGCGGACACGGTCTCAGTCTGTCCGTGGGCATGAGC----CA-CCAAGGCAGATAACACTGTGTCCGAT
Z1           ATCTCTAACTCCGTAGTGAAGAATCAGGATGATTGCGTGGCTGTCAACAAGGCTCAGCACGTGACCATCTCGAAGCTGCAGTGCTCAGGCGGACACGGTCTCAGTCTGTCCGTGGGCATGAGC----CA-CCAAGGCAGATAACACTGTGTCCGAT
Z2           ATCTCTAACACCGTAGTGAAGAATCAGGACGATTGCGTGGCTGTCAACAAGGCCCAGCACGTGACCATCTCGAAGCTGCAGTGCTCAGGCGG------------TCTGTCCGTGGGCATGAGCAAATCGTCCAAGGCAGATAACACGGTGTCTGAT
Z2           ATCTCTAACACCGTAGTGAAGAATCAGGACGATTGCGTGGCTGTCAACAAGGCCCAGCACGTGACCATCTCGAAGCTGCAGTGCTCAGGCGG------------TCTGTCCGTGGGCATGAGCAAATCGTCCAAGGCAGATAACACGGCGTCTGAT
Z2           ATCTCTAACACCGTAGTGAAGAATCAGGACGATTGCGTGGCTGTCAACAAGGCCCAGCACGTGACCATCTCGAAGCTGCAGTGCTCAGGCGG------------TCTGTCCGTGGGCATGAGCAAATCGTCCAAGGCAGATAACACGGTGTCTGAT
Z2           ATCTCTAACACCGTAGTGAAGAATCAGGACGATTGCGTGGCTGTCAACAAGGCCCAGCACGTGGCCATCTCGAAGCTGCAGTGCTCAGGCGG------------TCTGTCCGTGGGCATGAGCAAATCGTCCAAGGCAGATAACACGGTGTCTGAT
Z2           ATCTCTAACACCGTATTGAAGAATCAGGACGATTGCGTGGCTGTCAACAAGGCCCAGCACGTGACCATCTCGAAGCTGCAGTGCTCAGGCGG------------TCTGTCCGTGGGCATGAGCAAATCGTCCAAGGCAGATAACACGGTGTCTGAT
Z2           ATCTCTAACTCCGTAGTGAAGAATCAGGATGATTGCGTGGCTGTCAACAAGGCTCAGCACGTGACCATCTCGAAGCTGCAGTGCTCAGGCGGACATGGTCTCAGTCTGTCCGTGGGCATGAGC----CA-CCAAGGCAGATAACACTGTGTCCGAT
Z2           ATCTCTAACTCCGTAGTGAAGAATCAGGATGATTGCGTGGCTGTCAACAAGGCTCAGCACGTGACCATCTCGAAGCTGCAGTGCTCAGGCGGACACGGTCTCAGTCTGTCCGTGGGCATGAGC----CA-CCAAGGCAGATAACACTGTGTCCGAT
Z3           ATCTCTAACACCGTAGTGAAGAATCAGGACGATTGCGTGGCTGTCAACAAGGCCCAGCACGTGACCATCTCGAAGCTGCAGTGCTCAGGCGG------------TCTGTCCGTGGGCATGAGCAAATCGTCCAAGGCAGATAACACGGTGTCTGAT
Z3           ATCTCTAACACCGTAGTGAAGAATCAGGACGATTGCGTGGCTGTCAACAAGGCCCAGCACGTGACCATCTCGAAGCTGCAGTGCTCAGGCGG------------TCTGTCCGTGGGCATGAGCAAATCGTCCAAGGCAGATAACACGGTGTCTGAT
Z3           ATCTCTAACACCGTGATGAAGAATCAGGACGATTGCGTGGCTGTCAACAAGGCCCAGAACGTGACCATCTCGAAGCTGCAGTGCTCAGGCGG------------TCTGTCCGTGGGCATGAGCACATCGTCCAAGGAAGATAACACGGTGTCTGAT
Z3           ATCTCTAACTCCGTAGTGAAGAATCAGGATGATTGCGTGGCTGTCAACAAGGCTCAGCACGTGACCATCTCGAAGCTGCAGTGCTCAGGCGGACACGGTCTCAGTCTGTCCGTGGGCATGAGC----CA-CCAAGGCAGATAACACTGTGTCCGAT
Z3           ATCTCTAACTCCGTAGTGAAGAATCAGGATGATTGCGTGGCTGTCAACAAGGCTCAGCACGTGACCATCTCGAAGCTGCAGTGCTCAGGCGGACACGGTCTCAGTCTGTCCGTGGGCATGAGC----CA-CCAAGGCAGATAACACTGTGTCCGAT
Z3           ATCTCTAACTCCGTAGTGAAGAATCAGGATGATTGCGTGGCTGTCAACAAGGCTCAGCACGTGACCATCTCGAAGCTGCAGTGCTCAGGCGGACACGGTCTCAGTCTGTCCGTGGGCATGAGC----CA-CCAAGGCAGATAACACTGTGTCCGAT
Z4           ATCTCTAACACCGTAGTGAAGAATCAGGACGATTGCGTGGCTGTCAACAAGGCCCAGCACGTGACCATCTCGAAGCTGCAGTGCTCAGGCGG------------TCTGTCCGTGGGCATGAGCAAATCGTCCAAGGCAGATAACACGGTGTCTGAT
Z4           ATCTCTAACACCGTAGTGAAGAATCAGGACGATTGCGTGGCTGTCAACAAGGCCCAGCACGTGACCATCTCGAAGCTGCAGTGCTCAGGCGG------------TCTGTCCGTGGGCATGAGCAAATCGTCCAAGGCAGATAACACGGTGTCTGAT
Z4           ATCTCTAACACCGTAGTGAAGAATCAGGACGATTGCGTGGCTGTCAACAAGGCCCAGCACGTGACCATCTCGAAGCTGCAGTGCTCAGGCGG------------TCTGTCCGTGGGCATGAGCAAATCGTCCAAGGCAGATAACACGGTGTCTGAT
Z4           ATCTCTAACACCGTAGTGAAGAATCAGGACGATTGCGTGGCTGTCAACAAGGCCCAGCACGTGACCATCTCGAAGCTGCAGTGCTCAGGCGG------------TCTGTCCGTGGGCATGAGCAAATCGTCCAAGGCAGATAACACGGTGTCTGAT
Z4           ATCTCTAACACCGCAGTGAAGAATCAGGACGATTGCGTGGCTGTCAACAAGGCCCAGCACGTGACCATCTCGAAGCTGCAGCGCTCAGGCGG------------TCTGTCCGTGGGCATGAGCAAATCGTCCAAGGCAGATAACACGGTGTCTGAT
Z4           ATCTCTAACTCCGTAGTGAAGAATCAGGATGATTGCGTGGCTGTCAACAAGGCTCAGCACGTGACCATCTCGAAGCTGCAGTGCTCAGGCGGACACGGTCTCAGTCTGTCCGTGGGCATGAGC----CA-CCAAGGCAGATAACACTGTGTCCGAT
Z4           ATCTCTAACTCCGTAGTGAAGAATCAGGATGATTGCGTGGCTGTCAACAAGGCTCAGCACGTGACCATCTCGAAGCTGCAGTGCTCAGGCGGACACGGTCTCAGTCTGTCCGTGGGCATGAGC----CA-CCAAGGCAGATAACACTGTGTCCGAT
Z4           ATCTCTAACTCCGTAGTGAAGAATCAGGATGATTGCGTGGCTGTCAACAAGGCTCAGCACGTGACCATCTCGAAGCTGCAGTGCTCAGGCGGACACGGTCTCAGTCTGTCCGTGGGCATGAGC----CA-CCAAGGCAGATAACACTGTGTCCGAT
Z5           ATCTCTAACACCGTAGTGAAGAATCAGGACGATTGCGTGGCTGTCAACAAGGCCCAGCACGTGACCATCTCGAAGCTGCAGTGCTCAGGCGG------------TCTGTCCGTGGGCATGAGCAAGTCGTCCAAGGCAGATAACACGGTGTCTGAT
Z5           ATCTCTAACACCGTAGTGAAGAATCAGGACGATTGCGTGGCTGTCAACAAGGCCCAGCACGTGACCATCTCGAAGCTGCAGTGCTCAGGCGG------------TCTGTCCGTGGGCATGAGCAAATCGTCCAAGGCAGATAACACGGTGTCTGAT
Z5           ATCTCTAACACCGTAGTGAAGAATCAGGACGATTGCGTGGCTGTCAACAAGGCCCAGCACGTGACCATCTCGAAGCTGCAGTGCTCAGGCGG------------TCTGTCCGTGGGCATGAGCAAATCGTCCAAGGCAGATAACACGGCGTCTGAT
Z5           ATCTCTAACTCCGTAGTGAAGAATCAGGATGATTGCGTGGCTGTCAACAAGGCTCAGCACGTGACCATCTCGAAGCTGCAGTGCTCAGGCGGACACGGTCTCAGTCTGTCCGTGGGCATGAGC----CA-CCAAGGCAGATAACACTGTGTCCGAT
Z5           ATCTCTAACTCCGTAGTGAAGAATCAGGATGATTGCGTGGCTGTCAACAAGGCTCAGCACGTGACCATCTCGAAGCTGCAGTGCTCAGGCGGACACGGTCTCAGTCTGTCCGTGGGCATGAGC----CA-CCAAGGCAGATAACACTGTGTCCGAT
Z5           ATCTCTAACTCCGTAGTGAAGAATCAGGATGATTGCGTGGCTGTCAACAAGGCTCAGCACGTGACCATCTCGAAGCTGCAGTGCTCAGGCGGACACGGTCTCTGTCTGTCCGTGGGCATGAGC----CA-CCAAGGCAGATAACACTGTGTCCGAT
Z5           ATCTCTAACTCCGTAGTGAAGAATCAGGATGATTGCGTGGCTGTCAACAAGGCTCAGCACGTGACCATCTCGAAGCTGCAGTGCTCAGGCGGACACGGTCTCAGTCTGTCCGTGGGCATGAGC----CA-CCAAGGCAGATAACACCGTGTCCGAT
Z5           ATCTCTAACTCCGTAGTGAAGAATCAGGATAATTGCGTGGCTGTCAACAAGGCTCAGCACGTGACCATCTCGAAGCTGCAGTGCTCAGGCGGACACGGTCTCAGTCTGTCCGTGGGCATGAGC----CA-CCAAGGCAGATAACACTGTGTCCGAT
Z6           ATCTCTAACACCGTAGTGAAGAATCAGGACGATTGCGTGGCTGTCAACAAGGCCCAGCACGTGACCATCTCGAAGCTGCAGTGCTCAGGCGG------------TCTGTCCGTGGGCATGAGCAAATCGTCCAAGGCAGATAACACGGTGTCTGAT
Z6           ATCTCTAACACCGTAGTGAAGAATCAGGACGATTGCGTGGCTGTCAACAAGGCCCAGCACGTGACCATCTCGAAGCTGCAGTGCTCAGGCGG------------TCTGTCCGTGGGCATGAGCAAATCGTCCAAGGCAGATAACACGGTGTCTGAT
Z6           ATCTCTAACTCCGTAGTGAAGAATCAGGATGATTGCGTGGCTGTCAACAAGGCTCAGCACGTGACCATCTCGAAGCTGCAGTGCTCAAGCGGACACGGTCTCAGTCTGTCCGTGGGCATGAGC----CA-CCAAGGCAGATAACACTGTGTCCGAT
Z6           ATCTCTAACTCCGTAGTGAAGAATCAGGATGATTGCGTGGCTGTCAACAAGGCTCAGCACGTGACCATCTCGAAGCTGCAGTGCTCAGGCGGACACGGTCTCAGTCTGTCCGTGGGCATGAGC----CA-CCAAGGCAGATAACACTGTGTCCGAT
Z6           ATCTCTAACTCCGTAGTGAAGAATCAGGATGATTGCGTGGCTGTCAACAAGGCTCAGCACGTGACCATCTCGAAGCTGCAGTGCTCAGGCGGACACGGTCTCAGTCTGTCCGTGGGCATGAGC----CA-CCAAGGCAGATAACACTGTGTCCGAT
Z6           ATCTCTAACTCCGTAGTGAAGAATCAGGATGATTGCGTGGCTGTCAACAAGGCTCAGCACGTGACCATCTCGAAGCTGCAGTGCTCAGGCGGACACGGTCTCAGTCTGTCCGTGGGCATGAGC----CA-CCAAGGCAGATAACACTGTGTCCGAT
Z6           ATCTCTAACTCCGTAGTGAAGAATCAGGATGATTGCGTGGCTGTCAACAAGGCTCAGCACGTGACCATCTCGAAGCTGCAGTGCTCAGGCGGACACGGTCTCAGTCTGTCCGTGGGCATGGGC----CA-CCAAGGCAGATAACACTGCGTCCGAT
Z7           ATCTCTAACACCGTAGTGAAGAATCAGGACGATTGCGTGGCTGTCAACAAGGCCCAGCACGTGACCATCTCGAAGCTGCAGTGCTCAGGCGG------------TCTGTCCGTGGGCATGAGCAAATCGTCCAAGGCAGATAACACGGTGTCTGAT
Z7           ATCTCTAACACCGTAGTGAAGAATCAGGACGATTGCGTGGCTGTCAACAAGGCCCAGCACGTGACCATCTCGAAGCTGCAGTGCTCAGGCGG------------TCTGTCCGTGGGCATGAGCAAATCGTCCAAGGCAGATAACACGGTGTCTGAT
Z7           ATCTCTAACACCGTAGTGAAGAATCAGGACGATTGCGTGGCTGTCAACAAGGCCCAGCACGTGACCATCTCGAAGCTGCAGTGCTCAGGCGG------------TCTGTCCGTGGGCATGAGCAAATCGTCCAAGGCAGATAACACGGTGTCTGAT
Z7           ATCTCTAACACCGTAGTGAAGAATCTGGACGATTGCGTGGCTGTCAACAAGGCCCAGCACGTGACCATCTCGAAGCTGCAGTGCTCAGGCGG------------TCTGTCCGTGGGCATGAGCAAATCGTCCAAGGCAGATAACACGGTGTCTGAT
Z7           ATCTCTAACACCGTAGTGAAGAATCAGGACGATTGCGTGGCTGTCAACAAGGCCCAGCACGTGACCATCTCGAAGCTGCAGTGCTCAGGCGG------------TCTGTCCGTGGGCATGAGCAAATCGTCCAAGGCAGATAACACGGTGTCTGAT
Z7           ATCTCTAACACCGTAGTGAAGAATCAGGACGATTGCGTGGCTGTCAACAAGGCCCAGCACGTGACCATCTCGAAGCTGCAGTGCTCAGGCGG------------TCTGTCCGTGGGCATGAGCAAATCGTCCAAGGCAGATAACACGGTGTCTGAT
Z7           ATCTCTAACTCCGAAGTGAAGAATCAGGATGATTGCGTGGCTGTCAACAAGGCTCAGCACGTGACCATCTCGAAGCTGCAGTGCTCAGGCGGACATGGTCTCAGTCTGTCCGTGGGCATGAGC----CA-CCAAGGCAGATAACACTGTGTCCGAT
Z8           ATCTCTTTCACCGTTGTGAAGAATCAGGACGATTGCGTGGCTGTCAACAAGGCCCAGCACGTGACCATCTCGAAGCTGCAGTGCTCAGGCGG------------TCTGTCCGTGGGCATGAGCAAATCGTCCAAGGCAGATAACACGGTGTCTGAT
Z8           ATCTCTAACACCGTAGTGAAGAATCAGGACGATTGCGTGGCTGTCAACAAGGCCCAGCACGTGACCATCTCGAAGCTGCAGTGCTCAGGCGG------------TCTGTCCGTGGGCATGAGCAAATCGTCCAAGGCAGATAACACGGTGTCTGAT
Z8           ATCTCTAACACCGTAGTGAAGAATCAGGACGATTGCGTGGCTGTCAACAAGGCCCAGCACGTGACCATCTCGAAGCTGCAGTGCTCAGGCGG------------TCTGTCCGTGGGCATGAGCAAATCGTCCAAGGCAGATAACACGGTGTCTGAT
Z8           ATCTCTAACACCGTAGTGAAGAATCAGGACGATTGCGTGGCTGTCAACAAGGCCCAGCACGTGACCATCTCGAAGCTGCAGTGCTCAGGCGG------------TCTGTCCGTGGGCATGAGCAAATCGTCCAAGGCAGATAACACGGTGTCTGAT
Z8           ATCTCTAACACCGTAGTGAAGAATCAGGACGATTGCGTGGCTGTCAACAAGGCCCAGCACGTGACCATCTCGAAGCTGCAGTGCTCAGGCGG------------TCTGTCCGTGGGCATGAGCAAATCGTCCAAGGCAGATAACACGGTGTCTGAT
Z8           ATCTCTAACACCGTAGTGAAGAATCAGGACGATTGCGTGGCTGTCAACAAGGCCCAGCACGTGACCATCTCGAAGCTGCAGTGCTCAGGCGG------------TCTGTCCGTGGGCATGAGCAAATCGTCCAAGGCAGATAACACGGTGTCTGAT
Z8           ATCTCTAACTCCGTAGTGAGGAATCAGGATGATTGCGTGGCTGTCAACAAGGCTCAGCACGTGACCATCTCGAAGCTGCAGTGCTCAGGCGGACACGGTCTCAGTCTGTCCGTGGGCATGAGC----CA-CCAAGGCAGATAGCACTGTGTCCGAT
```

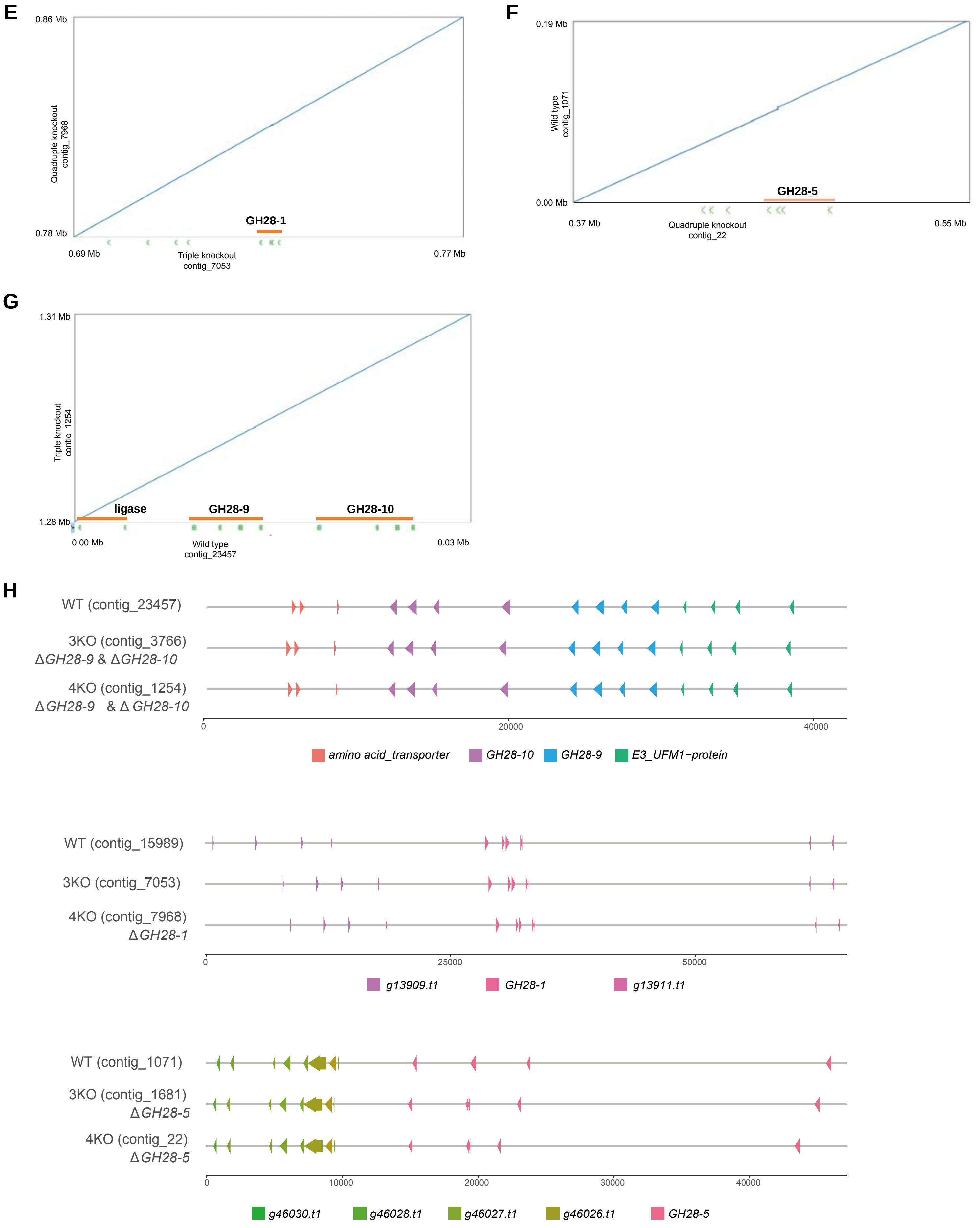

Figure S4 Mutant genotype analysis by either (A-D) multiple nucleotide alignments of *P. cochleariae GH28* amplicons (quadruple mutant) spanning target sites or (E-H) pairwise comparison of genomic regions flanking target sites.
(A-D) PCR products from 24 individuals (X1 to Z8) were cloned and multiple colonies of each genomic region and individual were sequenced. In case of *GH28-1* only colonies from eight individuals were sequenced as the size of the deletion allowed for further verification of the mutation by agarose gel electrophoresis (A bottom). Results verify the consistency of mutations for the loci (A) *GH28-1*, (B) *GH28-5*, (C) *GH28-9* and *10* exon1 and (D) *GH28-9* and *10* exon3. A difference between deletions in *GH28-9* and *10* is obvious. sgRNA target sites as well as translated ORFs of the wild-type sequence are highlighted on top of each alignment.
(E-G) Dot plots illustrating the high sequence similarity of genomic regions containing target *GH28* genes comparing wild type, triple knockout and quadruple knockout contigs from respective genome assemblies. (H) Synteny plots of the same genomic regions illustrating conserved flanking genes of target *GH28* genes comparing wild type, triple knockout and quadruple knockout contigs. These analysis exclude large, unintended deletions.

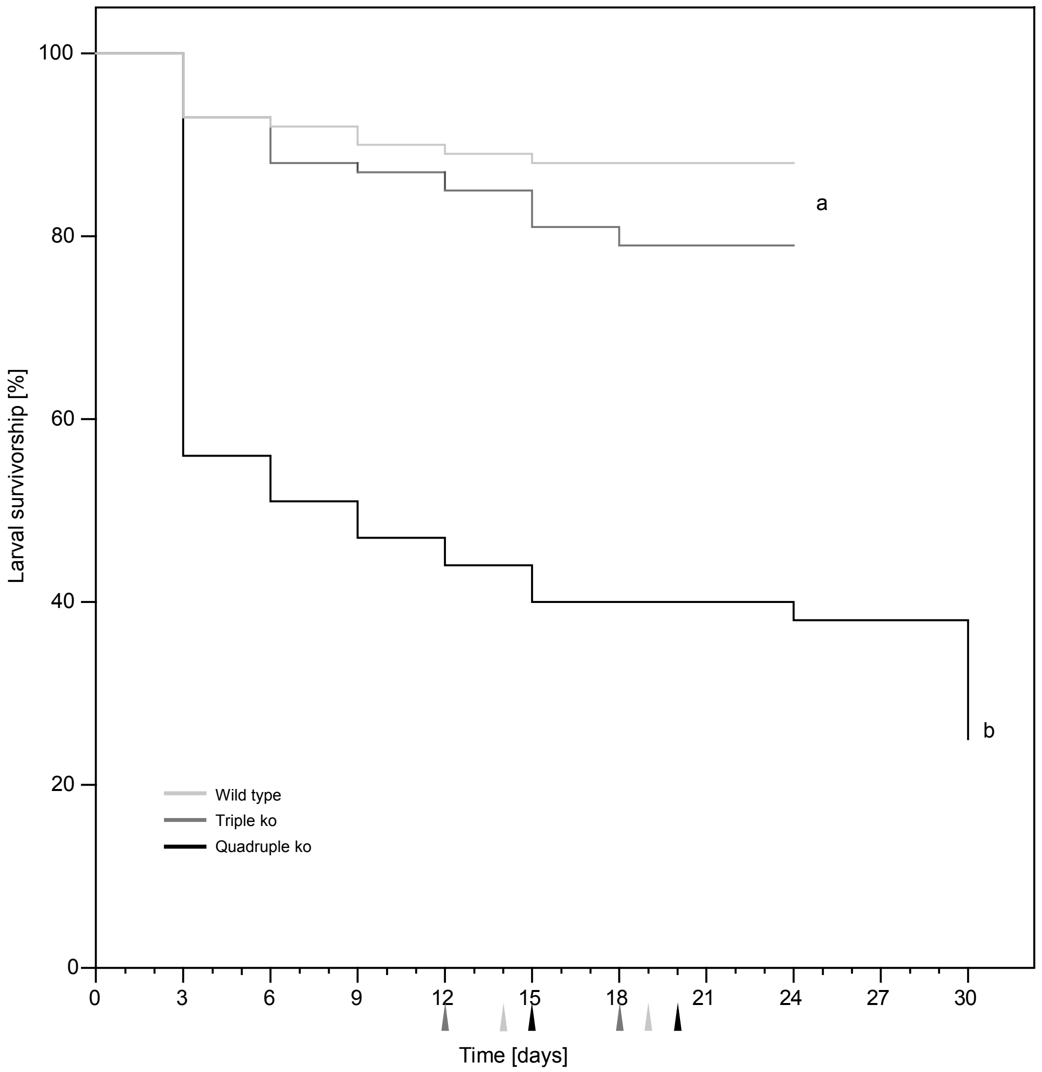

Figure S5 Fitness differences between wild-type (light grey), triple ko (dark grey) and quadruple ko (black) population fed with mature Chinese cabbage leaves from supermarket (standard rearing conditions). Kaplan–Meier survivorship curves of *P. cochleariae* (1st instar larvae to adult eclosion). Arrowheads on the x-axis indicate the beginning of pupation and adult eclosion, respectively. Letters indicate significant differences between populations (n=100, $P<0.001$; Log-Rank Survival Test, χ^2 =113.476).

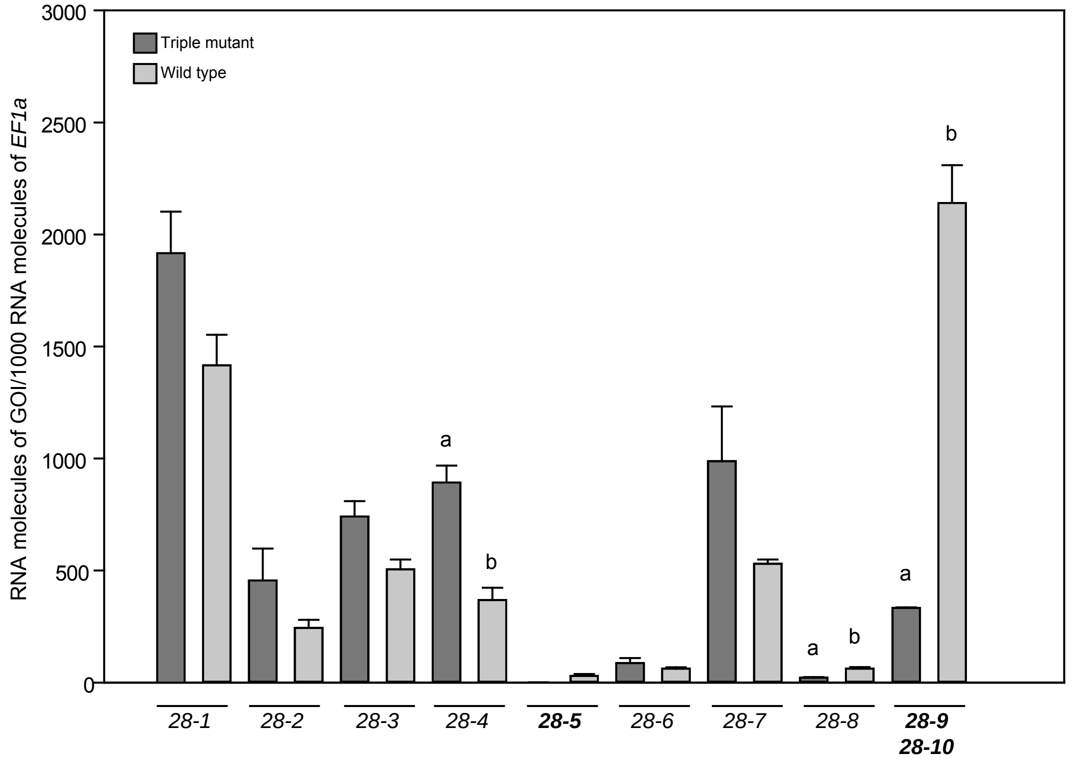

Figure S6 Comparative analysis of GH28 transcript levels between wild-type (light grey) and triple mutant (dark grey) line fed with mature Chinese cabbage leaves from supermarket (standard rearing conditions). Different letters indicate significant differences between lines (for details see table below).
Transcript abundances of GH28 family members are expressed as RNA molecules of gene of interest (GOI) per 1000 RNA molecules of the reference gene *elongation factor 1a* (*EF1a*). Targeted genes in the triple mutant line are highlighted in bold. Signal corresponding to 28-9/28-10 transcripts in the triple mutant was assigned to Δ9/Δ12 28-9 by sequencing the PCR product.

gene	t.test	tvalue	df	FDR
g28_1	Student	2.174	4	0.143
g28_2	Student	1.441	4	0.251
g28_3	Student	2.901	4	0.079
g28_4	Student	5.613	4	0.026
g28_5	Student	-3.559	4	0.053
g28_6	Student	1.109	4	0.330
g28_7	Student	1.869	4	0.174
g28_8	Student	-5.334	4	0.026
g28_9/10	Welch	-10.690	4	0.026

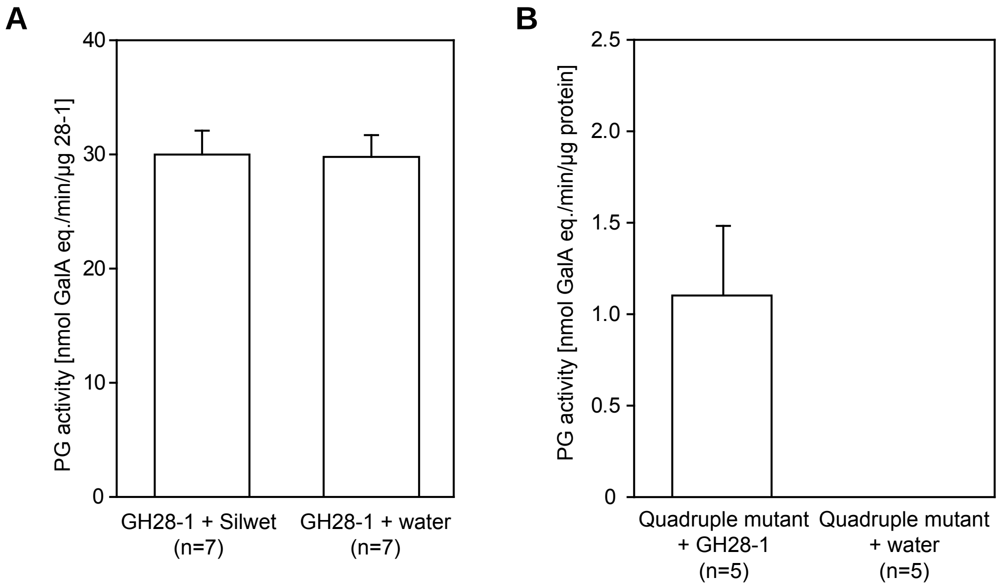

Figure S7 Preliminary test in the context of the rescue experiment shown in Figure 4. Quantification of PG activity of the (A) heterologously expressed GH28 protein with and without Silwet, the detergent reducing the surface tension of aqueous solutions, which was needed to impregnate leaves with GH28 protein (P = 0.020; t-test, t = 2.899) and of the (B) gut content of quadruple mutant larvae after overnight feeding on either leaves with or without GH28-1.

Table S1:

primer name	primer sequence	usage
FAM-M13(-20)F	gtaaaacgacggccagt	FAM labeled forward primer to 5´ lable GH28 diagnostic PCR amplicons for IDAA
PCO_GH28-1E3F	gtaaaacgacggccagtCGACATCAACACCACAGATCAAC	sequencing 28-1 exon3 & IDAA, forward primer (lower case is M13(-20) extension)
PCO_GH28-1E3R	caggaaacagctatgaccCTTCCATGGCAATGTTAC	sequencing 28-1 exon3, reverse primer (lower case is M13(-48mod) extension)
PCO_GH28-1E3R_short	CTTCCATGGCAATGTTACTG	IDAA 28-1 exon3, reverse primer
PCO_GH28-5E1F	gtaaaacgacggccagtTCTCTGTCAAACTCGTAAC	sequencing 28-5 exon1 & IDAA, forward primer (lower case is M13(-20) extension)
PCO_GH28-5E1R	caggaaacagctatgaccTACCGCTGATCTGGATC	sequencing 28-5 exon1, reverse primer (lower case is M13(-48mod) extension)
PCO_GH28-5E1R_short	TACCGCTGATCTGGATC	IDAA 28-5 exon1, reverse primer
PCO_GH28-5E3Fnew	gtaaaacgacggccagtTCTCCTCCAGCCACGAC	sequencing 28-5 exon3 & IDAA, forward primer (lower case is M13(-20) extension)
PCO_GH28-5E3R	caggaaacagctatgaccTTGATGACTCCCGGACC	sequencing 28-5 exon3, reverse primer (lower case is M13(-48mod) extension)
PCO_GH28-5E3R_short	TTGATGACTCCCGGACC	IDAA 28-5 exon3, reverse primer
PCO_GH28-9E1F	gtaaaacgacggccagtGTTCTCCGTCAAACTAGC	sequencing 28-9 exon1 & IDAA, forward primer (lower case is M13(-20) extension)
PCO_GH28-9E1R	caggaaacagctatgaccCATCCCAATGCGCAACTC	sequencing 28-9 exon1, reverse primer (lower case is M13(-48mod) extension)
PCO_GH28-9E1R_short	CATCCCAATGCGCAACTC	IDAA 28-9 exon1, reverse primer
PCO_GH28-9E3Fnew	gtaaaacgacggccagtATCCTCCACCAGCAATGTC	sequencing 28-9 exon3 & IDAA, forward primer (lower case is M13(-20) extension)
PCO_GH28-9E3R	caggaaacagctatgaccCGATGACTCCAGGTCCTC	sequencing 28-9 exon3, reverse primer (lower case is M13(-48mod) extension)
PCO_GH28-9E3R_short	CGATGACTCCAGGTCCTC	IDAA 28-9 exon3, reverse primer
PCO_Fwd_28-9_10E3	TCCTCCACCAGCAATGTCGTCATCTCTAAC	Identification of haplotype frequency (28-9 and 28-10) and (28-9) in wildtype
PCO_Rev_28-9_10E4	TGGAGTTCTTTCCGGTCATCGATCCGGACAC	Identification of haplotype frequency (28-9 and 28-10) and (28-9) in wildtype
crRNA name	crRNA target site sequence	usage
28-1E3a-sgRNA	TTTAACAATTTGGATTGTTCcgg	knockout 28-1
28-1E3b-sgRNA	CATGGTTTGAGTCTGTCAGTtgg	knockout 28-1
28-1E3c-sgRNA	ATCAAAACACATACCAATTCcgg	knockout 28-1
28-5/9E1-sgRNA	AGCTGCACCATCACCAGCTTcgg	knockout 28-5 & 28-9
28-5/9E3a-sgRNA	CTGCAGTGCTCAGGCGGCCAcgg	knockout 28-5 & 28-9
28-5/9E3b-sgRNA	GGGCATGAGCAAAACGTCCAaag	knockout 28-5 & 28-9
